# Health Effects of Occupational and Environmental Exposures to Nuclear Power Plants: A Meta-Analysis and Meta-Regression

**DOI:** 10.1007/s40572-024-00453-8

**Published:** 2024-06-18

**Authors:** Ro-Ting Lin, Hathaichon Boonhat, Yu-Yu Lin, Sonja Klebe, Ken Takahashi

**Affiliations:** 1 Department of Occupational Safety and Health, College of Public Health, China Medical University, Address: No. 100, Sec. 1, Jing-Mao Rd., Beitun Dist., Taichung, 406040 Taiwan; 2 Asbestos Diseases Research Institute, Sydney, NSW 2139 Australia; 3 Department of Epidemiology, Faculty of Public Health, Mahidol University, Bangkok, 10400 Thailand; 4 Graduate Institute of Public Health, College of Public Health, China Medical University, Taichung, 406040 Taiwan; 5 Department of Public Health, College of Public Health, China Medical University, Taichung, 406040 Taiwan; 6 Flinders University, Adelaide, South Australia 5001 Australia; 7 School of Population and Global Health, University of Western Australia, Perth, WA Australia; 8 University of Occupational and Environmental Health, Kitakyushu, Japan; 9 JEOL Ltd, Tokyo, Japan

**Keywords:** Nuclear power plant, Occupational exposure, Environmental exposure, Meta-analysis, Meta-regression, Cancer

## Abstract

**Purpose of Review:**

Numerous epidemiological studies have shown increased health risks among workers and residents living near nuclear power plants exposed to radiation levels meeting regulatory dose limits. This study aimed to evaluate the association between radiation exposure and disease risks among these populations exposed to radiation levels meeting the current regulatory dose limits.

**Recent Findings:**

We searched four databases (Cochrane Library, PubMed, ScienceDirect, and Web of Science) for studies published before August 2023, screened eligible studies (inclusion and exclusion criteria based on population, exposure, comparator, and outcome framework), and collected data on exposure indicators and disease risks. We applied random-effects models of meta-analysis to estimate the pooled effects and meta-regression to assess the dose-response relationship (radiation dose rate for workers and distance for residents). We identified 47 studies, 13 with worker and 34 with resident samples, covering 175 nuclear power plants from 17 countries, encompassing samples of 480,623 workers and 7,530,886 residents. Workers had a significantly lower risk for all-cancer and a significantly higher risk for mesothelioma. Residents had significantly higher risks for all-cancer, thyroid cancer, and leukemia. Notably, children under 5 years old showed the highest risk for all-cancer. Our meta-regression showed a significantly positive dose-response relationship between cumulative dose of radiation exposure and risk for circulatory disease among workers.

**Summary:**

Our findings demonstrated higher risks for mesothelioma for workers and all-cancer, thyroid cancer, and leukemia for residents exposed to low-dose radiation from nuclear power plants. Some included studies did not adjust for cancer risk confounders, which could overestimate the association between radiation exposure and cancer risk and increase the risk of bias.

**Supplementary Information:**

The online version contains supplementary material available at 10.1007/s40572-024-00453-8.

## Introduction

Nuclear power plants can cause immediate injuries through accidents and adverse health effects (e.g., carcinogenicity) because of long-term exposure to low-dose radiation from materials released by power plants [1]. To keep occupational and environmental exposure to radiation levels as low as possible, regulatory dose limits were established by the International Commission on Radiological Protection (ICRP). The recommendations put forth by the ICRP play a critical role in shaping radiological protection policies, regulations, guidelines, and practices worldwide. The ICRP established these regulatory dose limits based on the lifetime risk calculations of cancer. However, the linear no-threshold model proposes the relationship between radiation exposure dose and risks of radiation-induced responses without a threshold [2, 3]. According to the aforementioned model, the ICRP set, in 1990, regulatory dose limits at 100 millisieverts (mSv) per 5 years for workers and 1 mSv per year (mSv/year) for the general public, and these limits remain applicable today [3]. The ICRP’s recommendations on radiation protection have been widely embraced by regulatory bodies and governments worldwide, highlighting its important role in shaping international standards for radiation safety and guiding policy decisions aimed at mitigating radiation-related risks. Nevertheless, there remains the possibility of radiation-induced responses among individuals exposed to levels meeting the current regulatory dose limits.

Three decades have passed since the introduction of these dose limits, and numerous epidemiological studies have shown increased health risks among workers and the general public exposed to radiation levels meeting regulatory dose limits [4–11]. One cohort study with nuclear industry workers in 15 countries showed a significant excess in cancer mortality among workers with exposure to 7.5% of the regulatory limit (i.e., 1.5 mSv/year) [6]. The International Nuclear Workers Study (INWORKS) also demonstrated a significant excess relative risk of 0.47 per Gy [11]. Researchers also showcase increased cancer risks among residents living near nuclear installations [4, 5, 7, 8, 10]. Furthermore, epidemiological studies report on non-cancer disease risks, such as circulatory diseases, after low-dose ionizing radiation [9]. Despite this novel evidence, the regulatory limits set up by the ICRP are based solely on the risk of cancer and heritable effects; uncertainty also remains regarding the dose-response and dose threshold of non-cancer diseases at low doses of radiation exposure [3]. Therefore, there is a need to reassess whether radiation exposure is associated with cancer and non-cancer disease risks among those exposed to levels meeting regulatory dose limits.

This meta-analysis focuses on cancer and non-cancer risks in workers and residents living near nuclear power plants. We defined radiation exposure level at the workplace as an occupational exposure indicator. Due to the absence of actual exposure data for residents living near nuclear power plants and in accordance with previous studies [5, 7], we defined the residential distance of residents from the plants as an environmental exposure indicator. Using a random-effects model of meta-analysis and meta-regression, we investigated the association between exposure levels to radiation from nuclear power plants and disease (cancer and non-cancer) risks among workers and residents. Additionally, we assessed whether these two populations exposed to radiation levels meeting the current regulatory dose limits had an increased disease risk compared to the national population, workers without or with the lowest occupational exposure, and residents living farther from nuclear power plants.

## Materials and Methods

Our meta-analysis followed previous study, the Preferred Reporting Items for Systematic Reviews and Meta-Analyses guidelines, and the Cochrane Handbook for Systematic Reviews of Interventions for the literature search and selection of eligible studies [12–14].

### Search Strategy

We searched four databases, namely the Cochrane Library, PubMed, ScienceDirect, and Web of Science, for studies published before August 10, 2023 [12, 14]. The search terms used to identify the studies on exposure to nuclear power plants and cancer and non-cancer risks were determined based on the population, exposure, comparator, and outcome (PECO) framework [12, 14]. We used the following search terms: “((“nuclear power plant” OR “nuclear site” OR “nuclear power” OR “nuclear facility” OR “nuclear industry” OR “nuclear installation”) NOT (accident OR disaster OR “nuclear power plant incident”)) AND ((resident OR worker) OR (epidemiology OR incidence OR mortality OR death rate OR illness)).” The search terms and filters for the search strategy are presented in Appendix 1. The search comprised articles in any language, including English. All search results were exported to Endnote version 20 (Clarivate, PA, USA), which was used to preliminarily delete duplicate studies.

### Study Selection

Complying with the PECO framework, the following were the inclusion criteria for screening and reviewing full-text papers: (1) populations (P) were workers in nuclear power plants or residents living near such sites; (2) exposures (E) were occupational exposure of workers to radiation from nuclear power plants or environmental exposure of residents living near such sites; (3) comparators (C) were the national population, workers without occupational exposure to radiation of nuclear power plants, workers with the lowest occupational exposure to radiation of such sites, or residents living more than 30 km from nuclear power plants; (4) outcomes (O) were cancer and non-cancer disease incidence and mortality. The exclusion criteria for full-text review were as follows: (1) not focused on occupational or environmental exposure to nuclear power plants in normal operation, such as nuclear accidents at the Chernobyl and Fukushima Daiichi nuclear power plants; (2) combining radiation workers in different employment in analyses (including installations of nuclear weapon and nuclear fuel production), where we could not extract the effect measures of workers only in nuclear power plants; if studies did not only focus on the nuclear power plant, however, they provided effect measures of each facility independently, we included it; (3) containing overlapping populations, and once this occurred, we selected the study with the longest study period to allow sufficient latency periods; (4) lacking effect measures or sufficient exposure data; (5) focused on inheritance, genetics, or cell biology; (6) reviews, letters, or conference abstracts. Then, we used the National Toxicology Program Office of Health Assessment and Translation (NTP/OHAT) risk of bias tool to assess the risk of bias [15]. The NTP/OHAT rated the risk of bias of the studies into three tiers: a study with key elements rated as “definitely low” or “probably low” risk of bias would be categorized as tier 1, “definitely high” or “probably high” as tier 3, and others as tier 2 [15]. Tier 3 studies were excluded from the analysis. If most (more than 60%) information was derived from tier 1 studies, the analysis was evaluated as not likely to have a risk of bias, or if most information was derived from tier 2 studies, the analysis was evaluated to have a serious risk of bias [15].

Two investigators independently screened the titles and abstracts, reviewed the full texts, and evaluated the risk of bias. They discussed and resolved discrepancies in selected studies by discussing the reasons for inclusion or exclusion. A summary of the selection process and agreement rates between the two investigators is shown in Appendix 1.

### Certainty of Evidence

We evaluated the certainty of the evidence of the association between cancer and non-cancer risks and occupational and environmental exposure to radiation in nuclear power plants using the Grading of Recommendations Assessment, Development, and Evaluation (GRADE) approach [12]. The GRADE approach categorizes the certainty of the evidence as “high,” “moderate,” “low,” or “very low” [12]. The details of the judgments of certainty of the evidence are shown in Appendix 2.

### Data Extraction

The following qualitative and quantitative data were extracted: (1) basic information, including the first author’s surname, year of publication, study period, study country or region, study type, study population, sample size, demographic factors (age and sex), and reference population; (2) radiation dose for workers and the average distance from nuclear power plants to residential areas; (3) diseases, without limiting specific diseases but based on whether study groups of diseases were sufficient (at least two study groups); (4) effect measures, including relative risk (RR), odds ratio (OR), standard incidence rate (SIR), and standard mortality rate (SMR). We converted the exposure levels (i.e., average distance in km) and outcomes of OR, SIR, and SMR to RR to interpret the results consistently [12]. The formulae used are shown in Appendix 3. We analyzed three kinds of outcome (i.e., incidence, mortality, and combined incidence and mortality); considering the limited number of studies, we combined incidence and mortality as the main target outcome, and we provided forest plots of pooled estimates of all-cancer incidence and mortality among workers and residents in Appendix 4, respectively.

### Statistical Analysis

We used a random-effects model of meta-analysis to estimate the pooled RR and 95% confidence interval (CI) for occupational and environmental exposure to nuclear power plants and cancer and non-cancer risks. The model was used to incorporate heterogeneity based on the standard errors of individual studies [12]. We applied meta-regression to assess the association between cancer and non-cancer risk and cumulative dose (mSv) of radiation exposure from nuclear power plants for workers and the residential distance (km) from such sites.

For the populations of the different studies that may have different characteristics, we further evaluated the pooled estimate for all-cancer workers and residents in subgroups according to different variables (Appendix 5). The variables for workers included geographic area (America, Asia, and Europe), average cumulative dose of radiation exposure (0–25, 25–50, 50–75, 75–100, and ≥ 100 mSv), average annual cumulative dose of radiation exposure (0–5, 5–10, and ≥ 10 mSv/year), comparator indicators (national population and workers without or with the lowest occupational exposure to radiation), average age at the end of follow-up (< 45 and ≥ 45 years), and the risk of bias (tiers 1 and 2). The variables for residents included geographic area (America, Asia, and Europe), average age of the study sample (0–5, 5–10, and ≥ 10 years or not specified; children over age 10 were grouped with adults for balance analysis), sex (female, male, and not specified), study types (case-control study, cohort study, and ecologic study), exposure indicators (community/district and distance), residential distance from the nuclear power plant (0–10, 10–20, and 20–30 km), and the risk of bias (tiers 1 and 2).

We used the I-squared (*I*^2^) test to investigate the heterogeneity between studies and the leave-one-out method for sensitivity analyses by removing one study group at a time from the original model [12]. We calculated the alternative estimates of the pooled RRs of alternative models and estimated the relative difference between the original and alternative estimates. A relative difference between the original and alternative estimates of < 5.0% was considered a robust result. For publication bias, we used a funnel plot and Egger’s test. Statistical analyses were performed using STATA, version 14. Statistical significance was set at a two-tailed *p* < 0.05.

## Results

**Fig. 1:**
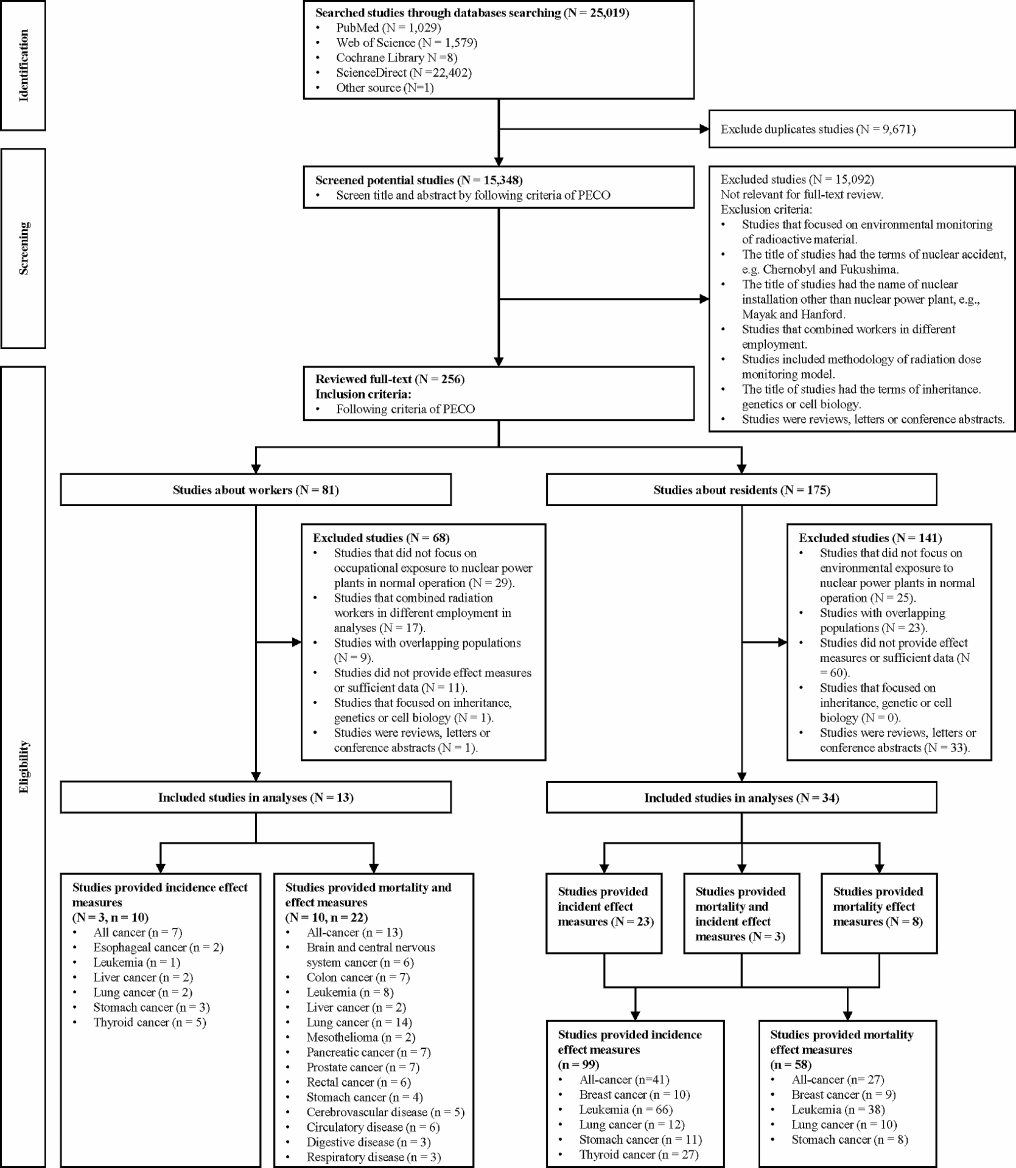
Flow chart of the study selection process *The formula in square brackets means the number of original search studies plus the number of updated search studies. N, number of studies; n, number of study groups included in the meta-analysis; NPP, nuclear power plant; PECO, population, exposure, comparator, and outcome

We identified 25,019 studies in the four databases and one additional source during our review (Fig. 1). After excluding 9,671 duplicates, we screened 15,348 studies by title and abstract. This process led to the exclusion of 15,092 unrelated studies. We classified the 256 studies that were left into two groups: (a) focused on workers in nuclear power plants (*N* = 81); (b) focused on residents living near nuclear power plants (*N* = 175). Sixty-eight and 141 studies on workers and residents, respectively, were excluded in accordance with the exclusion criteria for full-text review. Forty-seven studies were included in the final analysis, with 13 focused on workers and 34 on residents. The summarization of the basic characteristics of included studies and citations is provided in Appendix 6. Our study covered 480,623 workers and 7,530,886 residents from 175 nuclear power plants in 17 countries (Belgium, Canada, Finland, France, Germany, Hungary, Japan, Korea, Lithuania, the Slovak Republic, Slovenia, Spain, Switzerland, Taiwan, Ukraine, the United Kingdom, and the United States of America).

Workers exposed to radiation from nuclear power plants had a lower risk for all-cancer (RR 0.85, 95% CI: 0.75 to 0.97; *p* = 0.013) compared to that of those without or at the lowest occupational exposure level (Table 1). Separating the incidence and mortality outcome, pooled RRs for all-cancer incidence and mortality were 0.88 (95% CI: 0.70 to 1.10; *p* = 0.265) and 0.85 (95% CI: 0.73 to 0.98; *p* = 0.027; Appendix 4). For mesothelioma mortality, we found a significantly higher pooled RR for exposed workers (RR 5.53, 95% CI 4.05 to 7.54; *p* < 0.001) (Table 1). The *I*^2^-value was 58.9% (95% CI: 0.0–81.9%) for all-cancer RRs, representing moderate heterogeneity among these studies. The subgroup analyses for all-cancer types among workers indicated significantly lower risks in Europe (RR 0.79, 95% CI: 0.69 to 0.88; *p* < 0.001), workers with exposure between 25 and 50 mSv (RR 0.71, 95% CI: 0.62 to 0.80; *p* < 0.001), workers exposed to < 5 mSv/year (RR 0.83, 95% CI: 0.71 to 0.97; *p* = 0.019), study groups with < 20 years of follow-up period (RR 0.68, 95% CI: 0.58 to 0.80; *p* < 0.001), workers aged < 45 years (RR 0.83, 95% CI: 0.72 to 0.95; *p* = 0.006), and studies categorized as tier 1 regarding risk of bias (RR 0.81, 95% CI: 0.70 to 0.93; *p* = 0.003); significantly higher risks in workers with exposure between 50 and 75 mSv (RR 1.03, 95% CI: 1.00 to 1.06; *p* = 0.040; Appendix 5). Compared with that of the national population or workers with no or the lowest occupational exposure, those exposed to nuclear power plants showed pooled RRs of 0.87 and 0.81 for all-cancer, respectively (Appendix 5). Meta-regression analyses for workers only showed a significant dose-response relationship between cumulative dose of radiation exposure and risk for circulatory disease (coefficients 0.005, 95% CI: 0.001 to 0.009; *p* = 0.029) (Table 2).

**Table 1:** Pooled relative risk of diseases among workers in nuclear power plants compared with that of the population without or with the lowest occupational exposure to radiation from nuclear power plants

Type of diseases	Number of study groups	Pooled RR (95% CI)	*P*-value^a^	*I*^2^ (95% CI)	*P*-value^b^
**Cancers**					
All-cancer	20	0.85 (0.75–0.97)	0.013	58.9% (0.0–81.9%)	< 0.001
Brain and CNS cancer	6	0.87 (0.75–1.02)	0.088	0.0% (0.0–42.4%)	0.856
Colon cancer	7	0.95 (0.86–1.06)	0.362	0.0% (0.0–41.0%)	0.751
Esophageal cancer	2	1.01 (0.34–3.03)	0.989	0.0% (0.0–79.2%)	0.328
Leukemia	9	1.05 (0.91–1.21)	0.501	0.0% (0.0–39.2%)	0.765
Liver cancer	4	0.42 (0.17–1.00)	0.050^c^	0.0% (0.0–57.8%)	0.523
Lung cancer	16	1.04 (0.85–1.27)	0.691	67.8% (0.0–86.8%)	< 0.001
Mesothelioma	2	5.53 (4.05–7.54)	< 0.001	0.0% (0.0–0.0%)	0.888
Pancreatic cancer	7	0.84 (0.50–1.42)	0.514	49.3% (0.0–81.4%)	0.066
Prostate cancer	7	0.45 (0.18–1.16)	0.097	77.6% (0.0–92.7%)	< 0.001
Rectal cancer	6	1.10 (0.59–2.08)	0.746	22.3% (0.0–70.4%)	0.266
Stomach cancer	7	0.99 (0.83–1.18)	0.918	0.0% (0.0–37.7%)	0.835
Thyroid cancer	5	2.29 (0.76–6.90)	0.141	16.8% (0.0–70.2%)	0.307
**Non-cancer diseases**					
Cerebrovascular disease	5	0.92 (0.75–1.12)	0.390	36.8% (0.0–78.6%)	0.176
Circulatory disease	6	0.93 (0.78–1.11)	0.422	84.6% (0.0–95.5%)	< 0.001
Digestive disease	3	0.78 (0.39–1.59)	0.500	40.3% (0.0–84.4%)	0.187
Respiratory disease	3	0.05 (0.00–2.47)	0.133	91.0% (0.0–97.9%)	< 0.001

^a^*P*-value for the effect of population characteristics

^b^*P*-value of heterogeneity

^c^*P*-value = 0.05003

CI, confidence interval; CNS, central nervous system; *I*^2^, I-squared; RR, relative risk

**Table 2:** Coefficient estimates of risk for cancer or non-cancer disease of workers in nuclear power plants in increasing average cumulative dose of radiation exposure by meta-regression analysis

Type of diseases	Number of study groups	Coefficients (95% CI)	*P*-value^a^	*I*^2^ (%)
**Cancers**				
All-cancer	20	0.002 (-0.003–0.006)	0.488	49.4%
Brain and CNS cancer	6	0.007 (-0.022–0.037)	0.551	0.0%
Colon cancer	7	0.009 (-0.009–0.028)	0.252	0.0%
Esophageal cancer	2	NA	NA	NA
Leukemia	9	0.003 (-0.020–0.027)	0.739	0.0%
Liver cancer	4	0.006 (-0.049–0.062)	0.669	0.0%
Lung cancer	16	-0.000 (-0.011–0.010)	0.926	69.9%
Mesothelioma	2	NA	NA	NA
Pancreatic cancer	7	-0.010 (-0.049–0.030)	0.554	56.4%
Prostate cancer	7	-0.038 (-0.132–0.057)	0.352	81.3%
Rectal cancer	6	0.021 (-0.018–0.059)	0.208	2.8%
Stomach cancer	7	0.017 (-0.038–0.073)	0.308	0.0%
Thyroid cancer	5	-0.005 (-0.038–0.029)	0.699	33.0%
**Non-cancer diseases**				
Cerebrovascular disease	5	0.003 (-0.009–0.015)	0.474	42.4%
Circulatory disease	6	0.005 (0.001–0.009)	0.029	34.5%
Digestive disease	3	-0.014 (-0.226–0.198)	0.562	65.2%
Respiratory disease	3	-0.126 (-0.931–0.680)	0.297	92.0%

^a^*P*-value of coefficient

CI, confidence interval; CNS, central nervous system; *I*^2^, I-squared; NA, not applicable

Residents living within 30 km of the nuclear power plants had a 5% increased all-cancer risk (RR 1.05, 95% CI: 1.00 to 1.09; *p* = 0.045) compared with that of those living farther than 30 km (Table 3). Pooled RRs for all-cancer incidence and mortality were 1.05 (95% CI: 1.00 to 1.09; *p* = 0.047) and 1.02 (95% CI: 0.91 to 1.14; *p* = 0.736), respectively, when the incidence and mortality outcomes were separated (Appendix 4). By cancer type, we found significantly increased risks for thyroid cancer by 17% (RR 1.17, 95% CI: 1.04 to 1.32; *p* = 0.011) and leukemia by 9% (RR 1.09, 95% CI: 1.03 to 1.16; *p* = 0.004; Table 3). The *I*^2^-value was 97.2% (95% CI: 75.6–99.0%) for all-cancer RRs, representing considerable heterogeneity among these studies. The subgroup analyses for all-cancer among residents showed significantly higher risks in America (RR 1.07, 95% CI: 1.00 to 1.13; *p* = 0.046), Asia (RR 1.13, 95% CI: 1.05 to 1.22; *p* = 0.001), residents aged < 5 years (RR 1.09, 95% CI: 1.00 to 1.17; *p* = 0.050), case-control studies (RR 1.20, 95% CI: 1.01 to 1.41; *p* = 0.035), study groups that used distance as the main exposure indicator (RR 1.05, 95% CI: 1.00 to 1.09; *p* = 0.032), and residents living 20–30 km from nuclear power plants (RR 1.08, 95% CI: 1.02 to 1.13; *p* = 0.007; Appendix 5). The meta-regression analyses for residents showed no significant dose-response relationship between distance from nuclear power plants and risk for cancer (Table 4).

**Table 3:** Pooled relative risk of cancers among residents living within 30 km of nuclear power plants compared with that of those living farther than 30 km from nuclear power plants

Type of cancers	Number of study groups	Pooled RR (95% CI)	*P*-value^*^	*I*^2^ (95% CI)	*P*-value^a^
All-cancer	53	1.05 (1.00–1.09)	0.045	97.2% (75.6–99.0%)	< 0.001
Breast cancer	19	1.01 (0.93–1.10)	0.802	90.5% (0.0–97.0%)	< 0.001
Leukemia	102	1.09 (1.03–1.16)	0.004	74.0% (28.2–86.7%)	< 0.001
Lung cancer	22	0.96 (0.86–1.08)	0.529	95.8% (9.8–98.7%)	< 0.001
Stomach cancer	19	0.99 (0.85–1.16)	0.925	90.4% (32.5–96.4%)	< 0.001
Thyroid cancer	27	1.17 (1.04–1.32)	0.011	96.0% (84.2–98.2%)	< 0.001

^a^*P*-value of the effect of population characteristics

^b^*P*-value of heterogeneity

CI, confidence interval; *I*^2^, I-squared; NA, not applicable; RR, relative risk

**Table 4:** Coefficient estimates of risk for cancer of residents living within 30 km of nuclear power plants in increasing average distance from nuclear power plants by meta-regression analysis

Type of cancers	Number of study groups	Coefficients (95% CI)	*P*-value^a^	*I*^2^ (%)
All-cancer	46	0.001 (-0.007–0.009)	0.857	97.3%
Breast cancer	17	-0.021 (-0.059–0.016)	0.247	88.6%
Leukemia	79	-0.008 (-0.018–0.001)	0.073	75.7%
Lung cancer	19	0.006 (-0.029–0.040)	0.735	93.9%
Stomach cancer	17	0.022 (-0.020–0.064)	0.275	87.4%
Thyroid cancer	25	0.019 (-0.022–0.059)	0.351	95.3%

^a^*P*-value of coefficient

CI, confidence interval; *I*^2^, I-squared; NA, not applicable; RR, relative risk

The assessment of risk of bias was in accordance with the NTP/OHAT tool. For the workers, nine studies were categorized as tier 1, and four studies were categorized as tier 2; for the residents, 12 studies were categorized as tier 1 and 22 studies were categorized as tier 2 (Appendix 7). The certainty of evidence of all-cancer among workers and residents was categorized as “very low” based on the *I*^2^-values of 58.9% (95% CI: 0.0–81.9%) and 97.2% (95% CI: 75.6–99.0%), respectively, the potential publication bias (Egger’s test with a *p* < 0.01), and potential risk of bias (not serious in studies with workers and serious in those among residents; Appendix 2). The leave-one-out sensitivity analysis demonstrated that the relative difference between the original and alternative estimates was < 5.0% for all-cancer among workers and residents (Appendix 8).

## Discussion

Our analyses encompassed 480,623 workers in nuclear power plants and 7,530,886 residents living within 30 km of such sites. Workers exposed to radiation levels meeting regulatory dose limits had a significantly lower risk for all-cancer by 0.85-fold but a significantly higher risk for mesothelioma by 5.53-fold. Our meta-regression showed a significantly positive dose-response relationship for circulatory disease among workers. Regarding residents living within 30 km from the site, an exposure of under 1 mSv/year showed significantly increased risks for all-cancer by 5%, thyroid cancer by 17%, and leukemia by 9% compared to those living farther away. Our findings indicate that both workers and residents are still at higher risk for specific cancers, even when exposed to radiation levels meeting the current regulatory dose limits.

Workers had a significantly lower risk for all-cancer. Although the regulatory dose limit for nuclear workers’ radiation exposure is higher than that for the general public, workers are required to wear personal protective equipment during work and enjoy better medical care and greater health-seeking behaviors, which may make them healthier than the general public and decrease disease risk [3, 16]. However, we observed a significantly higher risk for mesothelioma among workers exposed to doses under or near the established limits compared to those without or at the lowest occupational exposure level. Researchers show that workers in nuclear power plants had a high risk of exposure to asbestos from pipe lagging, insulation, gaskets, and some personal protective equipment, such as aprons and gloves, as asbestos fibers were used as insulation materials in most workplaces with high temperatures [17]. Furthermore, our finding for mesothelioma is consistent with that of a previous meta-analysis, revealing a significantly higher risk for mesothelioma (RR 3.57, 95% CI: 2.16–5.89) [18]. Although mesothelioma was thought to be mainly caused by asbestos exposure [19], many studies on patients undergoing radiation therapy or nuclear workers reported an association between ionizing radiation exposure and mesothelioma risk [18, 20, 21]. Regardless of internal or external exposure processes and different ionizing radiation types, ionizing radiation can induce malignant cell transformation [20]. We observed higher but non-significant risks for lung cancer, leukemia, and rectal cancer. Regarding our findings of non-significant differences in risks for non-cancer diseases between workers with exposure, without exposure, or at the lowest level of exposure (Table 1), only four studies were available for analysis, and the results were in line with the ICRP 103 [3], thus suggesting that the dose-response and dose threshold of non-cancer diseases at low dose radiation exposure remain uncertain. Using general populations as the comparator for workers may risk the “healthy worker effect” bias [22]; however, our subgroup analysis of comparator indicators showed the pooled RR of 0.87 among the study groups compared to the national population was non-significantly higher than the pooled RR of 0.81 among the study groups compared to workers with no or the lowest occupational exposure (Appendix 5). We mainly found a significantly positive dose-response relationship for circulatory disease among workers. The mechanisms of radiation-induced circulatory disease cause the vascular smooth muscle to proliferate abnormally, similar to prolonged inflammation, leading the vascular wall to thicken, which is one basic pathological foundation of atherosclerosis [23]. The increasing dose-related trends for circulatory disease are also evidenced in previous studies of nuclear industry workers in 15 countries, although none of them was statistically significant [9].

Based on previous monitoring data [1], the radioactive materials released across the 30 km surrounding the nuclear installations do not expose residents to doses exceeding 0.01 mSv/year [1]. In conjunction with our findings, it seems that the current regulatory limit of 1 mSv/year may not be sufficient to protect the health of residents living within 30 km. In our subgroup analyses, compared with residents living 30 km from nuclear power plants, residents living 20–30 km (and not those living 0–10 km or 10–20 km) from nuclear power plants had a significantly higher risk for all-cancer. Because the airborne radioactive materials released by nuclear power plants have an elevated source, their dispersion may affect the surroundings of the plants by plant stack height, particle size or weight, atmospheric layer structure, or wind [24]. Particularly, the airborne pollutants generally reach a certain height and then start to gradually deposit, which may lead to their concentration not being the highest at the nearest surroundings of the power plants [24]. Our subgroup analyses for all-cancer demonstrated that children would have a higher risk than do adults at the same exposure level to radiation. Compared with adults, premalignant cells in children may have a longer duration to grow and develop more rapidly [25]. The risk of cancer would decrease with an increase in the age at which exposure to radiation occurs [25].

Five previous meta-analyses assess the association between disease risks and radiation exposure. Four of them had similar definitions of occupational and environmental exposure indices as the present study. Below is a comparison of these four meta-analyses and the present study (Appendix 9) [5, 7, 18, 26, 27]. Compared with four previous meta-analyses (two on workers and two on residents) [5, 7, 18, 26], our study covered more comprehensive databases when assessing the potential studies (Appendix 9). Regarding the study population scope, one of the two meta-analyses focused on workers and did not consider workers in nuclear power plants [26], and the other included workers in the broader nuclear industry (i.e., including workers in the mining and nuclear weapon fields) [18]. In contrast, our study specifically focused on workers in nuclear power plants. Regarding the two meta-analyses focused on residents, one only included residents living near nuclear power plants [7], thus being similar to our study, and the other broadly included residents living near different nuclear installations (e.g., nuclear reprocessing, weapon, and uranium mining sites) [5]. Regarding the definition of occupational and environmental exposure indices, the previous four meta-analyses were similar to our study, albeit the exposure levels covered differ by research. Among the meta-analyses focused on workers, Visci et al. did not provide exposure information, while Qu et al. focused on the dose rates of < 5 Sv/year or < 10 mSv/day [18, 26]; meanwhile, we covered occupational exposure between 5 and 200 mSv (0.4–20 mSv/year). The two meta-analyses focused on residents are similar to ours, having used distance from nuclear power plants as an index of environmental exposure [5, 7]. Particularly, we included populations living within 1.56–29 km of nuclear power plants, which is more similar to the sample in Kim et al.’s study (0–30 km) than that in the research by Baker et al. (0–16 km). Regarding outcome variables, the previous four meta-analyses and the present study included cancers, but we did expand the scope by adding non-cancer diseases. Pertaining to statistical methods, most of the six studies used random-effect models and heterogeneity and publication bias tests, whereas only our study evaluated the certainty of evidence using the GRADE approach (Appendex 2).

Our study has some limitations. First, we included studies spanning 175 nuclear power plants from 17 countries; however, as of September 2022, there are 279 nuclear power plants in 34 countries worldwide, based on the International Atomic Energy Agency [28]. Furthermore, most countries we covered are high-income countries (as defined by the World Bank) spanning across Europe, North America, and East Asia, which may have comprehensive radiation protection law systems for workers and residents [29]. Accordingly, our findings may not apply to low-income countries. Second, we speculated that residents living closer to the nuclear power plants would have higher exposure levels and defined distance as the primary exposure indicator; however, lack of information on actual exposure and neglect of the uptake of radioactive materials emitted from nuclear power plants may lead to the underestimation of disease risks. Accordingly, our results could be either over or underestimating the risk because exposure levels can vary at the regional level by time spent outdoors, diet, and wind direction [30]. Third, we excluded studies with overlapping populations and a shorter study period to allow for sufficient latency periods for solid tumors; this process may have led to the exclusion of the latest studies and overestimation of disease risks since it is based on older radiation protection law [31]. However, if the studies provided effect measures for diseases of a combined population of different nuclear industries and did not provide specific information on exposure or effect measures outcome for diseases only related to nuclear power plants, we include another study with a shorter follow-up period (e.g., the United Nations Scientific Committee on the Effects of Atomic Radiation report, and the studies by Zablotska et al. and Haylock et al.) [32–34]. The included studies in our analyses had follow-up periods ranging from 5 to 59 years (mean 21.8 years). Fourth, few studies provided information on some factors which might overestimate the disease risk of radiation exposure. For example, factors of education level or socioeconomic status might have affected the healthy-worker effect on analysis [35]; factor of smoking status might be thought to have a higher risk of some diseases than radiation [19]. Fifth, we found high heterogeneity for some cancer and non-cancer diseases in the random-effects model estimates and meta-regression, particularly among residents, indicating differences in effect sizes across included studies. This may be attributed to different study designs or population characteristics (e.g., age and sex). To address this, we used a random-effects model and applied leave-one-out sensitivity analyses, finding that the relative difference between the original and alternative estimates was less than 5.0% for the cancers discussed, thereby supporting the robustness of our findings (Appendix 8) [12]. Sixth, 43% of the included studies did not adjust for cancer risk confounders, such as the status of smoking and asbestos exposure, and were rated as “probably high” in confounding bias, which was the main reason for them being categorized as tier 2. Still, our subgroup analyses indicated that the estimated RRs in tier 1 and tier 2 studies were similar for all-cancer risks among both workers and residents (Appendix 5). Based on our findings of higher risks for all-cancer, thyroid cancer, and leukemia among residents, we recommended that countries should start assessments on health risks of vulnerable populations. Further research could include individual variables and apply subgroup analyses to precisely evaluate health risks of different populations.

## Conclusion

Our findings suggest that workers are at a higher risk for mesothelioma, even when exposed to radiation levels meeting regulatory dose limits. Although asbestos is the major risk factor for mesothelioma, our study underscores the probable association of mesothelioma and occupational low-dose radiation among workers. Furthermore, we found a significantly positive dose-response relationship for circulatory disease among workers. Importantly, we found higher risks for all-cancer, thyroid cancer, and leukemia among residents living within 30 km of nuclear power plants, especially in children under 5 years old. Our study underscored the need to improve radiation monitoring and protective equipment for workers and residents, focusing on the latter. Notably, some included studies did not adjust for confounding factors related to cancer risk. This may have resulted in a serious risk of bias, particularly in studies focusing on residents, thus potentially leading to an overestimation of the effect of environmental exposure to nuclear power plants on cancer risk.

### Electronic Supplementary Material

Below is the link to the electronic supplementary material.

Supplementary Material 1

## Data Availability

No datasets were generated or analysed during the current study.
