# Supplementary material for: Health Effects of Occupational and Environmental Exposures to Nuclear Power Plants: A Meta-Analysis and Meta-Regression
Source: Curr Environ Health Rep. 2024 Jun 18;11(3):329–39. doi: 10.1007/s40572-024-00453-8 (PMC11324671; doi:10.1007/s40572-024-00453-8)
Supplement: Supplementary file 1 — Supplementary Material 1 [file 40572_2024_453_MOESM1_ESM.pdf]

Supplementary material

Health effects of occupational and environmental exposures to nuclear power plants: A meta-analysis and meta-regression

Table of Contents

Appendix 1 Search strategy 1
Appendix 2 The certainty of evidence 3
Appendix 3 The formulae for data extraction 5
Appendix 4 Forest plots of pooled relative risk for all-cancer 6
Appendix 5 Statistical analysis of subgroup analyses 9
Appendix 6 Summarization of basic characteristics of included studies 11
Appendix 7 Risk of bias 23
Appendix 8 Sensitivity analysis 74
Appendix 9 Comparison with previous meta-analysis 75
Appendix 10 Funnel plot 79
References 80

List of Tables

Table S1. The search terms and filters for search strategy of four databases. 1

Table S2. The summary of findings of evidence using GRADE of all-cancer among workers in nuclear power plants. 4

Table S3. The summary of findings of evidence using GRADE of all-cancer among residents living within 30 km of nuclear power plants. 4

Table S4. Pooled relative risk of subgroup analyses for all-cancer among workers in nuclear power plants. compared with population without or with the lowest occupational exposure to radiation from nuclear power plants. 9

Table S5. Pooled relative risk of subgroup analyses for all-cancer among residents living within 30 km of the nuclear power plants compared with those living farther than 30 km from nuclear power plants.... 10

Table S6. Basic characteristics of the studies for workers in nuclear power plants included in meta-analysis......11

Table S7. Basic characteristics of the studies for residents living near nuclear power plants included in meta-analysis. 15

Table S8. Risk of bias of individual studies of workers in nuclear power plants according the NTP/OHAT risk of bias tool. 24

Table S9. Risk of bias of individual studies of residents living near nuclear power plants according the NTP/OHAT risk of bias tool. 37

Table S10. Summary of the risk of bias of individual studies of workers in nuclear power plants according the NTP/OHAT risk of bias tool. 71

Table S11. Summary of the risk of bias of individual studies of residents living near nuclear power plants according the NTP/OHAT risk of bias tool. 72

Table S12. Summary of sensitivity analysis of pooled estimates on meta-analysis random-effects model for all-cancer among workers in nuclear power plants. 74

Table S13. Summary of sensitivity analysis of pooled estimates on meta-analysis random-effects model for all-cancer among residents living within 30 km of the nuclear power plants. 74

Table S14. The comparison with previous meta-analyses. 75

List of Figures

Figure S1. The summary of agreement rate of screening and selection process. 2
Figure S2. Forest plots of studies on the relative risk of all cancer (a) incidence and (b) mortality among workers in nuclear power plants compared with population without or with the lowest occupational exposure to radiation from nuclear power plants. 6
Figure S3. Forest plots of studies on the relative risk of all-cancer incidence among residents living within 30 km of nuclear power plants compared with those living farther than 30 km from nuclear power plants. 7
Figure S4. Forest plots of studies on the relative risk of all-cancer mortality among residents living within 30 km of nuclear power plants compared with those living farther than 30 km from nuclear power plants. 8
Figure S5. Funnel plot of publication bias of individual studies for all-cancer among workers in nuclear power plants (n = 20). 79
Figure S6. Funnel plot of publication bias of individual studies for all-cancer among residents living within 30 km of nuclear power plants (n = 53). 79

Appendix 1 Search strategy

We originally searched studies published before July 28, 2022 in four databases, namely, the Cochrane Library, PubMed, ScienceDirect, and Web of Science. Updated search were conducted on January 13, 2023 to search studies published from July 29, 2022 to January 13, 2023 and second updated were conducted on August 10, 2023 to search studies published from January 13, 2023 to August 10, 2023 [1].

The search terms and filters for our search strategy are presented in Table S1. As ScienceDirect cannot accommodate more than eight Boolean connective words in one search term, we had to conduct three searches. The second search resulted in more than 6,000 results; this search had to be split into two time periods (1950–2010 and 2011–2022) because ScienceDirect can only display 6,000 results per search.

Table S1. The search terms and filters for search strategy of four databases.

Databases	Search term	Filters for original search	Filters for updated search	Filters for second updated search
**Cochrane Library**	"((“nuclear power plant*” OR “nuclear site*” OR “nuclear power” OR “nuclear facilit*” OR “nuclear industry” OR “nuclear installation*”) NOT (accident* OR disaster* OR “nuclear power plant incident*”)) AND ((residen* OR worker*) OR (epidemiolog* OR incidence* OR mortality OR death rate OR illness))”	Did not apply (searched until July 28, 2022).	Used filter of published years, set from 2022/7 to 2023/2.	Used filter of published years, set from 2023/1 to 2023/9.
**PubMed**	"((“nuclear power plant*” OR “nuclear site*” OR “nuclear power” OR “nuclear facilit*” OR “nuclear industry” OR “nuclear installation*”) NOT (accident* OR disaster* OR “nuclear power plant incident*”)) AND ((residen* OR worker*) OR (epidemiolog* OR incidence* OR mortality OR death rate OR illness))”	Did not apply (searched until July 28, 2022).	Used filter of published years, set from 2022/7/28 to 2023/1/13.	Used filter of published years, set from 2023/1/13 to 2023/8/10.
**ScienceDirect**	(1) (“nuclear power plant” OR “nuclear site”) AND ((residen OR worker) OR (epidemiolog OR incidence OR mortality OR death rate OR illness))	Used research articles as article type filter (searched until July 28, 2022).	Used research articles as article type filter, and used filter of published years, set from 2022 to 2023.	Used research articles as article type filter, and used filter of published years, set from 2023 to 2024.
	(2) (“nuclear power” OR “nuclear facility”) AND ((residen OR worker) OR (epidemiolog OR incidence OR mortality OR death rate OR illness))	(a) Publication year: “1950–2010” and “2011–2022” (b) Used research articles as article type filter. (searched until July 28, 2022)		
	(3) (“nuclear industry” OR “nuclear installation”) AND ((residen OR worker) OR (epidemiolog OR incidence OR mortality OR death rate OR illness))	Used research articles as article type filter (searched until July 28, 2022).		
**Web of Science**	"((“nuclear power plant*” OR “nuclear site*” OR “nuclear power” OR “nuclear facilit*” OR “nuclear industry” OR “nuclear installation*”) NOT (accident* OR disaster* OR “nuclear power plant incident*”)) AND ((residen* OR worker*) OR (epidemiolog* OR incidence* OR mortality OR death rate OR illness))	Did not apply (searched until July 28, 2022).	Used filter of published years, set from 2022/7/28 to 2023/1/13.	Used filter of published years, set from 2023/1/13 to 2023/8/10.

*, can be used to search for different spellings of a word.

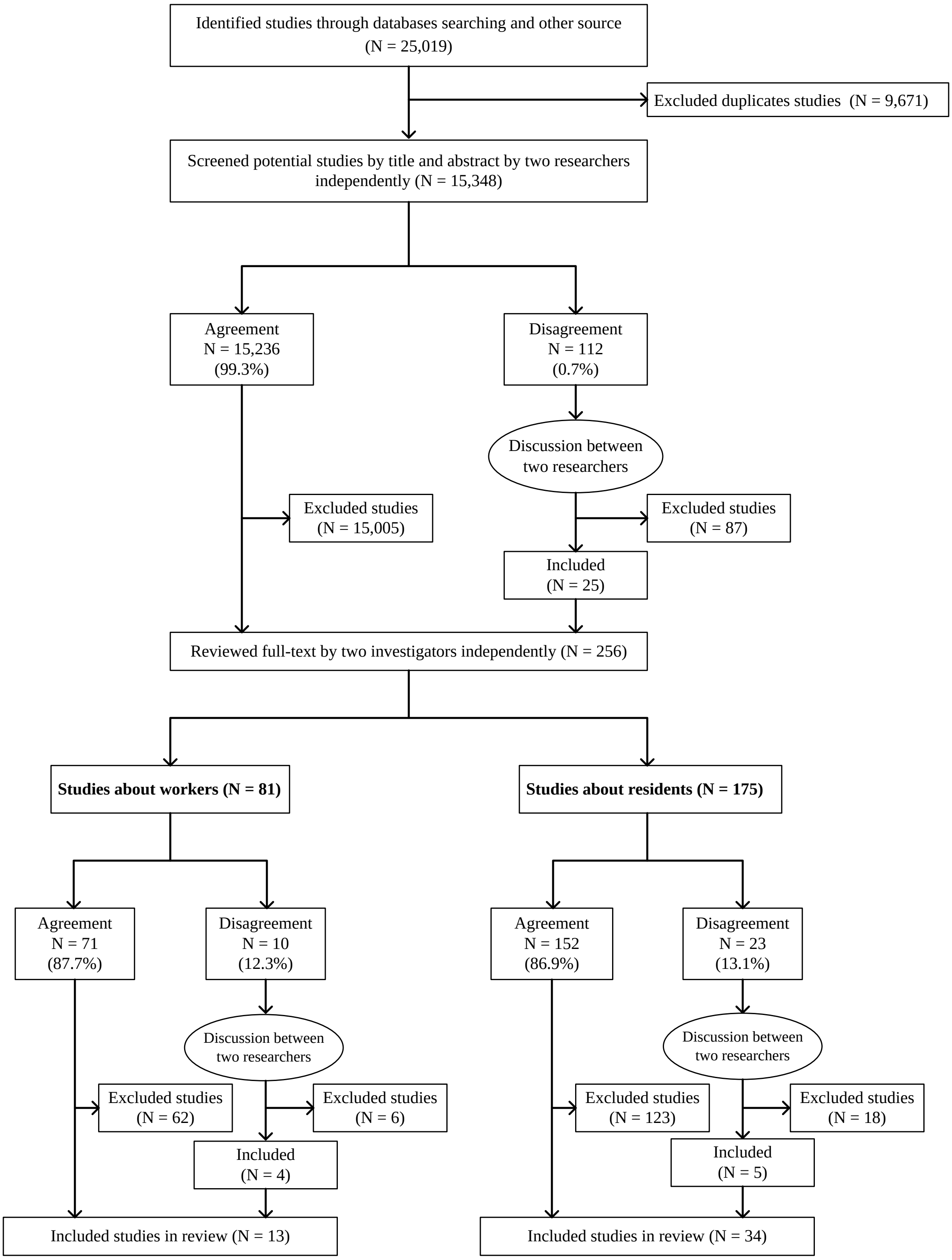

Figure S1. The summary of agreement rate of screening and selection process.
N, number of studies.

Appendix 2 The certainty of evidence

The certainty of evidence is lowered by the risk of bias, inconsistency of the effect, indirectness, imprecision, and publication bias, all of which lower the certainty rating [1]. Ratings are upgraded in the case of a large effect, a dose-response gradient, or plausible residual opposing confounding [1].

The risk of bias rating for studies of all-cancer risk among workers are mostly categorized as tier 1, and that among residents as tiers 1 or 2, indicating that the risks of bias were “not serious” in workers and “serious” in residents. As the meta-analyses of all-cancer risk among workers and residents revealed “moderate” and “considerable” heterogeneity (I^2 = 58.9%–97.2%), respectively, the inconsistencies of the effect were “serious” and “very serious”, respectively. The indirectness and imprecision were “not serious” in all instances because our outcomes directly reflected the nuclear power plant-related health of the workers and residents. Egger’s tests revealed evidence of publication bias for all-cancer risk among workers and residents. We did not detect a significant dose-response relationship between dose or average distance from the nuclear power plants and all-cancer risk for workers or residents. Ultimately, the certainties of evidence for all-cancer risk among workers in nuclear power plants and residents living within 30 km of nuclear power plants were “very low.”

Table S2. The summary of findings of evidence using GRADE of all-cancer among workers in nuclear power plants.

Certainty assessment							Number of patients		Effect	Certainty
**Number of studies**	**Study design**	**Risk of bias**	**Inconsistency**	**Indirectness**	**Imprecision**	**Other considerations**	**Workers in nuclear power plants**	**Reference population**	**Relative risk (RR) (95% CI)**	
8	observational studies	not serious	serious	not serious	not serious	Publication bias: strongly suspected<br>Dose response gradient: no	10,166	10,233	RR: 0.85 (0.75–0.97)	⨁○○○<br>Very low

Table S3. The summary of findings of evidence using GRADE of all-cancer among residents living within 30 km of nuclear power plants.

Certainty assessment							Number of patients		Effect	Certainty
**Number of studies**	**Study design**	**Risk of bias**	**Inconsistency**	**Indirectness**	**Imprecision**	**Other considerations**	**Residents near nuclear power plants**	**Reference population**	**Relative risk (RR) (95% CI)**	
14	observational studies	serious	very serious	not serious	not serious	Publication bias: strongly suspected<br>Dose response gradient: no	167,030	208,892	RR: 1.05 (1.00–1.09)	⨁○○○<br>Very low

GRADE, Grading of Recommendations Assessment, Development, and Evaluation; CI, confidence interval

Appendix 3 The formulae for data extraction

(1) The formula for estimating the exposure indicators

a. We collected data about average cumulative dose (millisieverts [mSv]) and duration of occupational exposure to radiation (years) to calculate the value of average annual cumulative dose (mSv/year). The formula we used is as follows:

$$D\ (\text{mSv/year}) = \frac{d\ (mSv)}{t\ (years)} \qquad \text{(Equation 1)}$$

Where D is average annual cumulative dose (mSv/year), d is average cumulative dose (mSv) and t is duration of occupational exposure to radiation (years).

b. We collected data about average distance from nuclear power plants (km). The formula we used is as follows:

$$d_{\text{average}}\ (\text{km}) = \frac{dmin\ (km) + dmax\ (km)}{2} \qquad \text{(Equation 2)}$$

Where $d_{average}$ is average distance from nuclear power plants (km), d_{min} is the minimum distance of residential range which study had defined near nuclear power plants (km) and d_{max} is the maxium distance of residential range which study had defined near nuclear power plants (km).

(2) The formula for estimating or transforming to relative risk (RR)

As the studies did not directly provide the estimates of RR or presented other outcome indicators, we used the following methods to calculate RR:

a. For studies in which details of the population size were provided, we used the following formula [2]:

$$RR = \frac{\left(\frac{a}{a+b}\right)}{\left(\frac{c}{c+d}\right)} \qquad \text{(Equation 3)}$$

Where a is the number of people who were exposed to nuclear power plants and developed diseases, b is the number of people who were exposed to nuclear power plants but did not develop diseases, c is the number of people who were not exposed to nuclear power plants but developed diseases, and d is the number of people who were not exposed to nuclear power plants and did not develop diseases.

b. The studies provided the value of odds ratio (OR), we use the following formula [3]:

$$RR = \frac{OR}{(1-r)+(r\times OR)} \qquad \text{(Equation 4)}$$

Where r is the incidence rate or mortality rate of diseases in population not exposed to nuclear power plants, and OR is the value of OR from the original article.

If the studies did not provide the SE or 95% CI, we calculated the SE and 95% CI for the natural logarithm of relative risks using following formula [2]:

$$\text{SE of ln RR (SE)} = \sqrt{\frac{1}{a} - \frac{1}{a+c} + \frac{1}{b} - \frac{1}{b+d}} \qquad \text{(Equation 5)}$$

$$\text{95\% CI of ln RR} = \ln RR \pm 1.96 \times SE \qquad \text{(Equation 6)}$$

Where a is the number of people who were exposed to nuclear power plants and developed diseases, b is the number of people who were exposed to nuclear power plants but did not develop diseases, c is the number of people who were not exposed to nuclear power plants but developed diseases, and d is the number of people who were not exposed to nuclear power plants and did not develop diseases.

Appendix 4 Forest plots of pooled relative risk for all-cancer

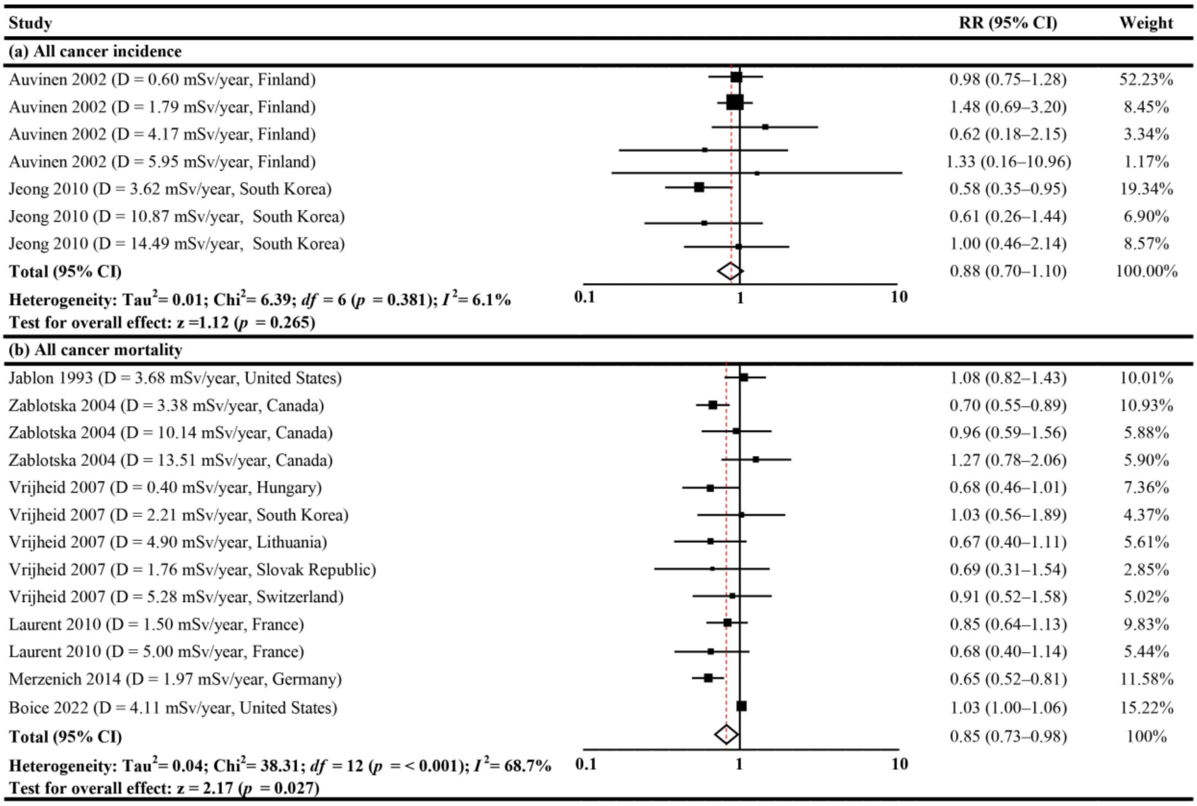

Figure S2. Forest plots of studies on the relative risk of all cancer (a) incidence and (b) mortality among workers in nuclear power plants compared with population without or with the lowest occupational exposure to radiation from nuclear power plants.

D, average annual cumulative dose of radiation exposure workers had exposed (mSv/year); mSv/year, millisievert per year; RR, relative risk; CI, confidence interval; *df*, degrees of freedom; I^2, I-squared; z, Z-score; *p*, P-value.

Study	RR (95% CI)	Weight
**(a) All cancer incidence**		
**Goldsmith 1992** (age = 0–9, Bradwel NPP, United Kingdom)	1.23 (0.73–2.07)	0.60%
**Goldsmith 1992** (age = 0–9, Berkeley NPP, United Kingdom)	1.01 (0.79–1.29)	1.65%
**Goldsmith 1992** (age = 0–9, Sellafield NPP, United Kingdom)	1.81 (1.57–2.09)	2.45%
**Goldsmith 1992** (age = 0–9, Sizewell NPP, United Kingdom)	0.58 (0.52–0.65)	2.67%
Michaelis 1992 (d = 2.5 km, age = 0–4, Germany)	1.42 (1.18–1.72)	2.07%
**Michaelis 1992** (d = 10 km, age = 0–4, Germany)	1.02 (0.97–1.07)	3.17%
Michaelis 1992 (d = 2.5 km, age = 5–14, Germany)	0.90 (0.84–0.97)	3.01%
**Michaelis 1992** (d =1 0 km, age = 5–14, Germany)	0.89 (0.87–0.91)	3.25%
**Mangano 2003** (d = 24 km, Beaver Valley NPP, age = 0–9, United States)	1.04 (0.94–1.15)	2.83%
**Mangano 2003** (d = 24 km, Brookhaven NPP, age = 0–9, United States)	1.04 (0.93–1.16)	2.73%
**Mangano 2003** (d = 24 km, Crystal River NPP, age = 0–9, United States)	1.04 (0.84–1.29)	1.87%
**Mangano 2003** (d = 24 km, Indian Point Valley NPP, age = 0–9, United States)	1.05 (0.93–1.19)	2.63%
**Mangano 2003** (d = 24 km, Limerick NPP, age = 0–9, United States)	1.03 (0.94–1.13)	2.91%
**Mangano 2003** (d = 24 km, Millstone NPP, age = 0–9, United States)	0.92 (0.79–1.07)	2.43%
**Mangano 2003** (d = 24 km, Oyster Creek Valley NPP, age = 0–9, United States)	1.27 (1.13–1.43)	2.68%
**Mangano 2003** (d = 24 km, Pilgrim NPP, age = 0–9, United States)	1.15 (0.96–1.38)	2.15%
**Mangano 2003** (d = 24 km, Salem NPP, age = 0–9, United States)	1.02 (0.89–1.17)	2.51%
**Mangano 2003** (d = 24 km, Seabrook NPP, age = 0–9, United States)	1.08 (0.95–1.22)	2.62%
**Mangano 2003** (d = 24 km, St. Lucie Valley NPP, age = 0–9, United States)	1.45 (1.16–1.82)	1.79%
**Mangano 2003** (d = 24 km, Susquehanna Valley NPP, age = 0–9, United States)	1.13 (0.95–1.34)	2.24%
**Mangano 2003** (d = 24 km, Three Mile Island NPP,age = 0–9, United States)	1.07 (0.96–1.19)	2.74%
**Mangano 2003** (d = 24 km, Turkey Point NPP, age = 0–9, United States)	1.28 (1.18–1.39)	2.96%
**Zadnik 2008** (Females, Krško NPP, Slovenia)	0.99 (0.95–1.03)	3.20%
**Zadnik 2008** (Males, Krško NPP, Slovenia)	0.95 (0.91–0.99)	3.20%
Heinävaara 2010 (Female, d = 7.5 km, age = 0–14, Finland)	1.03 (0.34–3.16)	0.15%
**Heinävaara 2010** (Females, d = 7.5 km, age > 14, Finland)	0.96 (0.92–1.01)	3.17%
Heinävaara 2010 (Male, d = 7.5 km, age = 0–14, Finland)	1.45 (0.76–2.75)	0.42%
Heinävaara 2010 (Males, d = 7.5 km, age > 14, Finland)	0.98 (0.93–1.03)	3.15%
Spycher 2011 (d = 2.5 km, age = 0–15, Switzerland)	1.03 (0.78–1.36)	1.44%
Spycher 2011 (d = 7.5 km, age = 0–15, Switzerland)	0.92 (0.77–1.10)	2.16%
Spycher 2011 (d = 12.5 km, age = 0–15, Switzerland)	1.14 (0.99–1.31)	2.52%
**Bazyka 2012** (Ukraine)	0.72 (0.69–0.75)	3.19%
Ahn 2012 (Females, d = 2.5 km, age = 53, South Korea)	1.09 (0.94–1.26)	2.41%
Ahn 2012 (Female, d = 17.5 km, age = 53, South Korea)	1.22 (1.05–1.42)	2.40%
Ahn 2012 (Male, d = 2.5 km, age = 53, South Korea)	1.02 (0.90–1.16)	2.61%
Ahn 2012 (Males, d = 17.5 km, age = 53, South Korea)	1.10 (0.97–1.24)	2.64%
Lane 2013 (d = 12.5 km, Bruce NPP, Canada)	1.40 (1.35–1.45)	3.22%
Lane 2013 (d = 12.5 km, Darlington NPP, Canada)	0.87 (0.86–0.88)	3.28%
Lane 2013 (d = 12.5 km, Pickering NPP, Canada)	0.87 (0.86–0.88)	3.29%
**Wang 2016** (Females, d = 7 km, Taiwan)	1.40 (1.09–1.80)	1.62%
**Wang 2016** (Male, d = 7 km, Taiwan)	1.18 (0.96–1.45)	1.95%
**Total (95% CI)**	1.05 (1.00–1.09)	100.00%

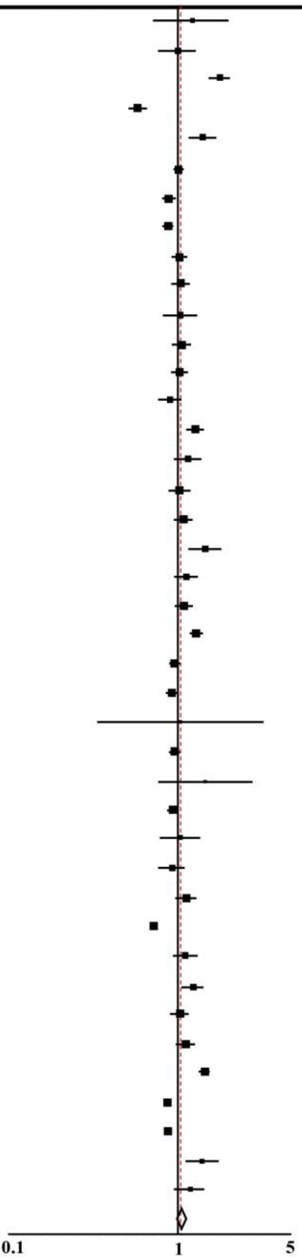

Heterogeneity: Tau^2= 0.02; Chi^2= 1274.29; *df* = 40 (p < 0.001); I^2= 96.9%
Test for overall effect: z = 1.99 (p = 0.047)

Figure S3. Forest plots of studies on the relative risk of all-cancer incidence among residents living within 30 km of nuclear power plants compared with those living farther than 30 km from nuclear power plants. d, average residential distance from nuclear power plants (km); age, average age of population; NPP, nuclear power plant; km, kilometer; RR, relative risk; CI, confidence interval; *df*, degrees of freedom; I^2, I-squared; z, Z-score; *p*, P-value.

Study	RR (95% CI)	Weight
**(b) All cancer mortality**		
**Baron 1984** (d = 4 km, Bradwell NPP, United Kingdom)	1.63 (1.48–1.79)	4.56%
**Baron 1984** (d = 4 km, Berkeleyl NPP, United Kingdom)	1.62 (1.52–1.73)	4.64%
**Baron 1984** (d = 4 km, Dungeness NPP, United Kingdom)	1.11 (0.79–1.55)	3.25%
**Baron 1984** (d = 4 km, Hinkley NPP, United Kingdom)	0.62 (0.55–0.69)	4.49%
**Baron 1984** (d = 4 km, Trawsfynyd NPP, United Kingdom)	0.35 (0.22–0.56)	2.48%
**Goldsmith 1992** (age = 0–9, Sellafield NPP, United Kingdom)	1.10 (0.65–1.86)	2.24%
**López-Abente 2001** (d = 15 km, Almaraz NPP, Spain)	1.09 (0.90–1.33)	4.09%
**López-Abente 2001** (d = 15 km, Garoña NPP, Spain)	0.91 (0.77–1.07)	4.27%
**López-Abente 2001** (d = 15 km, Vandellòs NPP, Spain)	1.05 (0.95–1.16)	4.53%
**López-Abente 2001** (d = 15 km, Zorita NPP, Spain)	1.00 (0.90–1.11)	4.51%
**Silva-Mato 2003** (d = 5 km, Trillo NPP, Spain)	1.29 (1.08–1.55)	4.18%
**Silva-Mato 2003** (d = 15 km, Trillo NPP, Spain)	1.09 (0.89–1.34)	4.04%
**Mangano 2003** (d = 24 km, Beaver Valley NPP, age = 0–9, United States)	0.91 (0.73–1.14)	3.94%
**Mangano 2003** (d = 24 km, Brookhaven NPP, age = 0–9, United States)	0.84 (0.65–1.09)	3.70%
**Mangano 2003** (d = 24 km, Crystal River NPP, age = 0–9, United States)	0.60 (0.33–1.08)	1.97%
**Mangano 2003** (d = 24 km, Indian Point Valley NPP, age = 0–9, United States)	1.01 (0.77–1.32)	3.68%
**Mangano 2003** (d = 24 km, Limerick NPP, age = 0–9, United States)	0.96 (0.79–1.17)	4.10%
**Mangano 2003** (d = 24 km, Millstone NPP, age = 0–9, United States)	0.73 (0.52–1.03)	3.18%
**Mangano 2003** (d = 24 km, Oyster Creek Valley NPP, age = 0–9, United States)	1.30 (1.02–1.66)	3.81%
**Mangano 2003** (d = 24 km, Pilgrim NPP, age = 0–9, United States)	1.02 (0.68–1.52)	2.87%
**Mangano 2003** (d = 24 km, Salem NPP, age = 0–9, United States)	0.99 (0.74–1.33)	3.51%
**Mangano 2003** (d = 24 km, Seabrook NPP, age = 0–9, United States)	1.28 (1.00–1.63)	3.81%
**Mangano 2003** (d = 24 km, St. Lucie Valley NPP, age = 0–9, United States)	1.27 (0.77–2.11)	2.33%
**Mangano 2003** (d = 24 km, Susquehanna Valley NPP, age = 0–9, United States)	1.03 (0.71–1.49)	3.04%
**Mangano 2003** (d = 24 km, Three Mile Island NPP, age = 0–9, United States)	1.32 (1.07–1.63)	4.02%
**Mangano 2003** (d = 24 km, Turkey Point NPP, age = 0–9, United States)	0.88 (0.71–1.08)	4.02%
**Boice 2005** (St. Lucie NPP, United States)	0.99 (0.97–1.01)	4.72%
**Total (95% CI)**	1.02 (0.91-1.14)	100%

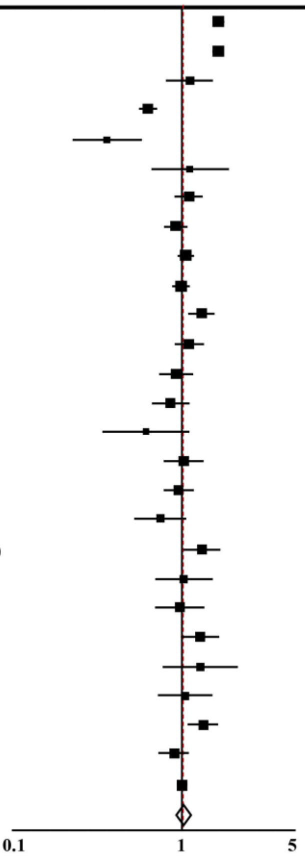

Heterogeneity: Tau^2= 0.06; Chi^2= 418.69; *df* = 26 (*p* < 0.001); I^2= 93.8%
Test for overall effect: z = 0.34 (*p* = 0.736)

Figure S4. Forest plots of studies on the relative risk of all-cancer mortality among residents living within 30 km of nuclear power plants compared with those living farther than 30 km from nuclear power plants. d, average residential distance from nuclear power plants (km); age, average age of population; NPP, nuclear power plant; km, kilometer; RR, relative risk; CI, confidence interval; *df*, degrees of freedom; I^2, I-squared; z, Z-score; *p*, P-value.

Appendix 5 Statistical analysis of subgroup analyses

Table S4. Pooled relative risk of subgroup analyses for all-cancer among workers in nuclear power plants. compared with population without or with the lowest occupational exposure to radiation from nuclear power plants.

Characteristics	Number of study groups	Pooled RR (95% CI)	P-value*	I^2 (95% CI)	P-value†
**Overall**	20	0.85 (0.75–0.97)	0.013	58.9% (0.0%–81.9%)	<0.001
**Geographic area**					
America	5	0.97 (0.81–1.16)	0.714	62.1% (0.0%–88.0%)	0.032
Asia	4	0.75 (0.55–1.03)	0.076	0.0% (0.0%–66.4%)	0.412
Europe	11	0.79 (0.69–0.88)	<0.001	2.4% (0.0%–52.4%)	0.419
**Average cumulative dose of radiation exposure (mSv)**					
0 ≤ dose <25	6	0.96 (0.80–1.14)	0.625	8.7% (0.0%–64.5%)	0.361
25 ≤ dose <50	7	0.71 (0.62–0.80)	<0.001	0.0% (0.0%–37.7%)	0.719
50 ≤ dose <75	2	1.03 (1.00–1.06)	0.040	0.0% (0.0%–0.0%)	0.813
75 ≤ dose <100	2	0.86 (0.56–1.31)	0.477	0.0% (0.0%–75.0%)	0.373
dose ≥100	3	0.95 (0.64–1.43)	0.814	33.0% (0.0%–81.9%)	0.225
**Average annual cumulative dose of radiation exposure (mSv/year)**					
0 ≤ dose rate <5	13	0.83 (0.71–0.97)	0.019	71.1% (0.0%–88.9%)	<0.001
5 ≤ dose rate <10	3	0.79 (0.55–1.15)	0.221	0.0% (0.0%–55.5%)	0.667
dose rate ≥10	4	1.01 (0.76–1.36)	0.930	0.0% (0.0%–57.0%)	0.528
**Comparator indicators**					
National population	14	0.87 (0.75–1.00)	0.052	56.9% (0.0%–82.0%)	0.004
Workers without or with the lowest occupational exposure to radiation	6	0.81 (0.63–1.03)	0.082	32.8% (0.0%–74.3%)	0.190
**Years of follow-up**					
<20 years	7	0.68 (0.58–0.80)	<0.001	0.0% (0.0%–35.7%)	0.758
≥20 years	13	0.93 (0.83–1.05)	0.225	37.2% (0.0%–70.9%)	0.086
**Average age at the end of follow-up**					
<45 years	14	0.83 (0.72–0.95)	0.0063	8.9% (0.0%–52.2%)	0.355
≥45 years	6	0.87 (0.71–1.07)	0.177	76.3% (0.0%–92.5%)	0.001
**The categorization of the risk of bias**					
Tier 1	13	0.81 (0.70–0.93)	0.003	27.1% (0.0%–63.3%)	0.171
Tier 2	7	0.95 (0.83–1.09)	0.482	26.0% (0.0%–70.4%)	0.230

* P-value show effect of characteristics. † P-value of heterogeneity. mSv, millisievert; mSv/year, millisievert per year; NPP, nuclear power plant; km, kilometer; RR, relative risk; CI, confidence interval; I^2, I-squared; NA, not applicable

Table S5. Pooled relative risk of subgroup analyses for all-cancer among residents living within 30 km of the nuclear power plants compared with those living farther than 30 km from nuclear power plants.

Characteristics	Number of study groups	Pooled RR (95% CI)	P-value*	I^2 (95% CI)	P-value†
**Overall**	53	1.05 (1.00–1.09)	0.045	97.2% (75.6%–99.0%)	<0.001
**Geographic area**					
America	18	1.07 (1.00–1.13)	0.046	98.2% (46.1%–99.5%)	<0.001
Asia	6	1.13 (1.05–1.22)	0.001	27.5% (0.0%–71.9%)	0.228
Europe	29	1.02 (0.93–1.10)	0.727	96.4% (87.9%–98.3%)	<0.001
**Average age of study population**					
0 ≤ age <5	20	1.09 (1.00–1.17)	0.0498	88.5% (66.9%–94.3%)	<0.001
5 ≤ age <10	7	0.95 (0.88–1.04)	0.256	61.2% (0.0%–87.2%)	0.017
age ≥10	6	1.03 (0.96–1.10)	0.321	63.8% (0.0%–87.5%)	0.017
Not specified	20	1.93 (0.97–1.10)	0.397	98.7% (73.4%–99.6%)	<0.001
**Sex**					
Females	6	1.06 (0.98–1.15)	0.177	72.2% (0.0%–91.9%)	0.003
Males	6	1.01 (0.95–1.07)	0.809	52.2% (0.0%–83.7%)	0.063
Not specified	41	1.04 (0.99–1.09)	0.127	97.7% (71.9%–99.3%)	<0.001
**Study types**					
Case-control study	2	1.20 (1.01–1.41)	0.035	32.3% (0.0%–86.5%)	0.224
Cohort study	27	1.01 (0.96–1.08)	0.657	92.8% (63.3%–97.0%)	<0.001
Ecologic study	24	1.06 (0.99–1.13)	0.082	98.4% (46.4%–99.6%)	<0.001
**Exposure indicators**					
Community/district	17	1.04 (0.92–1.18)	0.556	97.8% (81.4%–99.2%)	<0.001
Distance	36	1.05 (1.00–1.09)	0.032	96.5% (40.6%–98.9%)	<0.001
**Average distance from NPP (km)***					
0 ≤ distance <10	18	1.07 (0.93–1.23)	0.340	95.9% (81.2%–98.3%)	<0.001
10 ≤ distance <20	13	1.03 (0.96–1.11)	0.364	98.4% (22.6%–99.6%)	<0.001
20 ≤ distance <30	15	1.08 (1.02–1.13)	0.007	79.4% (0.0%–91.5%)	<0.001
Not specified	7	0.94 (0.79–1.12)	0.504	97.0% (34.0%–99.1%)	<0.001
**The categorization of the risk of bias**					
Tier 1	15	1.03 (0.98–1.07)	0.245	37.8% (0.0%–68.6%)	0.069
Tier 2	38	1.05 (1.00–1.10)	0.076	97.9% (77.3%–99.3%)	<0.001

***** P-value show effect of characteristics. † P-value of heterogeneity. mSv, millisievert; mSv/year, millisievert per year; NPP, nuclear power plant; km, kilometer; RR, relative risk; CI, confidence interval; I^2, I-squared; NA, not applicable

Appendix 6 Summarization of basic characteristics of included studies

Table S6. Basic characteristics of the studies for workers in nuclear power plants included in meta-analysis.

Number	Author	Year	Study type	Country (Region/ NPP)	Study period	Main exposure indicator	Definition of research and reference population	Outcome indicator	Outcome diseases we collected
**1**	**Jablon et al.[4]**	1993	Cohort study	Calvert Cliffs NPP, Calvert Count, the United States	1969–1988	Cumulative dose	**Research population:** male workers in the Calvert Cliffs NPP, the United States<br><br>**Reference population:** national population in the United States	**Mortality:** SMR	All-cancer<br>Lung cancer<br>Leukemia<br>Circulatory disease<br>Digestive disease<br>Respiratory disease
**2**	**Auvinen et al.[5]**	2002	Cohort study	Loviisa and Olkiluoto NPPs, Finland	1967–1998	Cumulative dose	**Research population:** workers in the Loviisa and Olkiluoto NPPs, Finland<br><br>**Reference population:** general population in Finland	**Incidence:** SIR	All-cancer
**3**	**Zablotska et al.[6]**	2004	Cohort study	Five NPPs (Point Lepreau, Hydro-Québec, Pickering, Darlington and Brucethe NPPs), Canada	1957–1994	Cumulative dose	**Research population:** workers in the Canadian National Dose Registry<br><br>**Reference population:** Canadian population during 1957–1994 for external comparisons and the lowest dose group (<1.0 mSv) for internal comparison	**Mortality**: RR, SMR, ERR	All-cancer<br>Esophageal cancer<br>Colon cancer<br>Brain and CNS cancer<br>Leukemia<br>Lung cancer<br>Pancreatic cancer<br>Prostate cancer<br>Rectal cancer
**4**	**Kindler et al.[7]**	2006	Case-control study	Greifswald NPP, Greifswald, Germany	1980–2004	Cumulative dose	**Research population:** workers based on the Study of Health in Pomerania in the Greifswald NPP, Germany<br><br>**Reference population:** workers based on the Study of Health in Pomerania who did not expose to radiation in Germany	**Incidence:** OR	Thyroid nodules

Number	Author	Year	Study type	Country (Region/ NPP)	Study period	Main exposure indicator	Definition of research and reference population	Outcome indicator	Outcome diseases we collected
**5**	**Vrijheid et al.[8]**	2007	Cohort study	Australia,<br>Belgium,<br>Canada<br>Finland,<br>France,<br>Hungary,<br>Japan,<br>South Korea,<br>Lithuania,<br>The Slovak Republic,<br>Spain,<br>Sweden,<br>Switzerland,<br>The United Kingdom<br>The United States	1972–1998<br>1969–1994<br>1956–1994<br>1971–1997<br>1968–1994<br>1985–1998<br>1986–1992<br>1992–1997<br>1984–2000<br>1973–1993<br>1970–1996<br>1954–1996<br>1969–1995<br>1955–1992<br>1943–1997	Cumulative dose	**Research population:** workers in nuclear installations in 15 countries<br><br>**Reference population:** national population in each 15 countries	**Mortality:** RR, SMR	All-cancer
**6**	**Cardis et al.[9]**	2007	Cohort study	Australia,<br>Belgium,<br>Canada<br>Finland,<br>France,<br>Hungary,<br>Japan,<br>South Korea,<br>Lithuania,<br>The Slovak Republic,<br>Spain,<br>Sweden,<br>Switzerland,<br>The United Kingdom<br>The United States	1972–1998<br>1969–1994<br>1956–1994<br>1971–1997<br>1968–1994<br>1985–1998<br>1986–1992<br>1992–1997<br>1984–2000<br>1973–1993<br>1970–1996<br>1954–1996<br>1969–1995<br>1955–1992<br>1943–1998	Cumulative dose	**Research population:** workers in nuclear installations in 15 countries<br><br>**Reference population:** national population in each 15 countries	**Mortality**: RR, ERR	Lung cancer
**7**	**Jeong et al.[10]**	2010	Cohort study	South Korea	1992–2005	Cumulative dose	**Research population:** nuclear power workers, who were issued with a dosimeter at nuclear power facilities at any time during 1978–2005 in South Korea<br><br>**Reference population:** workers who did not expose to radiation in nuclear power facilities in South Korea	**Incidence:** RR, SIR	All-cancer<br>Leukemia<br>Liver cancer<br>Lung cancer<br>Stomach cancer<br>Thyroid cancer

Number	Author	Year	Study type	Country (Region/ NPP)	Study period	Main exposure indicator	Definition of research and reference population	Outcome indicator	Outcome diseases we collected
**8**	**Laurent et al.[11]**	2010	Cohort study	France	1961–2003	Cumulative dose	**Research population:** workers of the French National Electricity Company<br><br>**Reference population:** national population in France	**Mortality**: RR	All-cancer<br>Brain and CNS cancer<br>Colon cancer<br>Leukemia<br>Lung cancer<br>Pancreatic cancer<br>Prostate cancer<br>Stomach cancer<br>Rectal cancer<br>Cerebrovascular disease<br>Circulatory disease<br>Digestive disease<br>Respiratory disease
**9**	**Merzenich et al.[12]**	2014	Cohort study	Germany	1991–2008	Cumulative dose	**Research population:** workers in 17 NPPs in west Germany<br><br>**Reference population:** general population in west Germany	**Mortality:** SMR, HR	All-cancer<br>Brain and CNS cancer<br>Colon cancer<br>Leukemia<br>Lung cancer<br>Mesothelioma<br>Pancreatic cancer<br>Prostate cancer<br>Stomach cancer
**10**	**Azizova et al.[13]**	2018	Cohort study	Mayak nuclear installation and Sellafield NPPs, the United Kingdom	1947–2005	Cumulative dose	**Research population:** workers in the Mayak nuclear installation and the Sellafield NPP, the United Kingdom<br><br>**Reference population:** workers who exposed dose less than 5 mSv in the Mayak nuclear installation and the Sellafield NPP, the United Kingdom	**Mortality:** RR, ERR	Cerebrovascular disease<br>Circulatory disease
**11**	**Boice et al.[14]**	2022	Cohort study	The United States	1957–2011	Cumulative dose	**Research population:** workers in 52 NPPs in the United States<br><br>**Reference population:** general population in the United States	**Mortality:** SMR, HR, ERR	All-cancer<br>Brain and CNS cancer<br>Colon cancer<br>Leukemia<br>Lung cancer<br>Pancreatic cancer<br>Prostate cancer<br>Rectal cancer<br>Stomach cancer

Number	Author	Year	Study type	Country (Region/ NPP)	Study period	Main exposure indicator	Definition of research and reference population	Outcome indicator	Outcome diseases we collected
									Cerebrovascular disease<br>Circulatory disease<br>Respiratory disease
**12**	**Kudo et al.[15]**	2022	Cohort study	Japan	2005–2010	Cumulative dose	**Research population:** workers in 17 NPPs in Japan<br><br>**Reference population:** the workers exposed lowest dose (<5.0 mSv)	**Mortality:** RR	Lung cancer
**13**	**Mumma et al.[16]**	2022	Cohort study	The United States	1969–2011	Cumulative dose	**Research population:** workers in 52 NPPs in the United States<br><br>**Reference population:** general population in the United States	**Mortality:** SMR, HR	Mesothelioma

NPP, nuclear power plant; CNS, central nervous system; mSv, millisievert; RR, relative risk; OR, odds ratio; SMR, standard mortality rate; SIR, standard incidence rate; ERR, excess relative risk; HR, hazard ratio.

Table S7. Basic characteristics of the studies for residents living near nuclear power plants included in meta-analysis.

Number	Author	Year	Study type	Country (Region/ NPP)	Study period	Main exposure indicator	Definition of research and reference population	Outcome indicator	Outcome diseases we collected
**1**	**Enstrom et al.[17]**	1983	Ecologic study	San Onofre NPP, California, the United States	1960–1978	Distance	**Research population:** population living near the San Onofre NPP, the United States<br><br>**Reference population:** population living farther than 25 miles from the San Onofre NPP, the United States	**Mortality:** SMR	Leukemia
**2**	**Baron et al.[18]**	1984	Ecologic study	18 nuclear installations (six NPPs: Bradwell, Berkeley, Hinkley, Dungeness, Trawsfynyd, Sizewell NPPs), the United Kingdom	1950–1979	Community/district	**Research population:** population living near the 18 nuclear installations (within a 5-mile radius), the United Kingdom<br><br>**Reference population:** national population in the United Kingdom	**Mortality:** SMR	All-cancer<br>Breast cancer<br>Leukemia<br>Lung cancer<br>Stomach cancer
**3**	**Ewings et al.[19]**	1989	Cohort study	Hinkley NPP, Somerset, the United Kingdom	1959–1986	Distance	**Research population:** population living near the Hinkley NPP, the United Kingdom<br><br>**Reference population:** population living farther than 15.625 km from the Hinkley NPP, the United Kingdom	**Incidence:** SIR	Leukemia
**4**	**Urquhart et al.[20]**	1991	Case-control study	Dounreay NPP, Caithness, the United Kingdom	1968–1986	Distance	**Research population:** population living near the Dounreay NPP, the United Kingdom<br><br>**Reference population:** population living farther than 25 km from the Dounreay NPP, the United Kingdom	**Incidence:** OR	Leukemia

Number	Author	Year	Study type	Country (Region/ NPP)	Study period	Main exposure indicator	Definition of research and reference population	Outcome indicator	Outcome diseases we collected
**5**	**Michaelis et al.[21]**	1992	Cohort study	18 NPPs, west Germany	1980–1990	Distance	**Research population:** population living near the 18 NPPs, west Germany<br><br>**Reference population:** population living in comparison area in west Germany defined in studies	**Incidence:** RR, SIR	All-cancer<br>Acute leukemia
**6**	**Goldsmith et al.[22]**	1992	Cohort study	23 nuclear installations (seven NPPs: Sellafield, Bradwell, Berkeley, Hinkley, Dungeness, Trawsfynyd, Sizewell NPPs), the United Kingdom	1971–1980	Community/district	**Research population:** population living near the seven NPPs, the United Kingdom<br><br>**Reference population:** population living in comparison area in the United Kingdom defined in studies	**Mortality:** SMR<br>**Incidence:** SIR	**Incidence:**<br>All-cancer<br>Leukemia<br>**Mortality:**<br>All-cancer<br>Leukemia
**7**	**McLaughlin et al.[23]**	1993	Ecologic study	Five nuclear installations (two NPPs: Pickering and Bruce NPPs), Canada	1971–1987 (for Pickering NPP)<br>1967–1987 (for Bruce NPP)	Distance	**Research population:** children, aged 0–14 years living in five areas of the province that contain nuclear facilities in Canada<br><br>**Reference population:** children in the province, Canada	**Mortality:** SMR<br>**Incidence:** SIR	**Incidence:** Leukemia<br>**Mortality:** Leukemia
**8**	**Hattchouel et al.[24]**	1995	Cohort study	13 nuclear installations (12 NPPs: Blayais, Bugey, Chinon, Chooz, Cruas, Dampierre, Fessenheim, Gravelines, Marcoule, Paluel, St-Laurent and Tricastin NPPs), France	1968–1989	Distance	**Research population:** population, aged 0–24 years living near 13 nuclear installations, France<br><br>**Reference population:** national population in France	**Mortality:** SMR	Leukemia
**9**	**Morris et al.[25]**	1996	Case-control study	Pilgrim NPP, Massachusetts, the United States	1978–1986	Distance	**Research population:** population living near the Pilgrim NPP, the United States<br><br>**Reference population:** population living farther than 37 km from the	**Incidence:** OR	Leukemia

Number	Author	Year	Study type	Country (Region/ NPP)	Study period	Main exposure indicator	Definition of research and reference population	Outcome indicator	Outcome diseases we collected
							Pilgrim NPP, the United States		
**10**	**López-Abente et al.[26]**	1999	Cohort study	13 nuclear installations (seven NPPs: Zorita, Santa María de Garoña, Vandellós, Almaraz, Ascó, Cofrentes, Trillo NPPs), Spain	1975–1993	Distance	**Research population:** population living near the 13 nuclear installations, Spain<br><br>**Reference population:** population living farther than 50 km from the 13 nuclear installations, Spain	**Mortality:** RR, SMR	Leukemia
**11**	**López-Abente et al.[27]**	2001	Cohort study	12 nuclear installations (four NPPs: Zorita, Santa María de Garoña, Vandellós, Almaraz NPPs), Spain	1975–1993	Distance	**Research population:** population living near the 12 nuclear installations, Spain<br><br>**Reference population:** population living farther than 50 km from the 12 nuclear installations, Spain	**Mortality:** RR, SMR	All-cancer<br>Breast cancer<br>Leukemia<br>Lung cancer<br>Stomach cancer
**12**	**Silva-Mato et al.[28]**	2003	Case-control study	Zorita and Trillo NPPs, Spain	1988–1999	Community/district	**Research population:** population living near the Zorita and Trillo NPPs, Spain<br><br>**Reference population:** population living farther than 30 km from the Zorita and Trillo NPPs, Spain	**Mortality:** OR	All-cancer
**13**	**Mangano et al.[29]**	2003	Ecologic study	14 NPPs, the eastern United States	1988–1997	Distance	**Research population:** population living within 30 miles (48 km) of 14 NPPs, the eastern United States<br><br>**Reference population:** national population in the United States	**Mortality:** Rate<br>**Incidence:** Rate	**Incidence**:<br>All-cancer<br>Leukemia<br>**Mortality**:<br>All-cancer

Number	Author	Year	Study type	Country (Region/ NPP)	Study period	Main exposure indicator	Definition of research and reference population	Outcome indicator	Outcome diseases we collected
**14**	**White-Koning et al.[30]**	2004	Cohort study	29 nuclear installations (19 NPPs: Belleville, Bugey, Cattenom, Chinon, Chooz, Civaux, Cruas, Dampierre, Fessenheim, Flamanville, Golfech, Gravelines, Le Blayais, Nogent, Paluel, Penly, St. Alban, St. Laurent and Tricastin NPPs), France	1990–1998	Distance	**Research population:** population living within 20 km of 29 nuclear installations, included 19 NPPs, France<br><br>**Reference population:** national population in France	**Incidence:** SIR	Leukemia
**15**	**Yoshimoto et al.[31]**	2004	Ecologic study	16 NPPs (Tokai, Fugen, Mihama, Fukushima I, Takahama, Shimane, Genkai, Hamaoka, Ikata, Oi,Fukushima II, Onagawa, Sendai, Kashiwazaki-kariwa, Tomari and Shika NPPs), Japan	1972–1997	Community/district	**Research population:** population living near 16 NPPs, Japan<br><br>**Reference population:** population living in comparison community/district in Japan defined in studies	**Mortality:** Rate, ERR	Leukemia
**16**	**Boice et al.[32]**	2005	Cohort study	St. Lucie NPP, Florida, the United States	1950–2000	Community/district	**Research population:** population living near the St. Lucie NPP, the United States<br><br>**Reference population:** population living in comparison community/district (Polk and Volusia Counties) in the United States	**Mortality:** RR, SMR	All-cancer<br>Leukemia
**17**	**Kaatsch et al.[33]**	2008	Case-control study	16 NPPs, Germany	1980–2003	Distance	**Research population:** population living near the 16 NPPs, Germany<br><br>**Reference population:** population living farther than 70 km from 16 NPPs, Germany	**Incidence:** OR, SIR	Leukemia
**18**	**Zadnik et al.[34]**	2008	Ecologic study	Krško NPP, Krško, Slovenia	1984–2003	Community/district	**Research population:** population living near the Krško NPP, Slovenia<br><br>**Reference population:** population in other 11	**Incidence:** RR	All-cancer<br>Breast cancer<br>Leukemia<br>Lung cancer<br>Stomach cancer<br>Thyroid cancer

Number	Author	Year	Study type	Country (Region/ NPP)	Study period	Main exposure indicator	Definition of research and reference population	Outcome indicator	Outcome diseases we collected
							Slovenian statistical regions		
**19**	**Spix et al.[35]**	2009	Case-control study	16 NPPs, Germany	1993–2003	Community/district	**Research population:** children aged <5 years living near 16 NPPs, Germany<br><br>**Reference population:** population living in comparison community/district in Germany	**Incidence:** OR	Leukemia
**20**	**Mangano et al.[36]**	2009	Cohort study	Seven NPPs (Indian Point, Oyster Creek, Salem/Hope Creek, Limerick, Peach Bottom, Susquehanna, Three Mile Island NPPs), New Jersey, the United States	2001–2005	Distance	**Research population:** population living near seven NPPs, the United States<br><br>**Reference population:** national population in the United States	**Incidence:** Rate	Thyroid cancer
**21**	**Heinävaara et al.[37]**	2010	Cohort study<br>Case-control study<br>Ecologic study	Loviisa and Olkiluoto NPPs, Finland	1975–2004<br>1981–2000<br>1977–2004	Distance	**Research population:** population living near the Loviisa and Olkiluoto NPPs, Finland<br><br>**Reference population:** population living farther than 15-50 km from the Loviisa and Olkiluoto NPPs, Finland	**Incidence:** RR, SIR, OR	All-cancer<br>Leukemia
**22**	**Spycher et al.[38]**	2011	Cohort study	Five NPPs (BeznauI, II, Leibstadt, Mühleberg, Gösgen NPPs), Switzerland	1985–2009	Distance	**Research population:** children, aged 0–15 living near five NPPs, Switzerland<br><br>**Reference population:** children, aged 0–15 living farther than 15 km from five NPPs, Switzerland	**Incidence:** Rate, RR	All-cancer<br>Leukemia

Number	Author	Year	Study type	Country (Region/ NPP)	Study period	Main exposure indicator	Definition of research and reference population	Outcome indicator	Outcome diseases we collected
23	**Ma et al.[39]**	2011	Cohort study	Seven NPPs (Braidwood, Byron, Clinton, Dresden, LaSalle, Quad Cities, Zion NPPs), Illinois, the United States	1986–2005	Distance	**Research population:** children, aged 0–14 living near seven NPPs, the United States<br><br>**Reference population:** population in Illinois, the United States	**Incidence:** RR, SIR	Leukemia
24	**Bazyka et al.[40]**	2012	Cohort study	Khmelnytskyi and Zaporizhzhia NPPs, Ukraine	2003–2008	Community/district	**Research population:** population living near the Khmelnytskyi and Zaporizhzhia NPPs, Ukraine<br><br>**Reference population:** population living in regions without cities with nuclear facilities, Ukraine	**Incidence:** SIR	All-cancer<br>Breast cancer<br>Leukemia<br>Lung cancer<br>Thyroid cancer
25	**Ahn et al.[41]**	2012	Cohort study	Four NPPs (Kori, Wolseong, Hanbit and Hanul NPPs), South Korea	1992–2010	Distance	**Research population:** population living near four NPPs, South Korea<br><br>**Reference population:** population living farther than 30 km from four NPPs, South Korea	**Incidence:** CR, ASR, HR	All-cancer<br>Breast cancer<br>Leukemia<br>Lung cancer<br>Stomach cancer<br>Thyroid cancer
26	**Sermage-Faure et al.[42]**	2012	Cohort study	19 NPPs, France	2002–2007	Distance	**Research population:** population living near 19 NPPs, France<br><br>**Reference population:** population living farther than 20 km from 19 NPPs, France	**Incidence:** SIR	Acute leukemia
27	**Bithell et al.[43]**	2013	Case-control study	13 NPPs (Bradwell, Berkeley, Chapelcross, Hinkley, Hartlepool, Heysham, Hunterston, Dungeness, Oldbury, Torness, Trawsfynyd, Sizewell and Wylfa NPPs), the United Kingdom	1962–2007	Distance	**Research population:** population living near 13 NPPs, the United Kingdom<br><br>**Reference population:** population living farther than 25 km from 13 NPPs, the United Kingdom	**Incidence:** OR	Leukemia

Number	Author	Year	Study type	Country (Region/ NPP)	Study period	Main exposure indicator	Definition of research and reference population	Outcome indicator	Outcome diseases we collected
**28**	**Lane et al.[44]**	2013	Ecologic study	Three NPPs (Pickering, Bruce and Darlington NPPs), Ontario, Canada	1990–2008	Distance	**Research population:** population living near three NPPs, Canada<br><br>**Reference population:** general population in Ontario, Canada	**Incidence:** SIR	All-cancer<br>Breast cancer<br>Leukemia<br>Lung cancer<br>Stomach cancer<br>Thyroid cancer
**29**	**Bollaerts et al.[45]**	2014	Ecologic study	Five nuclear installations (three NPPs: Doel, Tihange, Chooz NPPs), Belgium	2000–2008, 2004–2008	Distance	**Research population:** population living near five nuclear installations, Belgium<br><br>**Reference population:** national population in Belgium	**Incidence:** RR, SIR	Thyroid cancer
**30**	**Wang et al.[46]**	2016	Cohort study	Three NPPs (Jinshan, Kuosheng, Maanshan NPPs), Taiwan	1979–2003	Community/district	**Research population:** population living near three NPPs, Taiwan<br><br>**Reference population:** population living farther than 22.76 km from three NPPs, Taiwan	**Incidence:** RR	All-cancer<br>Breast cancer<br>Leukemia<br>Lung cancer<br>Stomach cancer<br>Thyroid cancer
**31**	**Khomenko et al.[47]**	2017	Ecologic study	Zaporizhzhia NPP, Ukraine	2003–2015	Community/district	**Research population:** population living near the Zaporizhzhia NPP, Dnipropetrovsk region, Ukraine<br><br>**Reference population:** general population living in Petrivsk district, Ukraine	**Incidence:** Rate	Digestive disease
**32**	**Demoury et al.[48]**	2017	Ecologic study	Four nuclear installations (two NPPs: Doel and Tihange NPPs), Belgium	2000–2014 (the Flemish Region) 2004–2014 (the Brussels-Capital Region and the Walloon Region)	Distance	**Research population:** population living near four nuclear installations, Belgium<br><br>**Reference population:** national population in Belgium	**Incidence:** RR	Thyroid cancer

Number	Author	Year	Study type	Country (Region/ NPP)	Study period	Main exposure indicator	Definition of research and reference population	Outcome indicator	Outcome diseases we collected
**33**	**Desbiolles et al.[49]**	2018	Ecologic study	Seven NPPs (Blayais, Bugey, Civaux, Fessenheim, Flamanville, Saint-Alban and Chooz NPPs), France	1995–2011	Distance	**Research population:** population living near seven NPPs, France<br><br>**Reference population:** national population in France	**Incidence:** RR	Thyroid cancer
**34**	**Demoury et al.[50]**	2021	Ecologic study	Four nuclear installations (two NPPs: Doel and Tihange NPPs), Belgium	2006–2016	Distance	**Research population:** population four nuclear installations, Belgium<br><br>**Reference population:** national population in Belgium	**Incidence:** RR	Leukemia

NPP, nuclear power plant; RR, relative risk; OR, odds ratio; SMR, standard mortality rate; SIR, standard incidence rate; ERR, excess relative risk; CR, crude ratio; ASR, age standardized ratio; HR, hazard ratio.

Appendix 7 Risk of bias

We used the National Toxicology Program Office of Health Assessment and Translation (NTP/OHAT) risk of bias tool to assess the risk of bias [51]. For workers, no adjustments were performed for confounders, such as lifestyle, occupational exposure to radiation, and socioeconomic status, in four studies; therefore confounding bias was rated as “probably high” for those studies [4, 8, 9, 14]. For exposure and outcome detection bias, most of the studies were rated as “definitely low” and “probably low”. In summary, regarding workers, nine studies were categorized as tier 1 and four as tier 2. For residents, no adjustments were performed for confounders, such as lifestyle and socioeconomic status, in most studies, or adjustment was performed for only one or two demographic factors; confounding bias was rated as “probably high” for those studies. For exposure and outcome detection bias, data from trustworthy databases were used in all of the studies; hence they were all rated as “definitely low” and “probably low”. The conflict of interest in one study resulted in a rating of “definitely high” for the risk of bias [43]. For other studies that did not provide conflict of interest information, we rated “probably high”. In summary, regarding residents, 12 studies were categorized as tier 1 and 22 as tier 2.

We downgraded confidence owing to the risk of bias across studies based on the guideline in the NTP/OHAT risk of bias tool, as follows [51]:

a. Not likely: Most information is derived from tier 1 studies (low risk of bias for all key domains).

b. Serious: Most information is derived from tier 1 and 2 studies.

c. Very serious: The proportion of information from tier 3 studies (at high risk of bias for all key domains) is sufficient to affect the interpretation of results.

Table S8. Risk of bias of individual studies of workers in nuclear power plants according the NTP/OHAT risk of bias tool.

(1) Jablon et al. 1993[4]

Domains	Rating	Comments
**1. Selection bias**		
1.1 Did selection of study participants result in appropriate comparison groups?	Definitely low	The study included the 8,954 male workers in Calvert Cliffs nuclear power plant in the United States (US), who were employed at any time up to the end of 1984. The information of expected risk was based on national death rates. The subject came from the same cohort.
**2. Confounding bias**		
2.1 Did the study design or analysis account for important confounding and modifying variables?	Probably high	The authors adjusted for age. They did not adjust for other potential confounding factor.
**3. Attrition/Exclusion bias**		
3.1 Were outcome data complete without attrition or exclusion from analysis?	Probably high	Not specified
**4. Detection bias**		
4.1 Can we be confident in the exposure characterization?	Definitely low	These measurements of dose were from film badges or other dosimeters.
4.2 Can we be confident in the outcome assessment?	Probably low	The vital status was determined from the Social Security Administration records and the National Death Index.
**5. Selective reporting bias**		
5.1 Were all measured outcomes reported?	Definitely low	The outcome has been reported in SMR and 95% CI. The outcome provided sufficient data, which could be included in meta-analysis.
**6. Other bias**		
6.1 Conflict of interest	Probably high	Not specified Data was collected from the staff of the Calvert Cliffs nuclear power plant which made records available for the study.

(2) Auvinen et al. 2002[5]

Domains	Rating	Comments
**1. Selection bias**		
1.1 Did selection of study participants result in appropriate comparison groups?	Definitely low	The study included the 15,619 workers in two nuclear power plants in Finland, who had been monitored for radiation exposure for at least six months.<br>The reference population was defined as general population in Finland.<br>The subject was came from the same cohort.
**2. Confounding bias**		
2.1 Did the study design or analysis account for important confounding and modifying variables?	Probably low	The authors adjusted for sex, educational level, type of employment, and smoking and alcohol consumption.<br>They did not adjust for other potential confounding factor.
**3. Attrition/Exclusion bias**		
3.1 Were outcome data complete without attrition or exclusion from analysis?	Definitely low	Total 119 of 15,619 subjects had emigrated during the follow-up and their follow-up ended at emigration.
**4. Detection bias**		
4.1 Can we be confident in the exposure characterization?	Definitely low	Both of the power companies used their own dosimetry service and reported the results to the National Registry of Occupational Radiation Exposure.
4.2 Can we be confident in the outcome assessment?	Probably low	The data of death were classified by the Finnish Cancer Registry.
**5. Selective reporting bias**		
5.1 Were all measured outcomes reported?	Definitely low	The outcome has been reported in SIR and 95% CI.<br>The outcome provided sufficient data, which could be included in meta-analysis.
**6. Other bias**		
6.1 Conflict of interest	Probably high	Not specified<br>The study was supported in part by grants from the Duodecim Society and the Finnish Cancer Society.

(3) Zablotska et al. 2004[6]

Domains	Rating	Comments
**1. Selection bias**		
1.1 Did selection of study participants result in appropriate comparison groups?	Definitely low	The study included 45,656 workers in the Canadian National Dose Registry (NDR), had to be monitored in a nuclear facility in Canada for one year.<br>The reference population was defined as Canadian population during 1957-1994 for external comparisons and the lowest dose group (<1.0 mSv) for internal comparisons.
**2. Confounding bias**		
2.1 Did the study design or analysis account for important confounding and modifying variables?	Definitely low	The authors adjusted for age, sex, calendar year, facility types, socioeconomic status, and duration of monitoring.
**3. Attrition/Exclusion bias**		
3.1 Were outcome data complete without attrition or exclusion from analysis?	Definitely low	Total 188 out of 45,656 workers were excluded because of irregularities in their records, leaving a cohort for analysis of 45,468.
**4. Detection bias**		
4.1 Can we be confident in the exposure characterization?	Definitely low	Most Canadian companies in the NDR reported thresholds were generally 0.2 mSv, and doses below this level were recorded as zero dose, but the exception was the Atomic Energy of Canada Limited, where from 1972 to 1994 the threshold was set at 0.05 mSv. No attempts were made to correct for the potential underestimation of dose where the doses were recorded as zero. However, the authors thought that the latest report from the International Agency for Research on Cancer suggests that the impact of measurement errors in dosimetry is likely to be very small.
4.2 Can we be confident in the outcome assessment?	Definitely low	The data of outcome was classified based on the 9th version of the International Classification of Diseases (ICD).
**5. Selective reporting bias**		
5.1 Were all measured outcomes reported?	Definitely low	The outcome has been reported in RR, SMR, ERR and 95% CI.<br>The outcome provided sufficient data, which could be included in meta-analysis.
**6. Other bias**		
6.1 Conflict of interest	Probably high	Not specified<br>The study was funded for record linkage and other resources by the Health Canada.

(4) Kindler et al. 2006[7]

Domains	Rating	Comments
**1. Selection bias**		
1.1 Did selection of study participants result in appropriate comparison groups?	Definitely low	The study included 71 workers based on the Study of Health in Pomerania (SHIP), in nuclear power plants, Energiewerke Nord, who were 38 to 57 years of age.<br>The reference population were also based on the SHIP and was defined as 670 males aged between 38 and 57 years without occupational exposure to ionizing radiation.
**2. Confounding bias**		
2.1 Did the study design or analysis account for important confounding and modifying variables?	Definitely low	The authors adjusted for age, educational level, smoking status, and medical histories on thyroid disorders.
**3. Attrition/Exclusion bias**		
3.1 Were outcome data complete without attrition or exclusion from analysis?	Probably high	Not specified
**4. Detection bias**		
4.1 Can we be confident in the exposure characterization?	Probably low	The cumulative lifetime radiation dose of each employee was obtained from individual dosimetry records provided by the radiation protection department of the Energiewerke Nord.
4.2 Can we be confident in the outcome assessment?	Probably low	The outcomes were performed thyroid ultrasonography using an ultrasound VST-gateway with a linear array transducer. All examinations were carried out by the same observers. Thyroid volume was calculated as length width depth 0.479 milliliter for each lobe.
**5. Selective reporting bias**		
5.1 Were all measured outcomes reported?	Definitely low	The outcome has been reported in OR and 95% CI.<br>The outcome provided sufficient data, which could be included in meta-analysis.
**6. Other bias**		
6.1 Conflict of interest	Probably high	Not specified<br>The study was funded by the Federal Ministry of Education and Research (grant no. ZZ9603), the Ministry of Cultural Affairs as well as the Social Ministry of the Federal State of Mecklenburg West Pomerania.

(5) Vrijheid et al. 2007[8]

Domains	Rating	Comments
**1. Selection bias**		
1.1 Did selection of study participants result in appropriate comparison groups?	Definitely low	The study included cohorts of workers in the nuclear industry in 15 countries (Australia, Belgium, Canada, Finland, France, Hungary, Japan, Korea, Lithuania, the Slovak Republic, Spain, Sweden, Switzerland, the United Kingdom [UK] and the US).
**2. Confounding bias**		
2.1 Did the study design or analysis account for important confounding and modifying variables?	Probably high	The authors adjusted for age, sex, smoking status, and socioeconomic status.<br>They did not adjust for other potential confounding factor.
**3. Attrition/Exclusion bias**		
3.1 Were outcome data complete without attrition or exclusion from analysis?	Definitely low	For each cohort, if workers were known to have been involved in a radiation incident or accident or have exceptionally high annual doses would be excluded from analyses.
**4. Detection bias**		
4.1 Can we be confident in the exposure characterization?	Probably low	Dosimetry information was collected from records of individual facilities and/or national dose registries.
4.2 Can we be confident in the outcome assessment?	Definitely low	Causes of death were therefore provided in the 6th, 7th, 8th, 9th, and 10th versions of the ICD, depending on year and country.
**5. Selective reporting bias**		
5.1 Were all measured outcomes reported?	Definitely low	The outcome has been reported in RR, SMR and 95% CI.<br>The outcome provided sufficient data, which could be included in meta-analysis.
**6. Other bias**		
6.1 Conflict of interest	Definitely low	No other authors have conflicts of interest to declare.<br>Financial support for coordination of the International Study was provided by the European Union (contracts F13P-CT930066, F14P CT96-0062, FIGH-CT1999-20001), the US. Centers for Disease Control and Prevention (Co-operative agreement U50/CCU011778), and the Canadian Nuclear Safety Commission.

(6) Cardis et al. 2007[9]

Domains	Rating	Comments
**1. Selection bias**		
1.1 Did selection of study participants result in appropriate comparison groups?	Definitely low	The study included cohorts of workers in the nuclear industry in 15 countries (Australia, Belgium, Canada, Finland, France, Hungary, Japan, Korea, Lithuania, the Slovak Republic, Spain, Sweden, Switzerland, the UK and the US).
**2. Confounding bias**		
2.1 Did the study design or analysis account for important confounding and modifying variables?	Probably high	The authors adjusted for smoking status, and socioeconomic status.<br>They did not adjust for other potential confounding factor.
**3. Attrition/Exclusion bias**		
3.1 Were outcome data complete without attrition or exclusion from analysis?	Definitely low	For each cohort, if workers were known to have been involved in a radiation incident or accident or have exceptionally high annual doses would be excluded from analyses.
**4. Detection bias**		
4.1 Can we be confident in the exposure characterization?	Probably low	Dosimetry information was collected from records of individual facilities and/or national dose registries.
4.2 Can we be confident in the outcome assessment?	Probably low	The information of vital status and date and cause of death for workers who had died were collected by each cohort member at the national level.
**5. Selective reporting bias**		
5.1 Were all measured outcomes reported?	Definitely low	The outcome has been reported in RR, ERR and 90% CI.<br>The outcome provided sufficient data, which could be included in meta-analysis.
**6. Other bias**		
6.1 Conflict of interest	Definitely low	No other authors have conflicts of interest to declare.<br>Financial support for coordination of the International Study was provided by the European Union (contracts F13PCT930066, F14P-CT96–0062, FIGH-CT1999–20001) and the Canadian Nuclear Safety Commission.

(7) Jeong et al. 2010[10]

Domains:	Rating	Comments
**1. Selection bias**		
1.1 Did selection of study participants result in appropriate comparison groups?	Definitely low	The study included 8,429 nuclear power workers in Korea, who were issued with a dosimeter at nuclear power facilities at any time during 1978–2005 and were called "radiation workers".<br>In addition, 7,807 non-radiation workers in nuclear power facilities in Korea were also included in the study as comparison group.
**2. Confounding bias**		
2.1 Did the study design or analysis account for important confounding and modifying variables?	Definitely low	The authors adjusted for age, birth year, monitoring status and smoking status.
**3. Attrition/Exclusion bias**		
3.1 Were outcome data complete without attrition or exclusion from analysis?	Definitely low	Females were excluded from the present study to focus on male subjects leaving a cohort for analysis of 16,236 male workers.
**4. Detection bias**		
4.1 Can we be confident in the exposure characterization?	Definitely low	The study used the radiation exposure database of the Korea Hydro & Nuclear Power Co., Ltd. because it provided whole dose records from 1978, and more detailed information such as radiation type (photon or neutron) and exposure type (internal or external) for each record.
4.2 Can we be confident in the outcome assessment?	Definitely low	The cancer incidence status was evaluated for the period of January 1, 1992 to December 31, 2005, the sites of cancer incidence were based on the 10th version of the ICD codes, and the date of cancer diagnosis was extracted from the Korean Central Cancer Registry.
**5. Selective reporting bias**		
5.1 Were all measured outcomes reported?	Definitely low	The outcome has been reported in RR, SIR and 95% CI.<br>The outcome provided sufficient data, which could be included in meta-analysis.
**6. Other bias**		
6.1 Conflict of interest	Probably high	Not specified<br>This study was supported by a grant of the Korean Ministry of Education, Science and Technology (KOSEF 2009-0062385) and partially by a grant of the Korea Hydro & Nuclear Power Co., Ltd. (E07NS55).

(8) Laurent et al. 2010[11]

Domains	Rating	Comments
**1. Selection bias**		
1.1 Did selection of study participants result in appropriate comparison groups?	Definitely low	The study included workers of the French National Electricity Company (EDF), who had been monitored for exposure to ionizing radiation between 1961 and 1994.
**2. Confounding bias**		
2.1 Did the study design or analysis account for important confounding and modifying variables?	Definitely low	The authors adjusted for age, sex and calendar period, educational level, and smoking status.
**3. Attrition/Exclusion bias**		
3.1 Were outcome data complete without attrition or exclusion from analysis?	Definitely low	Workers flagged as substantially exposed to neutrons were excluded from the main analysis.
**4. Detection bias**		
4.1 Can we be confident in the exposure characterization?	Definitely low	The measurement of external exposure to high-energy photon radiation (gamma rays, energy range 100–3,000 kiloelectron-volt) was used personal Kodak type two film badge dosimeters, which were worn on the chest. dosimeter calibration systems in use at EDF changed over time.
4.2 Can we be confident in the outcome assessment?	Definitely low	The causes of death coded according to the 8th version of the ICD for period of 1968–1978, the 9th version of the ICD for period of 1979–1999 and the 10th version of the ICD for period of 2000–2003 were obtained from the National Causes of Death Registry.
**5. Selective reporting bias**		
5.1 Were all measured outcomes reported?	Definitely low	The outcome has been reported in RR, 90% CI and 95% CI. The outcome provided sufficient data, which could be included in meta-analysis.
**6. Other bias**		
6.1 Conflict of interest	Probably high	Not specified<br>The study was funded by the Électricité de France.

(9) Merzenich et al. 2014[12]

Domains	Rating	Comments
**1. Selection bias**		
1.1 Did selection of study participants result in appropriate comparison groups?	Definitely low	The study included workers in 17 nuclear power plants in West Germany, who were eligible for four inclusion criteria. The reference population was defined as the general population of West Germany.
**2. Confounding bias**		
2.1 Did the study design or analysis account for important confounding and modifying variables?	Probably low	The authors adjusted for age and duration of employment, and smoking and alcohol consumption.<br>They did not adjust for other potential confounding factor.
**3. Attrition/Exclusion bias**		
3.1 Were outcome data complete without attrition or exclusion from analysis?	Definitely low	For workers lost to follow-up, the end of follow-up was defined as the date of death or last information date.<br>Seventy workers (0.7% of all workers included) were lost to follow-up.
**4. Detection bias**		
4.1 Can we be confident in the exposure characterization?	Definitely low	The data of individual dosimeters was monitored by officially approved film badges from external high-energy photon radiation.
4.2 Can we be confident in the outcome assessment?	Definitely low	The causes of death were coded by the Statistical Office of Rhineland Palatinate according to the 9th revision of the ICD for deaths in the period of 1991 to 1997 and according to the 10th revision of the ICD for deaths in the period of 1998 to 2008.
**5. Selective reporting bias**		
5.1 Were all measured outcomes reported?	Definitely low	The outcome has been reported in SMR, HR and 95% CI.<br>The outcome provided sufficient data, which could be included in meta-analysis.
**6. Other bias**		
6.1 Conflict of interest	Probably high	Not specified<br>The study was funded by the Berufsgenossenschaft Energie Textil Elektro Medienerzeugnisse (BGETEM).

(10) Azizova et al. 2018[13]

Domains	Rating	Comments
**1. Selection bias**		
1.1 Did selection of study participants result in appropriate comparison groups?	Definitely low	The study included 22,374 workers for Mayak worker cohort (MWC) and 23,443 workers for Sellafield worker cohort (SWC).
**2. Confounding bias**		
2.1 Did the study design or analysis account for important confounding and modifying variables?	Definitely low	The authors adjusted for sex, attained age, calendar period, migration status, external gamma-ray dose and dose from internal alpha radiation, and smoking and alcohol consumption.
**3. Attrition/Exclusion bias**		
3.1 Were outcome data complete without attrition or exclusion from analysis?	Definitely low	For the MWC, workers with unknown vital status, and for the SWC, workers with unknown vital status and those who had emigrated from the UK were defined as workers lost to follow-up.<br>The proportion of lost to follow-up were 5% and 2% for the MWC and SWC respectively.
**4. Detection bias**		
4.1 Can we be confident in the exposure characterization?	Definitely low	For external gamma rays and internal alpha-radiation doses, the measurement systems for MWC and SWC were clearly defined in study.
4.2 Can we be confident in the outcome assessment?	Definitely low	Causes of death were coded consistently in both cohorts in accordance with the 9th version of the ICD. Mortality risk was investigated for all circulatory disease (ICD-9: 390–459 codes), as well as for ischemic heart disease (ICD-9: 410–414 codes) and cerebrovascular disease (ICD-9: 430–438 codes), separately.
**5. Selective reporting bias**		
5.1 Were all measured outcomes reported?	Definitely low	The outcome has been reported in RR, ERR and 95% CI.<br>The outcome provided sufficient data, which could be included in meta-analysis.
**6. Other bias**		
6.1 Conflict of interest	Probably high	Not specified<br>This study was funded by the European Union Seventh Framework Programme under Grant Agreement No. 249675 "Epidemiological Studies of Exposed Southern Urals Populations".

(11) Boice et al. 2022[14]

Domains	Rating	Comments
**1. Selection bias**		
1.1 Did selection of study participants result in appropriate comparison groups?	Definitely low	The study included 1,065,703 workers employed at nuclear power plants in the United States in 1957 through 2011 was available for study: 1,001,958 workers within the Radiation Exposure Information and Reporting System (REIRS) maintained by the NRC and 63,745 workers within the Landauer, Inc. dosimetry database.
**2. Confounding bias**		
2.1 Did the study design or analysis account for important confounding and modifying variables?	Probably high	The authors adjusted for sex, year of birth, duration of monitoring, and socioeconomic status.<br>They did not adjust for other potential confounding factor.
**3. Attrition/Exclusion bias**		
3.1 Were outcome data complete without attrition or exclusion from analysis?	Definitely low	Workers were excluded if the date of first monitoring was after 1984 (n = 592,935) or if personal identifiers needed for tracing, specifically a Social Security Number (n = 5,738) were absent.
**4. Detection bias**		
4.1 Can we be confident in the exposure characterization?	Definitely low	The data of annual individual dosimeters records were available from the REIRS and Landauer files.
4.2 Can we be confident in the outcome assessment?	Definitely low	The causes of death were coded according to the 8th, 9th and 10th versions of the ICD.
**5. Selective reporting bias**		
5.1 Were all measured outcomes reported?	Definitely low	The outcome has been reported in SMR, HR, ERR and 95% CI.<br>The outcome provided sufficient data, which could be included in meta-analysis.
**6. Other bias**		
6.1 Conflict of interest	Definitely low	No potential conflict of interest was reported by the authors.

(12) Kudo et al. 2022[15]

Domains	Rating	Comments
**1. Selection bias**		
1.1 Did selection of study participants result in appropriate comparison groups?	Definitely low	The study included the workers based on the cohort study of cancer mortality among japanese nuclear workers, who were registered in the Radiation Dose Registry Center (RADREC) within the Radiation Effects Association as of the end of March 1999.
**2. Confounding bias**		
2.1 Did the study design or analysis account for important confounding and modifying variables?	Probably low	The authors adjusted for several variable of lifestyle, socioeconomic status, and there was indirect message to indicates adjusted for confounding factor.
**3. Attrition/Exclusion bias**		
3.1 Were outcome data complete without attrition or exclusion from analysis?	Definitely low	Female workers were excluded from the analysis.<br>Workers who are unable to be identified in the RADREC, no answers written in the questionnaire, and no follow-up period were excluded.
**4. Detection bias**		
4.1 Can we be confident in the exposure characterization?	Definitely low	The data of individual dosimeters records were supplied by the RADREC.
4.2 Can we be confident in the outcome assessment?	Definitely low	The causes of death were coded according to the 10th version of the ICD.
**5. Selective reporting bias**		
5.1 Were all measured outcomes reported?	Definitely low	The outcome has been reported in RR and 95% CI.<br>The outcome provided sufficient data, which could be included in meta-analysis.
**6. Other bias**		
6.1 Conflict of interest	Probably high	Not specified<br>The study was fully funded by the Nuclear Regulation Authority of Japan.

(13) Mumma et al. 2022[16]

Domains	Rating	Comments
**1. Selection bias**		
1.1 Did selection of study participants result in appropriate comparison groups?	Definitely low	The study was formed by combining two occupational cohorts, which were drawn from either the US Nuclear Regulatory Commission and the Radiation Exposure Information and Reporting System database or the electronic records of Landauer, Incorporated.
**2. Confounding bias**		
2.1 Did the study design or analysis account for important confounding and modifying variables?	Probably low	The authors adjusted for sex, year of birth, potential occupational exposure to asbestos, and year of first radiation monitoring.<br>Using indicates measures to derive smoking and socioeconomic status data.
**3. Attrition/Exclusion bias**		
3.1 Were outcome data complete without attrition or exclusion from analysis?	Definitely low	Follow-up within the first 10 years of radiation exposure were excluded in the internal Cox analyses.
**4. Detection bias**		
4.1 Can we be confident in the exposure characterization?	Definitely low	The data of individual dosimeters records were from the US Department of Energy (DOE) Radiation Exposure Monitoring Systems dosimetry database, historical DOE study databases as well as the US military service dosimetry databases.
4.2 Can we be confident in the outcome assessment?	Definitely low	The causes of death were coded according to the 8th, 9th, and 10th versions of the ICD.
**5. Selective reporting bias**		
5.1 Were all measured outcomes reported?	Definitely low	The outcome has been reported in SMR, HR and 95% CI.<br>The outcome provided sufficient data, which could be included in meta-analysis.
**6. Other bias**		
6.1 Conflict of interest	Definitely high	The authors declared that “only two authors (one is first author) have previously consulted with a former manufacturer of products containing asbestos on matters related to litigation.”, and other authors report no conflict of interest.

Table S9. Risk of bias of individual studies of residents living near nuclear power plants according the NTP/OHAT risk of bias tool.

(1) Enstrom et al. 1983[17]

Domains	Rating	Comments
**1. Selection bias**		
1.1 Did selection of study participants result in appropriate comparison groups?	Definitely low	The study included residents living within 25 miles of the San Onofre nuclear and the personal data were collected from the Vital Statistics of California reactor from same period and region.
**2. Confounding bias**		
2.1 Did the study design or analysis account for important confounding and modifying variables?	Probably high	The authors adjusted for age.<br>They did not adjust for other potential confounding factor.
**3. Attrition/Exclusion bias**		
3.1 Were outcome data complete without attrition or exclusion from analysis?	Definitely low	Deaths of unknown age and the Pendleton division, consisting of the Camp Pendleton Marine Corps Reservation, has been excluded because it has a highly transient population of young recruits undergoing advanced training and no leukemia deaths were reported among persons under age 20 with Camp Pendleton as a place of residence were excluded.
**4. Detection bias**		
4.1 Can we be confident in the exposure characterization?	Definitely low	The death tapes were then processed using the Statistical Analysis System (SAS) to obtain deaths by sex, race, five-year age group, year of death, cause of death, and county of residence.
4.2 Can we be confident in the outcome assessment?	Definitely low	The death tapes including each California resident death in the period of 1960 to 1978 were obtained from the Vital Statistics Branch of the California Department of Health Services in Sacramento and the underlying cause of death for each death was assigned by the California Vital Statistics Branch nosologist using the ICD.
**5. Selective reporting bias**		
5.1 Were all measured outcomes reported?	Definitely low	The outcome has been reported in SMR and 95%CI.<br>The outcome provided sufficient data, which could be included in meta-analysis.
**6. Other bias**		
6.1 Conflict of interest	Probably high	Not specified<br>This research has been supported by National Cancer Institute Preventive Oncology Academic Award CA00748 and by Southern California Edison Company.

(2) Baron et al. 1984[18]

Domains	Rating	Comments
**1. Selection bias**		
1.1 Did selection of study participants result in appropriate comparison groups?	Definitely low	The study included the cancer mortality data examined for the small area (within a 5-mile radius) around 14 nuclear and five non-nuclear facilities in England and Wales.<br>Expected numbers of deaths were obtained by multiplying England and Wales death rates for a given year by the estimated age/sex-specific populations of the local authority areas.
**2. Confounding bias**		
2.1 Did the study design or analysis account for important confounding and modifying variables?	Probably high	The authors adjusted for age.<br>They did not adjust for other potential confounding factor.
**3. Attrition/Exclusion bias**		
3.1 Were outcome data complete without attrition or exclusion from analysis?	Probably high	Not specified
**4. Detection bias**		
4.1 Can we be confident in the exposure characterization?	Definitely low	The areas included must covered at least one local authority and all geographic units used were as defined prior to the 1974 local government reorganization (i.e. the Population Statistics Division, and the Office of Population Censuses and Surveys).
4.2 Can we be confident in the outcome assessment?	Definitely low	The cause of death designations utilized were the ICD codes as employed by Office of Population Censuses and Surveys. Six ICD versions were used during the time span considered (ICD-4-9).
**5. Selective reporting bias**		
5.1 Were all measured outcomes reported?	Definitely low	The outcome has been reported in SMR.<br>The outcome provided sufficient data, which could be included in meta-analysis.
**6. Other bias**		
6.1 Conflict of interest	Probably high	Not specified<br>This study was partially supported by the Milbank Memorial Fund, and by BRSG2 S07 RR05392-22 awarded by the Biomedical Research Support Grant Program, Division of Research Resources, National Institutes of Health.

(3) Ewings et al. 1989[19]

Domains	Rating	Comments
**1. Selection bias**		
1.1 Did selection of study participants result in appropriate comparison groups?	Definitely low	The study included residents surrounding Hinkley Point in Somerset down and were recruited from the year, age, and sex.
**2. Confounding bias**		
2.1 Did the study design or analysis account for important confounding and modifying variables?	Probably high	The authors adjusted for age and sex.<br>They did not adjust for other potential confounding factor.
**3. Attrition/Exclusion bias**		
3.1 Were outcome data complete without attrition or exclusion from analysis?	Probably high	Not specified
**4. Detection bias**		
4.1 Can we be confident in the exposure characterization?	Definitely low	The information of smallest areas for which population were identified with postcode areas on the Scottish Cancer Registry since 1968.
4.2 Can we be confident in the outcome assessment?	Probably low	The data of cases were obtained from the South Western Regional Cancer Registry.
**5. Selective reporting bias**		
5.1 Were all measured outcomes reported?	Definitely low	The outcome has been reported in SIR and 95% CI.<br>The outcome provided sufficient data, which could be included in meta-analysis.
**6. Other bias**		
6.1 Conflict of interest	Probably high	Not specified

(4) Urquhart et al. 1991[20]

Domains	Rating	Comments
**1. Selection bias**		
1.1 Did selection of study participants result in appropriate comparison groups?	Definitely low	All registered cases of leukemia and non-Hodgkin's lymphoma in children resident in Caithness during 1968–1986 were included in the study. As controls, the authors selected children who were matched with case children according to sex and date of birth. The controls were also matched according to mother's area of residence at birth; for this purpose, Caithness was divided into two zones: (a) the area within 25 km of the Dounreay nuclear installation and (b) the remainder of Caithness.
**2. Confounding bias**		
2.1 Did the study design or analysis account for important confounding and modifying variables?	Probably high	The authors adjusted for age, caesarean section, breast feeding, birth weight, and smoking during pregnancy.<br>They did not adjust for other potential confounding factors.
**3. Attrition/Exclusion bias**		
3.1 Were outcome data complete without attrition or exclusion from analysis?	Definitely low	Control child data were excluded if they had left the Caithness area before the date of diagnosis for the matched case all results for that control relating to the period after birth were excluded from the analysis.
**4. Detection bias**		
4.1 Can we be confident in the exposure characterization?	Definitely low	Information was collected from each parent by experienced interviewers with a detailed and structured questionnaire. Detailed information was collected for the child and for each parent on all places of residence, on their medical history with particular reference to exposure to x-rays, and on any viral infections.
4.2 Can we be confident in the outcome assessment?	Probably low	The cases included in the study have been the subject of intensive investigation by the Committee on the Medical Aspects of Radiation in the Environment in respect both of completeness of recording and of accuracy of diagnosis.
**5. Selective reporting bias**		
5.1 Were all measured outcomes reported?	Definitely low	The outcome has been reported in OR and 95% CI.<br>The outcome provided sufficient data, which could be included in meta-analysis.
**6. Other bias**		
6.1 Conflict of interest	Probably high	Not specified

(5) Michaelis et al. 1992[21]

Domains	Rating	Comments
**1. Selection bias**		
1.1 Did selection of study participants result in appropriate comparison groups?	Definitely low	The study included residents in 18 locations of nuclear power plants and two of major research reactors which started their operation between 1960 and 1988 in West Germany. The control regions were recruited from different region with the same period.
**2. Confounding bias**		
2.1 Did the study design or analysis account for important confounding and modifying variables?	Probably high	The authors adjusted for age and smoking status of parents. They did not adjust for other potential confounding factor.
**3. Attrition/Exclusion bias**		
3.1 Were outcome data complete without attrition or exclusion from analysis?	Probably high	Not specified
**4. Detection bias**		
4.1 Can we be confident in the exposure characterization?	Definitely low	The region of interest around an individual installation was defined first with a radius of 15 km. Communities with at least one-third of their area falling in this circle form the study area for this particular installation. Subregions corresponding to 5 km and 10 km radii of installation were defined in the same way.
4.2 Can we be confident in the outcome assessment?	Probably low	Diagnoses had to be made between January 1, 1980 (the date the registry started its operation) and December 31, 1990. For the six installations which start their operation after January 1, 1980, inclusion of patients from the installation and control regions was restricted to diagnoses established at least one year after the start-up phase.
**5. Selective reporting bias**		
5.1 Were all measured outcomes reported?	Definitely low	The outcome has been reported in RR, SIR and 95% CI. The outcome provided sufficient data, which could be included in meta-analysis.
**6. Other bias**		
6.1 Conflict of interest	Probably high	Not specified

(6) Goldsmith et al. 1992[22]

Domains	Rating	Comments
**1. Selection bias**		
1.1 Did selection of study participants result in appropriate comparison groups?	Definitely low	The study included the subject in the same period (i.e. 1971–1975 and 1976–1980) and same ages (i.e. 0–9 years)
**2. Confounding bias**		
2.1 Did the study design or analysis account for important confounding and modifying variables?	Probably high	The authors adjusted for age.<br>They did not adjust for other potential confounding factor.
**3. Attrition/Exclusion bias**		
3.1 Were outcome data complete without attrition or exclusion from analysis?	Probably high	Not specified
**4. Detection bias**		
4.1 Can we be confident in the exposure characterization?	Definitely low	The Office of Population Censuses and Surveys (OPCS) data set deals with the information of each site.
4.2 Can we be confident in the outcome assessment?	Probably low	The OPCS data set deals with the information of each site.
**5. Selective reporting bias**		
5.1 Were all measured outcomes reported?	Definitely low	The outcome has been reported in SIR and SMR.<br>The outcome provided sufficient data, which could be included in meta-analysis.
**6. Other bias**		
6.1 Conflict of interest	Probably high	Not specified<br>The study was supported in part by a contract with the European Regional Office of the World Health Organization.

(7) McLaughlin et al. 1993[23]

Domains	Rating	Comments
**1. Selection bias**		
1.1 Did selection of study participants result in appropriate comparison groups?	Definitely low	The study included children, aged 0–14 years in five areas of the province that contain nuclear facilities, who died for leukemia in the period of 1950 to 1987, or were diagnosed with leukemia in the period of 1964 to 1986.<br>The expected numbers of events were based on age-specific rates of childhood leukemia in the province.
**2. Confounding bias**		
2.1 Did the study design or analysis account for important confounding and modifying variables?	Probably high	The authors adjusted for age and facility types.<br>They did not adjust for other potential confounding factor.
**3. Attrition/Exclusion bias**		
3.1 Were outcome data complete without attrition or exclusion from analysis?	Definitely low	Total 287 out of 2,181 childhood leukemia deaths and 560 out of 2,374 childhood leukemia incidence were excluded for clear reasons defined by authors.
**4. Detection bias**		
4.1 Can we be confident in the exposure characterization?	Definitely low	The information of maternal residence at the time of a child's birth was based on Ontario birth certificate.
4.2 Can we be confident in the outcome assessment?	Definitely low	Leukemia was defined according to the 9th version of the ICD (204-208 codes).<br>The information of outcome was collected from the Ontario Cancer Registry, which is a provincewid population-based registry.
**5. Selective reporting bias**		
5.1 Were all measured outcomes reported?	Definitely low	The outcome has been reported in SIR, SMR and 95% CI.<br>The outcome provided sufficient data, which could be included in meta-analysis.
**6. Other bias**		
6.1 Conflict of interest	Probably high	Not specified<br>The study had financial support was received from the Atomic Energy Control Board of Canada.

(8) Hattchouel et al. 1995[24]

Domains	Rating	Comments
**1. Selection bias**		
1.1 Did selection of study participants result in appropriate comparison groups?	Definitely low	The information of cause of each death that occurred in the population aged 0–24 years between 1968 and 1989 by year, zone, sex and five years age groups were collected from the Institut National de la Sante et de la Recherche M&licale (INSERM), service commun no. 8.
**2. Confounding bias**		
2.1 Did the study design or analysis account for important confounding and modifying variables?	Probably high	The authors adjusted for age, sex, facility types and distance from nuclear installations.<br>They did not adjust for other potential confounding factor.
**3. Attrition/Exclusion bias**		
3.1 Were outcome data complete without attrition or exclusion from analysis?	Probably high	Not specified
**4. Detection bias**		
4.1 Can we be confident in the exposure characterization?	Definitely low	Census data by commune were obtained from the Institut National de la Statistique et des Etudes Economiques, for the four censuses which took place in 1968, 1975, 1982 and 1990.
4.2 Can we be confident in the outcome assessment?	Definitely low	The information of cause of each death were collected from the INSERM, service commun no. 8 and were coded according to the 8th and 9th versions of the ICD.
**5. Selective reporting bias**		
5.1 Were all measured outcomes reported?	Definitely low	The outcome has been reported in SMR and 95% CI.<br>The outcome provided sufficient data, which could be included in meta-analysis.
**6. Other bias**		
6.1 Conflict of interest	Probably high	Not specified<br>This work was partly supported by a grant from CEC NRPB (Contract No. 920064).

(9) Morris et al. 1996[25]

Domains	Rating	Comments
**1. Selection bias**		
1.1 Did selection of study participants result in appropriate comparison groups?	Definitely low	The study included subject in same period and region.
**2. Confounding bias**		
2.1 Did the study design or analysis account for important confounding and modifying variables?	Definitely low	The authors adjusted for age, sex, smoking status, socioeconomic status, and work types.
**3. Attrition/Exclusion bias**		
3.1 Were outcome data complete without attrition or exclusion from analysis?	Definitely low	There four qualifying towns were excluded because they were included in a cancer study that was conducted concurrently with the Southeastern Massachusetts Health Study.
**4. Detection bias**		
4.1 Can we be confident in the exposure characterization?	Definitely low	The information of historical data pertaining to residence, occupation, health, and socio-demographics were collected from the subjects or their surrogates for deceased subjects during a 45-minute telephone interview conducted by trained staff.
4.2 Can we be confident in the outcome assessment?	Probably low	The information of cases diagnosed were mainly collected from the Massachusetts Cancer Registry.
**5. Selective reporting bias**		
5.1 Were all measured outcomes reported?	Definitely low	The outcome has been reported in OR and 95% CI.<br>The outcome provided sufficient data, which could be included in meta-analysis.
**6. Other bias**		
6.1 Conflict of interest	Probably high	Not specified

(10) López-Abente et al. 1999[26]

Domains	Rating	Comments
**1. Selection bias**		
1.1 Did selection of study participants result in appropriate comparison groups?	Definitely low	These towns of exposed and reference zones were selected at random from among all of those that met the matching conditions and included 173 and 174 towns, respectively.
**2. Confounding bias**		
2.1 Did the study design or analysis account for important confounding and modifying variables?	Probably low	The authors adjusted for age, sex, and period.
**3. Attrition/Exclusion bias**		
3.1 Were outcome data complete without attrition or exclusion from analysis?	Definitely low	Three plants were excluded because they were experimental research reactors and a fourth because it came into service in 1993.
**4. Detection bias**		
4.1 Can we be confident in the exposure characterization?	Definitely low	The Universal Transversa Mercator projection centroid coordinates for municipal population centers were furnished by the National Geographical Institute, which were used to measure the distance from the population centroids to the nuclear installations.
4.2 Can we be confident in the outcome assessment?	Definitely low	The study presents the results on mortality according to the 8th and 9th versions of the ICD.
**5. Selective reporting bias**		
5.1 Were all measured outcomes reported?	Definitely low	The outcome has been reported in RR, SMR and 95% CI.<br>The outcome provided sufficient data, which could be included in meta-analysis.
**6. Other bias**		
6.1 Conflict of interest	Probably high	Not specified<br>The study was supported in part by Grant 96/300 from Spain's Fondo de Investigación Sanitaria (Health Research Fund).

(11) López-Abente et al. 2001[27]

Domains	Rating	Comments
**1. Selection bias**		
1.1 Did selection of study participants result in appropriate comparison groups?	Definitely low	There were 184 towns within 30 km of installation were called the "exposed zone"; and towns there were 178 towns within 50–100 km of the installation were called the "reference zone."
**2. Confounding bias**		
2.1 Did the study design or analysis account for important confounding and modifying variables?	Probably low	The authors adjusted for age, sex, and period.
**3. Attrition/Exclusion bias**		
3.1 Were outcome data complete without attrition or exclusion from analysis?	Probably high	Not specified
**4. Detection bias**		
4.1 Can we be confident in the exposure characterization?	Definitely low	The authors used the Universal Transversa Mercator projection centroid coordinates for towns with a geographic information system to measure the distance from the population centroids to the nuclear installations.
4.2 Can we be confident in the outcome assessment?	Definitely low	The study presents the results on mortality according to the 9th version of the ICD.
**5. Selective reporting bias**		
5.1 Were all measured outcomes reported?	Definitely low	The outcome has been reported in RR, SMR and 95% CI.<br>The outcome provided sufficient data, which could be included in meta-analysis.
**6. Other bias**		
6.1 Conflict of interest	Probably high	Not specified<br>The study was supported in part by Grant 96/300 from Spain's Fondo de Investigación Sanitaria (Health Research Fund).

(12) Silva-Mato et al. 2003[28]

Domains	Rating	Comments
**1. Selection bias**		
1.1 Did selection of study participants result in appropriate comparison groups?	Definitely low	The study included cases were patients with cancer and controls were non-tumorous patients, both admitted to the Guadalajara hospital, who living in the areas within 10, 20, and 30 km of each plant.
**2. Confounding bias**		
2.1 Did the study design or analysis account for important confounding and modifying variables?	Probably high	The authors adjusted for age, sex, and zones.<br>Using indicates measures to derive smoking.
**3. Attrition/Exclusion bias**		
3.1 Were outcome data complete without attrition or exclusion from analysis?	Probably high	Not specified
**4. Detection bias**		
4.1 Can we be confident in the exposure characterization?	Definitely low	The information source used for pathology as well as for place of residence is the computerized database of discharge reports from hospital, completed for each patient admitted, provided by the Admission and Clinical Documentation Services of the hospital.<br>The authors performed a clear criterion to evaluate the variable of degree of proximity of place of residence to the nuclear power stations.
4.2 Can we be confident in the outcome assessment?	Probably low	The cases included were all patients with malignant tumors admitted to the Guadalajara hospital in the period of December 27, 1988 to January 16, 1999. Inclusion criteria were admittance for non-secondary malignant tumor pathology, and residence in the province.
**5. Selective reporting bias**		
5.1 Were all measured outcomes reported?	Definitely low	The outcome has been reported in OR and 95% CI.<br>The outcome provided sufficient data, which could be included in meta-analysis.
**6. Other bias**		
6.1 Conflict of interest	Probably high	Not specified<br>This research was supported by the University of Alcalá, grant number E017/97.

(13) Mangano et al. 2003[29]

Domains	Rating	Comments
**1. Selection bias**		
1.1 Did selection of study participants result in appropriate comparison groups?	Definitely low	The study included childhood age < 10 years in 49 counties who resided in the study counties at the time of diagnosis situated mostly or completely within 30 miles (i.e. 48 km) of the nuclear reactors in the eastern US.<br>The results would compare with national cancer rates.
**2. Confounding bias**		
2.1 Did the study design or analysis account for important confounding and modifying variables?	Probably high	The authors adjusted for age.<br>They did not adjust for other potential confounding factor.
**3. Attrition/Exclusion bias**		
3.1 Were outcome data complete without attrition or exclusion from analysis?	Probably high	Not specified
**4. Detection bias**		
4.1 Can we be confident in the exposure characterization?	Definitely low	The study used data from some of registries in the eastern United States.
4.2 Can we be confident in the outcome assessment?	Definitely low	The information of childhood cancer mortality data was according to the 9th version of the ICD (140.0–239.9 codes), which was available for all states from 1988 to 1997 and were obtained from the US Centers for Disease Control and Prevention.
**5. Selective reporting bias**		
5.1 Were all measured outcomes reported?	Definitely low	The outcome has been reported in death rate, incidence rate and 95% CI.<br>The outcome provided sufficient data, which could be included in meta-analysis.
**6. Other bias**		
6.1 Conflict of interest	Probably high	Not specified

(14) White-Koning et al. 2004[30]

Domains	Rating	Comments
**1. Selection bias**		
1.1 Did selection of study participants result in appropriate comparison groups?	Definitely low	The study included the incidence of leukemia among children under 15 years of age living within 20 km of 29 nuclear installations in France and used national age-specific incidence rates to derive annual expected numbers of cases for each age group.
**2. Confounding bias**		
2.1 Did the study design or analysis account for important confounding and modifying variables?	Probably low	The authors adjusted for age sex and stratified by distance.
**3. Attrition/Exclusion bias**		
3.1 Were outcome data complete without attrition or exclusion from analysis?	Probably high	Not specified
**4. Detection bias**		
4.1 Can we be confident in the exposure characterization?	Definitely low	The study areas were constructed as aggregations of the communes whose town hall was within the defined zones.
4.2 Can we be confident in the outcome assessment?	Probably low	The information of cases of acute leukemia diagnosed the in period of January 1, 1990 to December 31, 1998 among children aged < 15 years living within the study area at diagnosis were provided by the National Registry of Childhood Leukemia and Lymphoma, which has registered all cases diagnosed in France since January 1, 1990.
**5. Selective reporting bias**		
5.1 Were all measured outcomes reported?	Definitely low	The outcome has been reported in SIR and 95% CI.<br>The outcome provided sufficient data, which could be included in meta-analysis.
**6. Other bias**		
6.1 Conflict of interest	Probably high	Not specified<br>This study was supported by grants from INSERM, the Direction Générale de la Santé, the Ministère de l'Environnement et de l'Aménagement du Territoire and the Fondation pour la Recherche Médicale.

(15) Yoshimoto et al. 2004[31]

Domains	Rating	Comments
**1. Selection bias**		
1.1 Did selection of study participants result in appropriate comparison groups?	Definitely low	The study included 20 administrative areas (cities/towns/villages) in Japan where commercial operation of the first reactor started between 1966 and 1993.<br>There 80 control areas were chosen with respect to similarity of administrative area unit, population size, and seashore area so that they could be assumed to have a similar environment/lifestyle, which have distance between nuclear facility and the control areas was about 10–80 km.
**2. Confounding bias**		
2.1 Did the study design or analysis account for important confounding and modifying variables?	Probably high	The study adjusted for temporal area variations associated with Kyushu and the other five local area blocks and did not for other potential confounding factor.
**3. Attrition/Exclusion bias**		
3.1 Were outcome data complete without attrition or exclusion from analysis?	Definitely low	Exclude chronic lymphocytic leukemia (CLL) (non-CLL leukemia) and non-Hodgkin's lymphoma mortality.
**4. Detection bias**		
4.1 Can we be confident in the exposure characterization?	Definitely low	The information of census data (for 1970, 1975, 1980, 1985, 1990, and 1995) were provided by the Statistics Bureau, Management and Coordination Agency, Government of Japan—the present Ministry of Public Management, Home Affairs, Posts and Telecommunications, and linearly interpolated or non-negatively extrapolated numbers were used for each year between 1972 and 1997.
4.2 Can we be confident in the outcome assessment?	Definitely low	The observed numbers of deaths were counted using the following the ICD codes for vital statistics mortality data provided by the Statistics and Information Department, Minister's Secretariat, Ministry of Health and Welfare.
**5. Selective reporting bias**		
5.1 Were all measured outcomes reported?	Definitely low	The outcome has been reported in RR and 95% CI.<br>The outcome provided sufficient data, which could be included in meta-analysis.
**6. Other bias**		
6.1 Conflict of interest	Probably high	Not specified

(16) Boice et al. 2005[32]

Domains	Rating	Comments
**1. Selection bias**		
1.1 Did selection of study participants result in appropriate comparison groups?	Definitely low	The study included childhood aged < 20 years in St Lucie County, which located on the east coast of Florida, south of Cape Canaveral.<br>The comparison counties were defined as Polk and Volusia Counties for having similar demographic data with St Lucie County.
**2. Confounding bias**		
2.1 Did the study design or analysis account for important confounding and modifying variables?	Probably high	The authors adjusted for age and sex.<br>They did not adjust for other potential confounding factor but chose comparison counties from the same region as the study county helps minimize differences in these and other factors, such as diet and smoking.
**3. Attrition/Exclusion bias**		
3.1 Were outcome data complete without attrition or exclusion from analysis?	Definitely low	Analyses excluding the first 10 years after reactor start-up in 1976.
**4. Detection bias**		
4.1 Can we be confident in the exposure characterization?	Definitely low	The 1990 Census Bureau demographic data on ten socioeconomic variables were obtained for St Lucie County and for all other counties in Florida.
4.2 Can we be confident in the outcome assessment?	Definitely low	The outcome data was classified by the 8th version of the ICD.
**5. Selective reporting bias**		
5.1 Were all measured outcomes reported?	Definitely low	The outcome has been reported in RR, SMR and 95% CI.<br>The outcome provided sufficient data, which could be included in meta-analysis.
**6. Other bias**		
6.1 Conflict of interest	Probably high	Not specified<br>The study was funded, in part, by an agreement with the American Nuclear Insurers.

(17) Kaatsch et al. 2008[33]

Domains:	Rating	Comments
**1. Selection bias**		
1.1 Did selection of study participants result in appropriate comparison groups?	Definitely low	The study included the cases of all the children under 5 years of age in 41 administrative districts in the vicinity of 16 nuclear power plants in western Germany.<br>For every case, three sex- and age-matched controls were randomly selected from the same district in the year of diagnosis, which totally included 4,735 controls.
**2. Confounding bias**		
2.1 Did the study design or analysis account for important confounding and modifying variables?	Probably high	The authors did not adjust for potential confounding factor.
**3. Attrition/Exclusion bias**		
3.1 Were outcome data complete without attrition or exclusion from analysis?	Probably high	Not specified
**4. Detection bias**		
4.1 Can we be confident in the exposure characterization?	Definitely low	For both cases and controls the residential address at the time of diagnosis was geo-coded and the distance "r" to the outlet tower of the nearest nuclear power plant established.<br>The model that authors used to estimate the radiation exposure was according to the United Nations Scientific Committee on the Effects of Atomic Radiation, which included the function of distance from a corresponding point source, and was as a variable in the regression model.
4.2 Can we be confident in the outcome assessment?	Probably low	The information of cases was on the basis of data from the German Childhood Cancer Registry.
**5. Selective reporting bias**		
5.1 Were all measured outcomes reported?	Definitely low	The outcome has been reported in OR, SIR and 95% CI.<br>The outcome provided sufficient data, which could be included in meta-analysis.
**6. Other bias**		
6.1 Conflict of interest	Definitely low	The authors declare that no conflict of interest exists according to the guidelines of the International Committee of Medical Journal Editors.<br>The study was supported by the Federal Ministry for the Environment, Nature Conservation and Nuclear Safety and the Federal Office for Radiation Protection (BfS) for financing the KiKK study (project no. StSch 4334) and to the Expert Commission of the BfS.

(18) Zadnik et al. 2008[34]

Domains	Rating	Comments
**1. Selection bias**		
1.1 Did selection of study participants result in appropriate comparison groups?	Definitely low	The study included data of risk in same period in Spodnjeposavska statistical region, where Brežice municipality and Krško nuclear power plant are situated and were compared with risk for other 11 Slovenian statistical regions.
**2. Confounding bias**		
2.1 Did the study design or analysis account for important confounding and modifying variables?	Probably high	The authors adjusted for age.<br>They did not adjust for other potential confounding factor.
**3. Attrition/Exclusion bias**		
3.1 Were outcome data complete without attrition or exclusion from analysis?	Definitely low	The cases of CLL were excluded as authors thought CLL is less inducible by ionizing radiation than other types of leukemia.
**4. Detection bias**		
4.1 Can we be confident in the exposure characterization?	Definitely low	Cancer incidence data including cancer site, sex, age at diagnosis, and place of residence at the time of diagnosis were collected for two time periods: 1970–1983 and 1984–2003 and provided by the Cancer Registry of Slovenia.
4.2 Can we be confident in the outcome assessment?	Probably low	The data on cancer incidence was obtained from the population-based Cancer Registry of Slovenia, existing since 1950, which main sources are notifications of cancer prescribed by the law.<br>Coding of the information is performed by trained registrars, supervised by a physician. The coding rules and standards are following the World Health Organization recommendations.
**5. Selective reporting bias**		
5.1 Were all measured outcomes reported?	Definitely low	The outcome has been reported in RR and 95% CI.<br>The outcome provided sufficient data, which could be included in meta-analysis.
**6. Other bias**		
6.1 Conflict of interest	Probably high	Not specified<br>The study was financed by the Agency for Radwaste Management of the Republic of Slovenia.

(19) Spix et al. 2009[35]

Domains	Rating	Comments
**1. Selection bias**		
1.1 Did selection of study participants result in appropriate comparison groups?	Definitely low	The study included the cases aged < 5 years at diagnosis in the period of 1993 to 2003, had been registered at the German. The matching criteria of cases and controls were sex, age, and year of diagnosis. Total 366 cases and 896 controls were available for analysis, these are one to seven controls per case, on average 2.5 controls per case.
**2. Confounding bias**		
2.1 Did the study design or analysis account for important confounding and modifying variables?	Probably low	The authors adjusted for sex, age, socioeconomic status, duration of pregnancy, birth weight, year at diagnosis, and toxins.
**3. Attrition/Exclusion bias**		
3.1 Were outcome data complete without attrition or exclusion from analysis?	Definitely low	The reasons of exclusion during matching the cases and controls included non-response, the child received a therapy with ionizing radiation before the diagnosis, suffered from an autoimmune disease, had an organ transplant, regularly took immune suppressive drugs.
**4. Detection bias**		
4.1 Can we be confident in the exposure characterization?	Definitely low	The questions of questionnaire referred to social status, potential exposure with ionizing radiation, aspects of pregnancy and birth of the child, activation of the child's immune system and toxins. The questions would use to interview the biological mother and the interviewers were trained in multiple sessions and met regularly for retraining over the about 18 months of the field work.
4.2 Can we be confident in the outcome assessment?	Probably low	The diagnose data were provided by the German Childhood Cancer Registry.
**5. Selective reporting bias**		
5.1 Were all measured outcomes reported?	Definitely low	The outcome has been reported in OR and 95% CI.<br>The outcome provided sufficient data, which could be included in meta-analysis.
**6. Other bias**		
6.1 Conflict of interest	Definitely low	The authors declare that have no conflict of interest to disclose.

(20) Mangano et al. 2009[36]

Domains	Rating	Comments
**1. Selection bias**		
1.1 Did selection of study participants result in appropriate comparison groups?	Definitely low	The study included the data of thyroid cancer incidence 45 states plus the District of Columbia in same period.
**2. Confounding bias**		
2.1 Did the study design or analysis account for important confounding and modifying variables?	Probably high	The authors adjusted for age.<br>They did not adjust for other potential confounding factor.
**3. Attrition/Exclusion bias**		
3.1 Were outcome data complete without attrition or exclusion from analysis?	Definitely low	Incidence rates are published only for those counties with at least 15 thyroid cancer cases diagnosed in the period of 2001 to 2005, as rates in less-populated counties are based on small numbers of cases that are often not reliable.
**4. Detection bias**		
4.1 Can we be confident in the exposure characterization?	Definitely low	The US Centers for Disease Control and Prevention or the National Cancer Institute (NCI).
4.2 Can we be confident in the outcome assessment?	Probably low	The US Centers for Disease Control and Prevention or the NCI.
**5. Selective reporting bias**		
5.1 Were all measured outcomes reported?	Definitely low	The outcome has been reported in incidence rate and 95% CI. The outcome provided sufficient data, which could be included in meta-analysis.
**6. Other bias**		
6.1 Conflict of interest	Probably high	Not specified

(21) Heinävaara et al. 2010[37]

Domains	Rating	Comments
**1. Selection bias**		
1.1 Did selection of study participants result in appropriate comparison groups?	Definitely low	The study included subject in same period and region.
**2. Confounding bias**		
2.1 Did the study design or analysis account for important confounding and modifying variables?	Probably low	The authors adjusted for age, sex, socioeconomic status, parents' radiation work, and father's age at child's birth.
**3. Attrition/Exclusion bias**		
3.1 Were outcome data complete without attrition or exclusion from analysis?	Definitely low	In case–control analysis:<br>In children, the coordinates of residential history were missing for 8% (i.e. three out of 38 coordinates) of cases and 4% (i.e. five out of 128 coordinates) of controls, and in adults, the corresponding percentages were 6% (i.e. 12 out of 196) and 11% (i.e. 89 out of 828), respectively.<br>The coordinates were missing in children for one case and one control, and in adults for eight cases and 24 controls.
**4. Detection bias**		
4.1 Can we be confident in the exposure characterization?	Definitely low	Residential histories of all study subjects were obtained from the Population Register Center.
4.2 Can we be confident in the outcome assessment?	Definitely low	Leukemia cases were according to the 10th version of the ICD (C91-95 coded) and were identified from the Finnish Cancer Registry.
**5. Selective reporting bias**		
5.1 Were all measured outcomes reported?	Definitely low	The outcome has been reported in RR, SIR, OR and 95% CI. The outcome provided sufficient data, which could be included in meta-analysis.
**6. Other bias**		
6.1 Conflict of interest	Definitely low	The authors declared that none of the authors have any financial or non-financial competing interests.

(22) Spycher et al. 2011[38]

Domains	Rating	Comments
**1. Selection bias**		
1.1 Did selection of study participants result in appropriate comparison groups?	Definitely low	The study included childhood aged 0–15 who living within 15 km of five nuclear power plants (Beznau I and II, Leibstadt, Mühleberg and Gösgenin) in Switzerland.<br>The reference population was defined as childhood aged 0–15 who living farther than 15 km from five nuclear power plants in Switzerland, which has been adjusted for sex, age and calendar year.
**2. Confounding bias**		
2.1 Did the study design or analysis account for important confounding and modifying variables?	Probably low	The authors adjusted for age, sex, and calendar year at diagnosis.
**3. Attrition/Exclusion bias**		
3.1 Were outcome data complete without attrition or exclusion from analysis?	Definitely low	The 1,250 and 85 children excluded from birth and resident cohorts, respectively, had higher proportions of lymphomas and bone tumors as they were, on average, older at diagnosis.
**4. Detection bias**		
4.1 Can we be confident in the exposure characterization?	Definitely low	The authors have clearly interpreted the measurement or estimation of exposure assessment and geo-coding in study.
4.2 Can we be confident in the outcome assessment?	Definitely low	The data of children diagnosed with cancer in the period of 1985 to 2009 were based on the Swiss Childhood Cancer Registry were eligible, which has completeness of registration is > 90% and were classified according to the 3rd version of the International Classification of Childhood Cancer (ICCC).
**5. Selective reporting bias**		
5.1 Were all measured outcomes reported?	Definitely low	The outcome has been reported in RR and 95% CI.<br>The outcome provided sufficient data, which could be included in meta-analysis.
**6. Other bias**		
6.1 Conflict of interest	Definitely low	The authors declare that no conflict of interest in study.<br>This study was supported by the Swiss Federal Office of Public Health (BAG 08.001616) and the Swiss Cancer League (KLS 02224-03-2008).

(23) Ma et al. 2011[39]

Domains	Rating	Comments
**1. Selection bias**		
1.1 Did selection of study participants result in appropriate comparison groups?	Definitely low	The study included cancer cases diagnosed in the period of 1986 to 2005 for Illinois children aged 0–14 years.<br>The expected number of cases for each geographic zone was obtained using the Illinois state age-specific cancer incidence for the two periods (i.e. 1986–1995 and 1996–2005) and the corresponding census populations.
**2. Confounding bias**		
2.1 Did the study design or analysis account for important confounding and modifying variables?	Probably high	The authors adjusted for age.<br>They did not adjust for other potential confounding factor.
**3. Attrition/Exclusion bias**		
3.1 Were outcome data complete without attrition or exclusion from analysis?	Probably high	Not specified
**4. Detection bias**		
4.1 Can we be confident in the exposure characterization?	Definitely low	The authors used distance from nuclear power plants to the zone improvement plan code centroid of cancer case residence as the surrogate of exposure, which were calculated using SAS program developed by the North American Association of Central Cancer Registries.
4.2 Can we be confident in the outcome assessment?	Definitely low	Individual-level cancer incidence data were from the Illinois State Cancer Registry and were classified by the 3rd version of the ICCC.
**5. Selective reporting bias**		
5.1 Were all measured outcomes reported?	Definitely low	The outcome has been reported in RR, SIR and 95% CI.<br>The outcome provided sufficient data, which could be included in meta-analysis.
**6. Other bias**		
6.1 Conflict of interest	Probably high	Not specified

(24) Bazyka et al. 2012[40]

Domains	Rating	Comments
**1. Selection bias**		
1.1 Did selection of study participants result in appropriate comparison groups?	Definitely low	The study included the case data of malignant tumors in the population of the listed cities in same period, which were provided from the National Cancer Registry of Ukraine.
**2. Confounding bias**		
2.1 Did the study design or analysis account for important confounding and modifying variables?	Probably high	The authors adjusted for age.<br>They did not adjust for other potential confounding factor.
**3. Attrition/Exclusion bias**		
3.1 Were outcome data complete without attrition or exclusion from analysis?	Definitely low	Excluded some cities and were listed in table.
**4. Detection bias**		
4.1 Can we be confident in the exposure characterization?	Definitely low	The study was area-based and the total number of inhabitants of the cities of Nuclear Energy and the reported cancer cases are presented in table.
4.2 Can we be confident in the outcome assessment?	Definitely low	The data on primary cancer cases of the National Cancer Registry of Ukraine conducted by the National Cancer Institute were used and were classified by the 10th version of ICD.
**5. Selective reporting bias**		
5.1 Were all measured outcomes reported?	Definitely low	The outcome has been reported in SIR and 95% CI.<br>The outcome provided sufficient data, which could be included in meta-analysis.
**6. Other bias**		
6.1 Conflict of interest	Probably high	Not specified.

(25) Ahn et al. 2012[41]

Domains	Rating	Comments
**1. Selection bias**		
1.1 Did selection of study participants result in appropriate comparison groups?	Definitely low	The exposed population were based on the Korea Radiation Effect & Epidemiology Cohort included adult residents living within 5 km of the nuclear power plants. The non-exposed population was set at two different levels of proximity: 5–30 and 30 km from nuclear power plants. Both of them were recruited from same period and region
**2. Confounding bias**		
2.1 Did the study design or analysis account for important confounding and modifying variables?	Definitely low	The authors adjusted for age, sex, smoking, alcohol drinking, liver disease history, current job, education attainment, total energy expenditure per day, family history of cancer, body mass index, and medical services of x-rays or radiation.
**3. Attrition/Exclusion bias**		
3.1 Were outcome data complete without attrition or exclusion from analysis?	Definitely low	Cases with codes other than C00-D09 were excluded from the screening, because a trial sample survey on the probability of having cancer among those cases yielded nil.
**4. Detection bias**		
4.1 Can we be confident in the exposure characterization?	Definitely low	The definition exposed study area was based on the "Act on Support to the around Area of Power Plants", which was enacted in June 1989.<br>An independent radiological investigation in the residential areas near nuclear power plants, the exposed study areas, by Thermo-luminescence dosimeter at 7-8 spots in each exposed study areas.
4.2 Can we be confident in the outcome assessment?	Definitely low	The outcome data was defined as C00~D09 according to the 10th version of the ICD-10.
**5. Selective reporting bias**		
5.1 Were all measured outcomes reported?	Definitely low	The outcome has been reported in HR, CR and ASR cancer incidence rate and 95% CI.<br>The outcome provided sufficient data, which could be included in meta-analysis.
**6. Other bias**		
6.1 Conflict of interest	Probably high	Not specified<br>This study was supported in part by the National Research Foundation of Korea (NRF) funded by the Republic of Korea Government (Ministry of Education, Science and Technology) (No. 2010-0000986).

(26) Sermage-Faure et al. 2012[42]

Domains	Rating	Comments
**1. Selection bias**		
1.1 Did selection of study participants result in appropriate comparison groups?	Definitely low	The study included all the 2,753 French childhood leukemia cases aged up to 15 years at the end of the year of diagnosis, diagnosed in the period of 2002 to 2007, which were obtained from the French National Registry of Childhood Hematopoietic Malignancies (NRCH).
**2. Confounding bias**		
2.1 Did the study design or analysis account for important confounding and modifying variables?	Probably low	The authors adjusted for age at the end of the year and some demographic variables.
**3. Attrition/Exclusion bias**		
3.1 Were outcome data complete without attrition or exclusion from analysis?	Definitely low	Analyses were also performed after excluding the subjects living 50 km from nuclear power plants in the case–control study and computing the incidence between 20 and 50 km.
**4. Detection bias**		
4.1 Can we be confident in the exposure characterization?	Definitely low	The addresses of the cases and controls were geocoded by the GEOCIBLE Company (Saint-Maurice/France) using the geographic information system MAPINFO (New York/USA), the NAVTEQ (Paris/France) street databases and detailed vectorized maps from the National Geographic Institute (Saint-Mandé/France).
4.2 Can we be confident in the outcome assessment?	Probably low	The outcome data were obtained from the French NRCH.
**5. Selective reporting bias**		
5.1 Were all measured outcomes reported?	Definitely low	The outcome has been reported in SIR and 95% CI.<br>The outcome provided sufficient data, which could be included in meta-analysis.
**6. Other bias**		
6.1 Conflict of interest	Probably high	Not specified<br>This study was supported by Institut de Veille Sanitaire (InVS), Agence Nationale de S'ecurit'e Sanitaire de l'Alimentation, de l'Environnement et du Travail (ANSES), Association pour la Recherche sur le Cancer (ARC), Fondation Pfizer, Institut National du Cancer (INCa), Agence Nationale de la Recherche (ANR).

(27) Bithell et al. 2013[43]

Domains	Rating	Comments
**1. Selection bias**		
1.1 Did selection of study participants result in appropriate comparison groups?	Definitely low	The study included cases in same period and region from the National Registry of Childhood Tumours (NRCT).
**2. Confounding bias**		
2.1 Did the study design or analysis account for important confounding and modifying variables?	Probably high	Not specified
**3. Attrition/Exclusion bias**		
3.1 Were outcome data complete without attrition or exclusion from analysis?	Definitely low	The study excluded the Sellafield site based on the reasons given in the Committee on Medical Aspects of Radiation in the Environment 2011.
**4. Detection bias**		
4.1 Can we be confident in the exposure characterization?	Definitely low	For over 90% of the cases and controls, the grid reference of the actual residential address at birth could be located using the Ordnance Survey product, ADDRESS-POINT; for the remainder, the postcode was used provided it determined the location to an accuracy equivalent at least to the approximate house number.<br>The authors defined proximity to an nuclear power plant as the reciprocal of the distance in kilometer from residential address at birth or diagnosis to the nearest of these plants commissioned before the case child was born.
4.2 Can we be confident in the outcome assessment?	Definitely low	The cancers data were provided from the NRCT and were classified using the 3rd version of the ICCC.
**5. Selective reporting bias**		
5.1 Were all measured outcomes reported?	Definitely low	The outcome has been reported in OR and 95% CI.<br>The outcome provided sufficient data, which could be included in meta-analysis.
**6. Other bias**		
6.1 Conflict of interest	Definitely high	One of author carries out paid consultancy work, including work for the nuclear industry.

(28) Lane et al. 2013[44]

Domains	Rating	Comments
**1. Selection bias**		
1.1 Did selection of study participants result in appropriate comparison groups?	Definitely low	The study included the cancer incidence data from 1990 to 2008 for populations living within 25 km of three Ontario nuclear power plants for observed group and the general Ontario population for expected group, both of them were provided by the Ontario Cancer Registry (OCR) and the Canadian Cancer Registry (CCR).
**2. Confounding bias**		
2.1 Did the study design or analysis account for important confounding and modifying variables?	Probably high	The authors adjusted for age and sex.<br>They did not adjust for other potential confounding factor.
**3. Attrition/Exclusion bias**		
3.1 Were outcome data complete without attrition or exclusion from analysis?	Probably high	Not specified
**4. Detection bias**		
4.1 Can we be confident in the exposure characterization?	Definitely low	The authors have clearly interpreted the measurement or estimation of dose calculations among the public living near Ontario nuclear power plants in study.
4.2 Can we be confident in the outcome assessment?	Definitely low	The case data was collected from the OCR from 1990 to 1991 and the CCR from 1992 to 2008 and selected according to 3rd version of the ICCC.
**5. Selective reporting bias**		
5.1 Were all measured outcomes reported?	Definitely low	The outcome has been reported in SIR and 95% CI.<br>The outcome provided sufficient data, which could be included in meta-analysis.
**6. Other bias**		
6.1 Conflict of interest	Probably high	Not specified

(29) Bollaerts et al. 2014[45]

Domains	Rating	Comments
**1. Selection bias**		
1.1 Did selection of study participants result in appropriate comparison groups?	Definitely low	The study included the thyroid cancer incidence data in same period of the nuclear sites of interest.
**2. Confounding bias**		
2.1 Did the study design or analysis account for important confounding and modifying variables?	Probably high	The authors adjusted for age and sex.<br>They did not adjust for other potential confounding factor.
**3. Attrition/Exclusion bias**		
3.1 Were outcome data complete without attrition or exclusion from analysis?	Probably high	Not specified
**4. Detection bias**		
4.1 Can we be confident in the exposure characterization?	Definitely low	For each of the 589 Belgian communes, distances were calculated between the commune's centroid and the geographical location of the nuclear sites. The locations of the sites were determined by locating the nuclear facilities.
4.2 Can we be confident in the outcome assessment?	Definitely low	Thyroid cancer incidence data according to the 10th version of the ICD (C73 code) were requested from the Belgian Cancer Registry (BCR).
**5. Selective reporting bias**		
5.1 Were all measured outcomes reported?	Definitely low	The outcome has been reported in RR, SIR and 95% CI.<br>The outcome provided sufficient data, which could be included in meta-analysis.
**6. Other bias**		
6.1 Conflict of interest	Definitely low	The authors declared that no competing financial interests exist in study.

(30) Wang et al. 2016[46]

Domains	Rating	Comments
**1. Selection bias**		
1.1 Did selection of study participants result in appropriate comparison groups?	Definitely low	The study subjects included in the present study were inhabitant living in the eight townships interested. The Cancer Registry Database identified every patient by county and village of residence.
**2. Confounding bias**		
2.1 Did the study design or analysis account for important confounding and modifying variables?	Probably high	The authors adjusted for age and sex.<br>They did not adjust for other potential confounding factor.
**3. Attrition/Exclusion bias**		
3.1 Were outcome data complete without attrition or exclusion from analysis?	Probably high	Not specified
**4. Detection bias**		
4.1 Can we be confident in the exposure characterization?	Definitely low	The "plant-vicinity" group included inhabitants in the townships where nuclear power plants are located. The "non-plant-vicinity" group was chosen based on the New Taipei City General Development Plan and the Pingtung County Revised General Development Plan.
4.2 Can we be confident in the outcome assessment?	Definitely low	The cancer data was collected from the Taiwan Cancer Registry Database compiled by the Health Promotion Administration, Ministry of Health and Welfare, which is coded using the International Classification of Diseases for Oncology (ICD-O), field trial edition for 1979–2001 and the ICD-O, third edition after 2002.
**5. Selective reporting bias**		
5.1 Were all measured outcomes reported?	Definitely low	The outcome has been reported in RR and 95% CI.<br>The outcome provided sufficient data, which could be included in meta-analysis.
**6. Other bias**		
6.1 Conflict of interest	Definitely low	The authors declare that they have no conflict of interest.

(31) Khomenko et al. 2017[47]

Domains	Rating	Comments
**1. Selection bias**		
1.1 Did selection of study participants result in appropriate comparison groups?	Definitely low	The study included adults aged 18 years and over in Dnipropetrovsk region as observation zone, where the Zaporizhzhia nuclear power plant located, and Petrivsk district as controls zone in same period.
**2. Confounding bias**		
2.1 Did the study design or analysis account for important confounding and modifying variables?	Probably high	Not specified
**3. Attrition/Exclusion bias**		
3.1 Were outcome data complete without attrition or exclusion from analysis?	Probably high	Not specified
**4. Detection bias**		
4.1 Can we be confident in the exposure characterization?	Definitely low	The state statistical reporting data included into the form of the Ministry of Health of Ukraine no.12 «Report on the Diseases Registered in Patients Living in the Area Serviced by the Treatment and Prophylactic Establishment» were the information base for the analysis. The data were generalized by districts, Dnipropetrovsk region and overall, by Ukraine.
4.2 Can we be confident in the outcome assessment?	Definitely low	The outcome data was classified by the 10th version of the ICD.
**5. Selective reporting bias**		
5.1 Were all measured outcomes reported?	Definitely low	The outcome has been reported in morbidity rate. The outcome provided sufficient data, which could be included in meta-analysis.
**6. Other bias**		
6.1 Conflict of interest	Probably high	Not specified

(32) Demoury et al. 2017[48]

Domains	Rating	Comments
**1. Selection bias**		
1.1 Did selection of study participants result in appropriate comparison groups?	Definitely low	The study included the data in the Flemish region from 2000 to 2014 and the Brussels-Capital region and the Walloon region from 2004 to 2014, which were selected from the Belgian Cancer Registry (BCR), a national population-based registry.
**2. Confounding bias**		
2.1 Did the study design or analysis account for important confounding and modifying variables?	Probably low	The authors adjusted for age, sex, incidence year and region.
**3. Attrition/Exclusion bias**		
3.1 Were outcome data complete without attrition or exclusion from analysis?	Probably high	Not specified
**4. Detection bias**		
4.1 Can we be confident in the exposure characterization?	Definitely low	The authors have clearly interpreted the measurement or estimation of surrogate exposure in study.
4.2 Can we be confident in the outcome assessment?	Probably low	Thyroid cancer incidence data were selected from the BCR, a national population-based registry.
**5. Selective reporting bias**		
5.1 Were all measured outcomes reported?	Definitely low	The outcome has been reported in RR and 95% CI.<br>The outcome provided sufficient data, which could be included in meta-analysis.
**6. Other bias**		
6.1 Conflict of interest	Definitely low	The authors declare no conflict of interest.

(33) Desbiolles et al. 2018[49]

Domains	Rating	Comments
**1. Selection bias**		
1.1 Did selection of study participants result in appropriate comparison groups?	Definitely low	The study was conducted around nuclear power plants at the level of the municipality, the smallest French administrative unit in rural areas and included adults aged ≥ 15 years, who was diagnosed in the period of 1995 to 2011.
**2. Confounding bias**		
2.1 Did the study design or analysis account for important confounding and modifying variables?	Definitely low	The authors adjusted for age, sex, tobacco and alcohol consumption, deprivation, rural/urban status, population density, and exposure to pesticides, road traffic pollution, other polluting industries, benzene and high-voltage power lines.
**3. Attrition/Exclusion bias**		
3.1 Were outcome data complete without attrition or exclusion from analysis?	Definitely low	Excluding any cancers for the Chooz nuclear power plant.
**4. Detection bias**		
4.1 Can we be confident in the exposure characterization?	Definitely low	The surrogate exposure measure was the distance between the nuclear power plant and the municipality's town hall. The municipalities were considered potentially exposed to nuclear power plants if they were within 20 km of plants; the area was called the "20-km proximity area".
4.2 Can we be confident in the outcome assessment?	Definitely low	The outcome data was classified by the 3rd version of the ICD-O.
**5. Selective reporting bias**		
5.1 Were all measured outcomes reported?	Definitely low	The outcome has been reported in RR and 95% CI.<br>The outcome provided sufficient data, which could be included in meta-analysis.
**6. Other bias**		
6.1 Conflict of interest	Probably high	Not specified

(34) Demoury et al. 2021[50]

Domains	Rating	Comments
**1. Selection bias**		
1.1 Did selection of study participants result in appropriate comparison groups?	Definitely low	The study included the case of children diagnosed with acute lymphoid or myeloid leukemia in the period of 2006 to 2016 and aged < 15 years at the time of diagnosis were included in the study, provided by the Belgian Cancer Registry (BCR).
**2. Confounding bias**		
2.1 Did the study design or analysis account for important confounding and modifying variables?	Definitely low	The authors adjusted for age, sex, socio-economic index and median household income.
**3. Attrition/Exclusion bias**		
3.1 Were outcome data complete without attrition or exclusion from analysis?	Probably high	Not specified
**4. Detection bias**		
4.1 Can we be confident in the exposure characterization?	Definitely low	The authors have clearly interpreted the measurement or estimation of surrogate exposure in study.
4.2 Can we be confident in the outcome assessment?	Definitely low	The case data was collected from the BCR, a national population-based registry, and selected according to the ICD-O-3 codes.
**5. Selective reporting bias**		
5.1 Were all measured outcomes reported?	Definitely low	The outcome has been reported in RR and 95% CI. The outcome provided sufficient data, which could be included in meta-analysis.
**6. Other bias**		
6.1 Conflict of interest	Definitely low	The authors declare that they have no known competing financial interests or personal relationships that could have appeared to influence the work reported in this paper.

RR, relative risk; OR, odds ratio; SMR, standard mortality rate; SIR, standard incidence rate; ERR, excess relative risk; HR, hazard ratio; CR, crude ratio; ASR, age standardized ratio; CI, confidence interval.

Table S10. Summary of the risk of bias of individual studies of workers in nuclear power plants according the NTP/OHAT risk of bias tool.

• Tier 1: A study must be rated as “definitely low” or “probably low” risk of bias for key elements and have most other applicable items answered “definitely low” or “probably low” risk of bias.

Example of key risk of bias elements for observational human studies:
o Can we be confident in the exposure characterization?
o Can we be confident in the outcome assessment?
o Does the study design or analysis account for important confounding variables?

• Tier 2: Study meets neither the criteria for 1st or 3rd tiers.
• Tier 3: A study must be rated as “definitely high” or “probably high” risk of bias for key elements and have most other applicable items answered “definitely high” or “probably high” risk of bias.

Symbol	Meaning	Symbol	Meaning
++	**Definitely low risk of bias**	+	**Probably low risk of bias**
- -	**Definitely high risk of bias**	-	**Probably high risk of bias**

**Domains**	**Rating**												
	**Jablon et al. 1993[4]**	**Auvinen et al. 2002[5]**	**Zablotska et al. 2004[6]**	**Kindler et al. 2006[7]**	**Vrijheid et al. 2007[8]**	**Cardis et al. 2007[9]**	**Jeong et al. 2010[10]**	**Laurent et al. 2010[11]**	**Merzenich et al. 2014[12]**	**Azizova et al. 2018[13]**	**Boice et al. 2022[14]**	**Kudo et al. 2022[15]**	**Mumma et al. 2022[16]**
**1. Selection bias**													
1.1 Did selection of study participants result in appropriate comparison groups?	++	++	++	++	++	++	++	++	++	++	++	++	++
**2. Confounding bias**													
2.1 Did the study design or analysis account for important confounding and modifying variables? **(key domain)**	-	+	++	++	-	-	++	++	+	++	-	+	+
**3. Attrition/Exclusion bias**													
3.1 Were outcome data complete without attrition or exclusion from analysis?	-	++	++	-	++	++	++	++	++	++	++	++	++
**4. Detection bias**													
4.1 Can we be confident in the exposure characterization? **(key domain)**	++	++	++	+	+	+	++	++	++	++	++	++	++
4.2 Can we be confident in the outcome assessment? **(key domain)**	+	+	++	+	++	+	++	++	++	++	++	++	++
**5. Selective reporting bias**													
5.1 Were all measured outcomes reported?	++	++	++	++	++	++	++	++	++	++	++	++	++
**6. Other bias**													
6.1 Conflict of interest	-	-	-	-	++	++	-	-	-	-	++	-	- -
**Summary tier**	**T 2**	**T 1**	**T 1**	**T 1**	**T 2**	**T 2**	**T 1**	**T 1**	**T 1**	**T 1**	**T 2**	**T 1**	**T 1**

NTP/OHAT, National Toxicology Program/Office of Health Assessment Translation.

Table S11. Summary of the risk of bias of individual studies of residents living near nuclear power plants according the NTP/OHAT risk of bias tool.

• Tier 1: A study must be rated as “definitely low” or “probably low” risk of bias for key elements and have most other applicable items answered “definitely low” or “probably low” risk of bias.

Example of key risk of bias elements for observational human studies:
o Can we be confident in the exposure characterization?
o Can we be confident in the outcome assessment?
o Does the study design or analysis account for important confounding variables?

• Tier 2: Study meets neither the criteria for 1st or 3rd tiers.
• Tier 3: A study must be rated as “definitely high” or “probably high” risk of bias for key elements and have most other applicable items answered “definitely high” or “probably high” risk of bias.

++	**Definitely low risk of bias**	+	**Probably low risk of bias**
- -	**Definitely high risk of bias**	-	**Probably high risk of bias**

Domains	Enstrom et al. 1983[17]	Baron et al. 1984[18]	Ewings et al. 1989[19]	Urquhart et al. 1991[20]	Michaelis et al. 1992[21]	Goldsmith et al. 1992[22]	McLaughlin et al. 1993[23]	Hattchouel et al. 1995[24]	Morris et al. 1996[25]	López-Abente et al. 1999[26]	López-Abente et al. 2001[27]	Silva-Mato et al. 2003[28]	Mangano et al. 2003[29]	White-Koning et al. 2004[30]	Yoshimoto et al. 2004[31]	Boice et al. 2005[32]	Kaatsch et al. 2008[33]
**1. Selection bias**																	
1.1 Did selection of study participants result in appropriate comparison groups?	++	++	++	++	++	++	++	++	++	++	++	++	++	++	++	++	++
**2. Confounding bias**																	
2.1 Did the study design or analysis account for important confounding and modifying variables? **(key domain)**	-	-	-	-	-	-	-	-	++	+	+	-	-	+	-	-	-
**3. Attrition/Exclusion bias**																	
3.1 Were outcome data complete without attrition or exclusion from analysis?	++	-	-	++	-	-	++	-	++	++	-	-	-	-	++	++	-
**4. Detection bias**																	
4.1 Can we be confident in the exposure characterization? **(key domain)**	++	++	++	++	++	++	++	++	++	++	++	++	++	++	++	++	++
4.2 Can we be confident in the outcome assessment? **(key domain)**	++	++	+	+	+	+	++	++	+	++	++	+	++	+	++	++	+
**5. Selective reporting bias**																	
5.1 Were all measured outcomes reported?	++	++	++	++	++	++	++	++	++	++	++	++	++	++	++	++	++
**6. Other bias**																	
6.1 Conflict of interest	-	-	-	-	-	-	-	-	-	-	-	-	-	-	-	-	++
**Summary tier**	**T 2**	**T 2**	**T 2**	**T 2**	**T 2**	**T 2**	**T 2**	**T 2**	**T 1**	**T 1**	**T 1**	**T 2**	**T 2**	**T 1**	**T 2**	**T 2**	**T 2**

Table S7. Continued.

Domains	Zadnik et al. 2008[34]	Spix et al. 2009[35]	Mangano et al. 2009[36]	Heinävaara et al. 2010[37]	Spycher et al. 2011[38]	Ma et al. 2011[39]	Bazyka et al. 2012[40]	Ahn et al. 2012[41]	Sermage-Faure et al. 2012[42]	Bithell et al. 2013[43]	Lane et al. 2013[44]	Bollaerts et al. 2014[45]	Wang et al. 2016[46]	Khomenko et al. 2017[47]	Demoury et al. 2017[48]	Desbiolles et al. 2018[49]	Demoury et al. 2021[50]
• Tier 1: A study must be rated as "definitely low" or "probably low" risk of bias for key elements and have most other applicable items answered "definitely low" or "probably low" risk of bias.<br><br>Example of key risk of bias elements for observational human studies:<br>o Can we be confident in the exposure characterization?<br>o Can we be confident in the outcome assessment?<br>o Does the study design or analysis account for important confounding variables?<br><br>• Tier 2: Study meets neither the criteria for 1st or 3rd tiers.<br>• Tier 3: A study must be rated as "definitely high" or "probably high" risk of bias for key elements and have most other applicable items answered "definitely high" or "probably high" risk of bias.<br>++ **Definitely low risk of bias**; + **Probably low risk of bias**; - - **Definitely high risk of bias**; - **Probably high risk of bias**	Rating																
**1. Selection bias**																	
1.1 Did selection of study participants result in appropriate comparison groups?	++	++	++	++	++	++	++	++	++	++	++	++	++	++	++	++	++
**2. Confounding bias**																	
2.1 Did the study design or analysis account for important confounding and modifying variables? **(key domain)**	-	+	-	+	+	-	-	++	+	-	-	-	-	-	+	++	++
**3. Attrition/Exclusion bias**																	
3.1 Were outcome data complete without attrition or exclusion from analysis?	++	++	++	++	++	-	++	++	++	++	-	-	-	-	-	++	-
**4. Detection bias**																	
4.1 Can we be confident in the exposure characterization? **(key domain)**	++	++	++	++	++	++	++	++	++	++	++	++	++	++	++	++	++
4.2 Can we be confident in the outcome assessment? **(key domain)**	+	+	+	++	++	++	++	++	+	++	++	++	++	++	+	++	++
**5. Selective reporting bias**																	
5.1 Were all measured outcomes reported?	++	++	++	++	++	++	++	++	++	++	++	++	++	++	++	++	++
**6. Other bias**																	
6.1 Conflict of interest	-	++	-	++	++	-	-	-	-	--	-	++	++	-	++	-	++
**Summary tier**	**T 2**	**T 1**	**T 2**	**T 1**	**T 1**	**T 2**	**T 2**	**T 1**	**T 1**	**T 2**	**T 2**	**T 2**	**T 2**	**T 2**	**T1**	**T 1**	**T 1**

NTP/OHAT, National Toxicology Program/Office of Health Assessment Translation.

Appendix 8 Sensitivity analysis

Table S12. Summary of sensitivity analysis of pooled estimates on meta-analysis random-effects model for all-cancer among workers in nuclear power plants.

Cause	Number of study groups	Pooled RR (95% CI)	Relative difference on average (%) (range)
**Cancers**			
**All-cancer**	20	0.85 (0.75–0.97)	1.3% (0.1%–3.9%)
**Brain and CNS cancer**	6	0.87 (0.75–1.02)	5.2% (0.2%–28.3%)
**Colon cancer**	7	0.95 (0.86–1.06)	0.9% (0%–4.5%)
**Esophageal cancer**	2	1.01 (0.34–3.03)	73.1% (32.8%–113.3%)
**Leukemia**	9	1.05 (0.91–1.21)	1.8% (0.1%–13%)
**Liver cancer**	4	0.42 (0.17–1.00)	16.1% (0.3%–26.1%)
**Lung cancer**	16	1.04 (0.85–1.27)	2.8% (0.2%–5.9%)
**Mesothelioma**	2	5.53 (4.05–7.54)	5.4% (0.5%–10.4%)
**Pancreatic cancer**	7	0.84 (0.50–1.42)	9.1% (1.5%–19.8%)
**Prostate cancer**	7	0.45 (0.18–1.16)	29.6% (4.3%–116%)
**Rectal cancer**	6	1.10 (0.59–2.08)	11.9% (6%–21.9%)
**Stomach cancer**	7	0.99 (0.83–1.18)	1.5% (0.1%–6.4%)
**Thyroid cancer**	5	2.29 (0.76–6.90)	26.4% (10.6%–38.3%)
**Non-cancer diseases**			
**Cerebrovascular disease**	5	0.92 (0.75–1.12)	5.5% (1%–10.8%)
**Circulatory disease**	6	0.93 (0.78–1.11)	3.7% (0.7%–6.7%)
**Digestive disease**	3	0.78 (0.39–1.59)	13.9% (0.8%–27.5%)
**Respiratory disease**	3	0.05 (0.001–2.47)	596.1% (77.5%–1624.2%)

CNS, central nervous system; mSv, millisievert; RR, relative risk; CI, confidence interval.

Table S13. Summary of sensitivity analysis of pooled estimates on meta-analysis random-effects model for all-cancer among residents living within 30 km of the nuclear power plants.

Cause	Number of study groups	Pooled RR (95% CI)	Relative difference on average (%) (range)
**All-cancer**	53	1.05 (1.00–1.09)	0.6% (0.0%–1.9%)
**Breast cancer**	19	1.01 (0.93–1.10)	1.4% (0.1%–4.8%)
**Leukemia**	102	1.09 (1.03–1.16)	0.2% (0.0%–1.2%)
**Lung cancer**	22	0.96 (0.86–1.08)	1.2% (0.0%–4.3%)
**Stomach cancer**	19	0.99 (0.85–1.16)	1.7% (0.1%–6.4%)
**Thyroid cancer**	27	1.17 (1.04–1.32)	1.2% (0.0%–2.6%)

CNS, central nervous system; mSv, millisievert; RR, relative risk; CI, confidence interval.

Appendix 9 Comparison with previous meta-analysis

Table S14. The comparison with previous meta-analyses.

Author	Present meta-analysis	Baker et al. (2007)[52]	Kim et al. (2016)[53]	Qu et al. (2018)[54]	Hauptmann et al. (2020)[55]	Visci et al. (2022)[56]
**Study population**	Workers in nuclear power plants<br><br>Residents living near nuclear power plants	Residents aged under 26 years living near nuclear installations	Residents living near nuclear power plants	Workers in the nuclear industry with a main occupation in mining, refining, enrichment, non-destructive testing and nuclear weapon research, but not in nuclear power plants, medical facilities, education or nuclear accidents	Populations exposed to low-dose ionizing radiation (mean cumulative dose <100 mGy).	Workers in the nuclear industry<br><br>Patients exposed to either Thorotrast (a contrast agent used for x-rays) or the EBRT
**Databases**	Cochrane Library<br>PubMed<br>ScienceDirect<br>Web of Science	Not specified	Medline<br>Embase	PubMed<br>Embase	Analyses included epidemiological studies published since the Biological Effects of Ionizing Radiation VII report in 2006 and before 2018.	PubMed<br>Scopus<br>Embase
**Keywords**	"((“nuclear power plant” OR “nuclear site” OR “nuclear power” OR “nuclear facility” OR “nuclear industry” OR “nuclear installation”) NOT (accident OR disaster OR “nuclear power plant incident”)) AND ((resident OR worker) OR (epidemiology OR incidence OR mortality OR death rate OR illness))”	Not specified	“nuclear facility”, “nuclear sites”, “nuclear industry”, “nuclear power plants”, “nuclear installations”, “radiation exposure”, “ionizing radiation”, “environmental exposure”, “thyroid neoplasm”, “thyroid cancer”, “thyroid carcinoma”	‘solid cancer’ OR ‘lung cancer’ OR ‘brain cancer’ OR ‘central nervous system cancer’ OR ‘liver cancer’ OR ‘stomach cancer’ OR ‘colorectal cancer’ OR ‘colon cancer’ OR ‘intestinal cancer’ OR ‘rectum cancer’ OR ‘kidney cancer’ OR ‘bladder cancer’ OR ‘prostate cancer’ AND ‘mortality’ AND ‘nuclear industry’ OR ‘nuclear facility’	Not specified	(Radiotherapy OR EBRT OR “external beam radiotherapy” OR “stereotactic radiotherapy” OR (peritoneal AND irradiation) OR radionuclides OR “therapeutic ionizing radiation”) OR ((Nuclear AND (industry * OR work OR worker * OR job)) OR (Radiation AND (industry * OR work OR worker * OR job)) OR “Radiography / adverse effects” [Mesh] OR (hiroshima [tiab] OR (nagasaki [tiab] OR atomic bomb survivors OR life span study) OR “Thorium Dioxide [Mesh] OR Thorotrast”.<br>AND<br>(“Etiology” [Subheading] OR etiologic * OR Neoplasm, Radiation-Induced [MH] OR Neoplasms, Radiation-Induced * OR Neoplasms, Second Primary [MH] OR Neoplasms, Second Primary * OR etiology [MH] OR aetiologic * OR aetiology OR Cohort Studies [MH])))

Author	Present meta-analysis	Baker et al. (2007)[52]	Kim et al. (2016)[53]	Qu et al. (2018)[54]	Hauptmann et al. (2020)[55]	Visci et al. (2022)[56]
						AND<br>“Mesothelioma” OR “pleural cancer / neoplasm” OR “peritoneal cancer / neoplasm”
**Searching period**	Before January 13, 2023	Not specified	Before March 2015	Between January 1, 2000 and December 31, 2016	Not specified	Before June 2021
**Guideline**	PRISMA<br>Cochrane	No	No	No	No	PRISMA
**Exposure definition**	Occupational whole-body ionizing radiation exposure<br><br>Distance or geographic location to the nuclear power plants	Distance or geographic location to the nuclear installations	Distance or geographic location to the nuclear power plants	Whole-body ionizing radiation exposure (with a cumulative mean dose of <0.5 Sv/year, or at a low dose rate [<10 mSv/day])	Exposed to low-dose ionizing radiation (mean cumulative dose <100 mGy)	Therapeutic exposure:<br>Thorotrast (a contrast agent used for x-rays) or the EBRT<br><br>Occupational exposure:<br>Gamma ray, neutrons, uranium, plutonium, tritium, and other radionuclides in nuclear industry
**Exposure range**	For workers:<br>5–200 mSv<br><br>For residents:<br>1.56-29 km (comparison areas: more than 30 km)	0–16 km	0–30 km (comparison areas: more than 30 km or 50–100 km)	<0.5 Sv/year,<br>or<br><10 mSv/day	Mean cumulative dose <100 mGy	No
**Outcome definition**	For workers:<br>All-cancer, 12 kinds of cancers, as follows: brain and CNS cancer, colon cancer, esophageal cancer, leukemia, liver cancer, lung cancer, mesothelioma, pancreatic cancer, prostate cancer, rectal cancer, stomach cancer, thyroid cancer, and four kinds of non-cancer diseases, as follows: cerebrovascular disease, circulatory disease, digestive disease, and respiratory disease.<br><br>For residents:<br>All-cancer, five kinds of cancers, as follows: breast cancer, leukemia, lung cancer,	Leukemia	Thyroid cancer	Nine kinds of cancers, as follows: cancers of the lung, brain and CNS, liver, stomach, colorectum, kidney, bladder and prostate.	All-malignant neoplasms<br>Leukemia	Mesothelioma

Author	Present meta-analysis	Baker et al. (2007)[52]	Kim et al. (2016)[53]	Qu et al. (2018)[54]	Hauptmann et al. (2020)[55]	Visci et al. (2022)[56]
	stomach cancer, and thyroid cancer.					
**Exclusion criteria**	1. The studies did not focus on occupational or environmental exposure to nuclear power plants in normal operation.<br>2. The studies with overlapping populations, we selected the study with the longest study period.<br>3. The studies no effect measures or sufficient data.<br>4. The studies focused on inheritance, genetics, or cell biology.<br>5. The studies of reviews, letters, or conference abstracts.	1. Restricted in cohort studies.<br>2. The study did not include at least two of the following three variables: observed, expected, or end point (SIR or SMR) for individual nuclear sites, as opposed to a summarization that includes multiple sites.<br>3. The study did not have at least one age category less than 26 (only ages less than 26 were used in the meta-analysis).<br>4. The study did not indicate geographical zones in which cases or deaths occurred.	1. Studies that reported results from the Chernobyl or Fukushima accidents, fallout, any accident from unusual operation, or nuclear weapons testing sites were excluded.<br>2. If there was more than one article with the same or overlapping population, preference was given to the article providing more comprehensive, updated information.<br>3. If the article type was a review, letter, or comments, the study was excluded.<br>4. The studies that did not provide quantitative effect estimates about thyroid cancer.	Data were excluded for all reviews, books and reports where workers were engaged in their activities for <1 year, and from all articles containing insufficient/incomplete data.	1. Studies with the hypothesis that the median ERR equals zero<br>2. Studies with potential positive bias	1. Studies with wrong outcome<br>2. Studies with wrong exposure<br>3. Studies of reviews and case report<br>4. Studies with lack of risk measures
**Types of included studies**	Cohort study, case-control study, and ecologic study	Cohort study	Cohort study	Cohort study	Cohort study	Cohort study and case-control study
**Quality assessment**	NTP/OHAT	No	MOOSE	No	No	A checklist drawn up on the basis of the NIH indications was used.
**Number of included studies**	47 studies (13 on workers; 34 on residents)	17 studies	13 studies	27 studies	26 studies (eight for environmental, four for medical, and 14 for occupational exposure)	Nine studies (four on radiation therapy; five on nuclear worker)
**Publication years of included studies**	For workers:<br>1993–2022<br><br>For residents:<br>1983–2021	1984–1999	1993–2014	2000–2016	2006–2017	For workers:<br>1998–2016<br><br>For radiation therapy:<br>2002–2017
**Study period of included studies**	For workers:<br>1947–2011	1950–1993	1950–2010	1942–2008	1948–2012	For workers:<br>1946–2015

Author	Present meta-analysis	Baker et al. (2007)[52]	Kim et al. (2016)[53]	Qu et al. (2018)[54]	Hauptmann et al. (2020)[55]	Visci et al. (2022)[56]
	For residents:<br>1959–2016					For radiation therapy:<br>1965–2014
**Population of included studies**	480,623 workers and 7,530,886 residents	Not specified	2,298 cases	667,729 workers	Not specified	285,689 workers and 1,276,774 patients
**Statistical analysis**	Random-effect models<br>Meta-regression	Fixed-effect models<br>Random-effect models	Random-effect models	Random-effect models	Meta-ERR estimation	Random-effect models
**Subgroup analysis**	For workers:<br>◦ Geographic area<br>◦ Average annual cumulative dose of radiation exposure<br>◦ Definition of comparators<br>◦ Age at the end of follow-up<br>◦ Risk of bias<br><br>For residents:<br>◦ Geographic area<br>◦ Age of study population<br>◦ Sex<br>◦ Study types<br>◦ Definition of exposure indicators<br>◦ Distance from nuclear power plants<br>◦ Risk of bias	◦ Age<br>◦ Geographic zone	◦ Region<br>◦ Gender<br>◦ Exposure definition<br>◦ Reference population	Not performed	Not performed	Not performed
**Test of heterogeneity**	I-squared statistics	Chi-square tests	Chi-square tests<br>I-squared statistics	Chi-square tests<br>I-squared statistics	Chi-square tests<br>I-squared statistics	I-squared statistics
**Sensitivity analysis**	Yes	No	Yes	Yes	No	No
**Publication bias**	Egger's tests<br>Funnel plots	Funnel plots	Begg's and Egger's tests<br>Funnel plots	Begg's and Egger's tests<br>Funnel plots	No	Egger's tests<br>Funnel plots
**The certainty of evidence**	GRADE	No	No	No	No	No

EBRT, external beam radiation therapy; PRISMA, Preferred Reporting Items for Systematic Reviews and Meta-Analyses; Sv, sievert; mSv/day, millisievert per year; km, kilometer; mSv/year, millisievert per year; mGy, milligray; CNS, central nervous system; SIR, standardized incidence rate; SMR, standardized mortality rate; ERR, excess relative risk; NTP/OHAT, National Toxicology Program/Office of Health Assessment Translation; MOOSE, Meta-analysis Of Observational Studies in Epidemiology; NIH, National Institutes of Health; GRADE, Grading of Recommendations Assessment, Development, and Evaluation

Appendix 10 Funnel plot

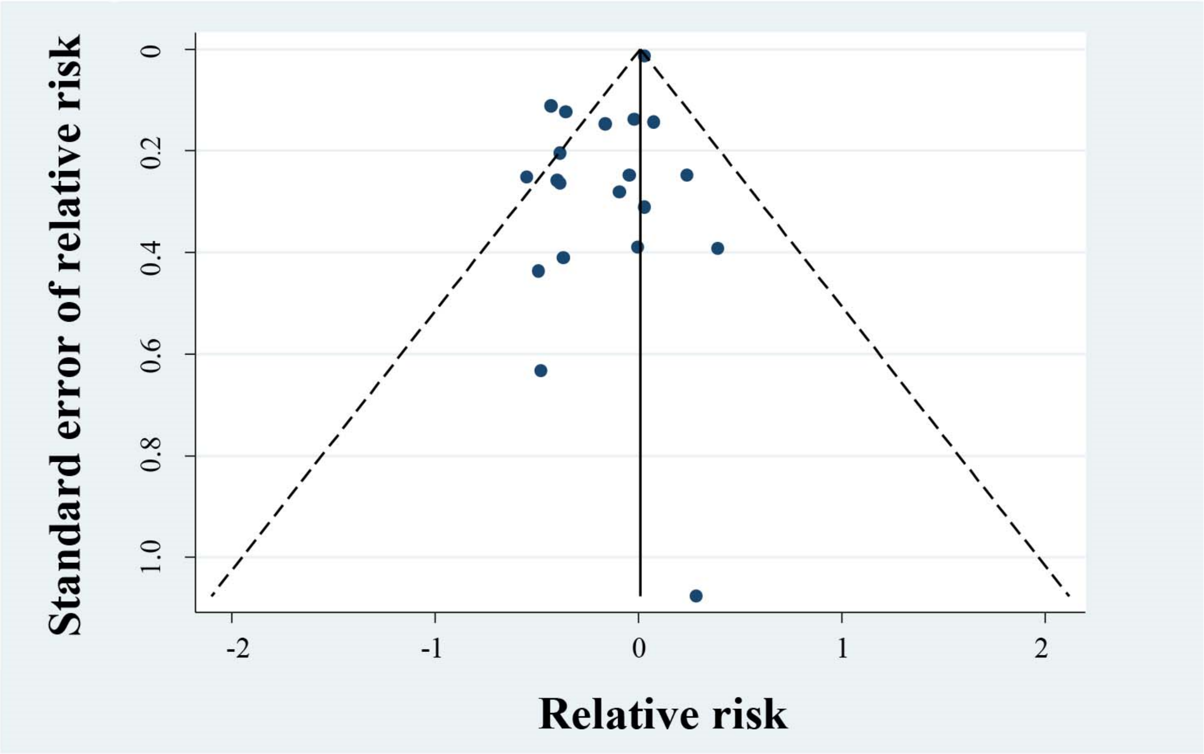

Figure S5. Funnel plot of publication bias of individual studies for all-cancer among workers in nuclear power plants (n = 20).
n, number of study groups included in meta-analysis.

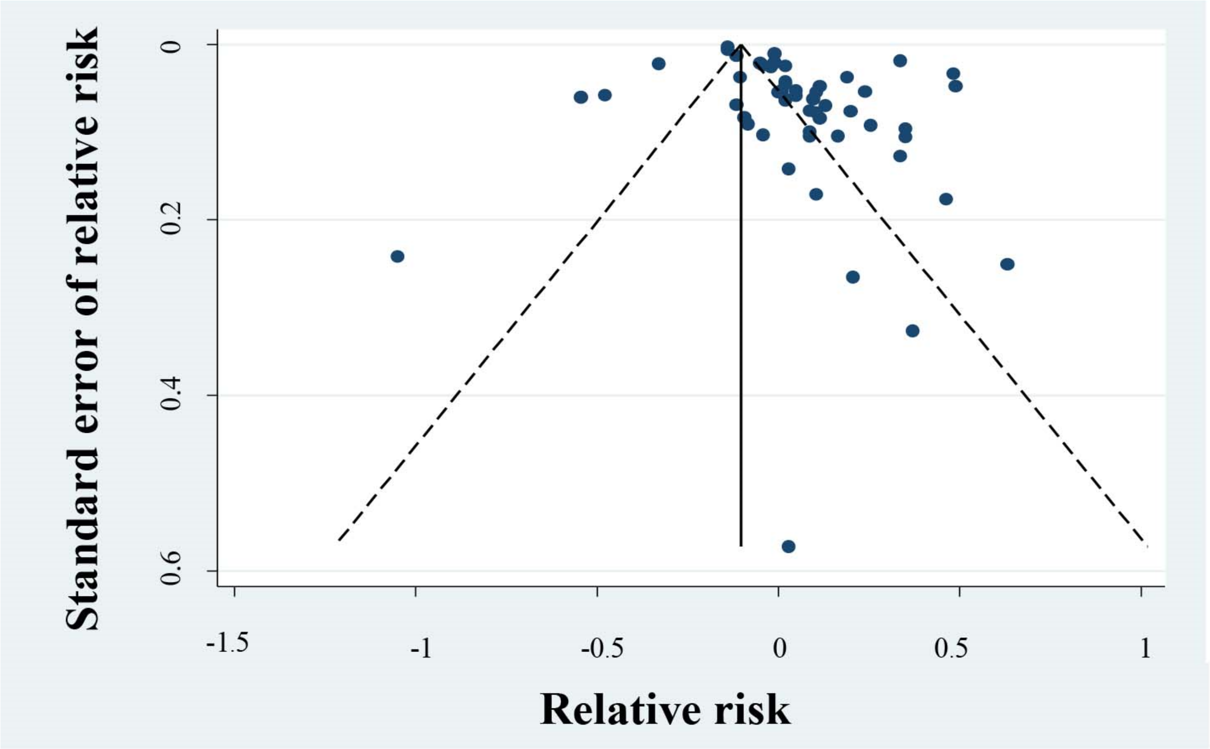

Figure S6. Funnel plot of publication bias of individual studies for all-cancer among residents living within 30 km of nuclear power plants (n = 53).
n, number of study groups included in meta-analysis.

References

1. Higgins JPT, Thomas J, Chandler J, Cumpston M, Li T, Page MJ. Cochrane handbook for systematic reviews of interventions. 2nd ed: Chichester: John Wiley & Sons; 2019.

2. Morris JA, Gardner MJ. Calculating confidence intervals for relative risks (odds ratios) and standardised ratios and rates. Br Med J (Clin Res Ed). 1988;296(6632):1313–6.

3. Zhang J, Kai F Y. What's the relative risk? A method of correcting the odds ratio in cohort studies of common outcomes. JAMA. 1998;280(19):1690–1.

4. Jablon S, Boice JD. Mortality among workers at a nuclear power plant in the United States. Cancer Causes Control. 1993;4:427–30.

5. Auvinen A, Pukkala E, Hyvönen H, Hakama M, Rytömaa T. Cancer incidence among Finnish nuclear reactor workers. J Occup Environ Med. 2002;44:634–8.

6. Zablotska LB, Ashmore JP, Howe GR. Analysis of mortality among Canadian nuclear power industry workers after chronic low-dose exposure to ionizing radiation. Radiat Res. 2004;161(6):633–41.

7. Kindler S, Roser M, Below H, Hoffmann W, Kohlmann T, Kramer A, et al. Thyroid disorders in employees of a nuclear power plant. Thyroid. 2006;16(10):1009–17.

8. Vrijheid M, Cardis E, Blettner M, Gilbert E, Hakama M, Hill C, et al. The 15-country collaborative study of cancer risk among radiation workers in the nuclear industry: design, epidemiological methods and descriptive results. Radiat Res. 2007;167(4):361–79.

9. Cardis E, Vrijheid M, Blettner M, Gilbert E, Hakama M, Hill C, et al. The 15-country collaborative study of cancer risk among radiation workers in the nuclear industry: estimates of radiation-related cancer risks. Radiat Res. 2007;167(4):396–416.

10. Jeong M, Jin YW, Yang KH, Ahn YO, Cha CY. Radiation exposure and cancer incidence in a cohort of nuclear power industry workers in the Republic of Korea, 1992–2005. Radiat Environ Biophys. 2010;49:47–55.

11. Laurent O, Metz-Flamant C, Rogel A, Hubert D, Riedel A, Garcier Y, et al. Relationship between occupational exposure to ionizing radiation and mortality at the French electricity company, period 1961–2003. Int Arch Occup Environ Health. 2010;83:935–44.

12. Merzenich H, Hammer GP, Tröltzsch K, Ruecker K, Buncke J, Fehringer F, et al. Mortality risk in a historical cohort of nuclear power plant workers in Germany: results from a second follow-up. Radiat Environ Biophys. 2014;53:405–16.

13. Azizova TV, Batistatou E, Grigorieva ES, McNamee R, Wakeford R, Liu H, et al. An assessment of radiation-associated risks of mortality from circulatory disease in the cohorts of Mayak and Sellafield nuclear workers. Radiat Res. 2018;189(4):371–88.

14. Boice JD, Jr., Cohen SS, Mumma MT, Hagemeyer DA, Chen H, Golden AP, et al. Mortality from leukemia, cancer and heart disease among U.S. nuclear power plant workers, 1957–2011. Int J Radiat Biol. 2022;98(4):657–78.

15. Kudo Si, Nishide A, Furuta H, Ishizawa N, Saigusa S. A risk comparison between lifestyle, socioeconomic status, and radiation: a cohort study of cancer mortality among Japanese nuclear workers (J-EPISODE). Health Phys. 2022;122(4):469–79.

16. Mumma MT, Sirko JL, Boice JD, Jr., Blot WJ. Mesothelioma mortality within two radiation monitored occupational cohorts. Int J Radiat Biol. 2022;98(4):786–94.

17. Enstrom JE. Cancer mortality patterns around the San Onofre nuclear power plant, 1960–1978. Am J Public Health. 1983;73(1):83–92.

18. Baron JA. Cancer mortality in small areas around nuclear facilities in England and Wales. Br J Cancer. 1984;50(6):815–24.

19. Ewings PD, Bowie C, Phillips MJ, Johnson SA. Incidence of leukaemia in young people in the vicinity of Hinkley Point nuclear power station, 1959–86. BMJ. 1989;299(6694):289–93.

20. Urquhart JD, Black RJ, Muirhead MJ, Sharp L, Maxwell M, Eden OB, et al. Case-control study of leukaemia and non-Hodgkin's lymphoma in children in Caithness near the Dounreay nuclear installation. BMJ. 1991;302(6778):687–92.

21. Michaelis J, Keller B, Haaf G, Kaatsch P. Incidence of childhood malignancies in the vicinity of west German nuclear power plants. Cancer Causes Control. 1992;3:255–63.

22. Goldsmith JR. Nuclear installations and childhood cancer in the UK: mortality and incidence for 0–9-year-old children, 1971–1980. Sci Total Environ. 1992;127(1-2):13–35.

23. McLaughlin JR, Clarke EA, Nishri ED, Anderson TW. Childhood leukemia in the vicinity of Canadian nuclear facilities. Cancer Causes Control. 1993;4:51–8.

24. Hattchouel JM, Laplanche A, Hill C. Leukaemia mortality around French nuclear sites. Br J Cancer. 1995;71(3):651–3.

25. Morris MS, Knorr RS. Adult leukemia and proximity-based surrogates for exposure to Pilgrim plant's nuclear emissions. Arch Environ Health. 1996;51(4):266–74.

26. López-Abente G, Aragonés N, Pollán M, Ruiz M, Gandarillas A. Leukemia, lymphomas, and myeloma mortality in the vicinity of nuclear power plants and nuclear fuel facilities in Spain. Cancer Epidemiol Biomarkers Prev. 1999;8(10):925–34.

27. López-Abente G, Aragonés N, Pollán M. Solid-tumor mortality in the vicinity of uranium cycle facilities and nuclear power plants in Spain. Environ Health Perspect. 2001;109(7):721–9.

28. Silva-Mato A, Viana D, Fernández-SanMartín MI, Cobos J, Viana M. Cancer risk around the nuclear power plants of Trillo and Zorita (Spain). Occup Environ Med. 2003;60(7):521–7.

29. Mangano JJ, Sherman J, Chang C, Dave A, Feinberg E, Frimer M. Elevated childhood cancer incidence proximate to U.S. nuclear power plants. Arch Environ Health. 2003;58(2):74–82.

30. White-Koning ML, Hémon D, Laurier D, Tirmarche M, Jougla E, Goubin A, et al. Incidence of childhood leukaemia in the vicinity of nuclear sites in France, 1990–1998. Br J Cancer. 2004;91(5):916–22.

31. Yoshimoto Y, Yoshinaga S, Yamamoto K, Fijimoto K, Nishizawa K, Sasaki Y. Research on potential radiation risks in areas with nuclear power plants in Japan: leukaemia and malignant lymphoma mortality between 1972 and 1997 in 100 selected municipalities. J Radiol Prot. 2004;24(4):343–68.

32. Boice JD, Mumma MT, Blot WJ, Heath CW. Childhood cancer mortality in relation to the St Lucie nuclear power station. J Radiol Prot. 2005;25(3):229–40.

33. Kaatsch P, Spix C, Jung I, Blettner M. Childhood leukemia in the vicinity of nuclear power plants in Germany. Dtsch Arztebl Int. 2008;105(42):725–32.

34. Zadnik V, Žagar T, Drobne S, Žakelj MP. Estimation of cancer burden in Brežice municipality, a community neighboring Krško nuclear power plant in Slovenia. Croat Med J. 2008;49(2):257–66.

35. Spix C, Schulze-Rath R, Kaatsch P, Blettner M. Case-control study on risk factors for leukaemia and brain tumours in children under 5 years in Germany. Klin Padiatr. 2009;221(6):362–8.

36. Mangano JJ. Geographic variation in U.S. thyroid cancer incidence and a cluster near nuclear reactors in New Jersey, New York, and Pennsylvania. Int J Health Serv. 2009;39(4):643–61.

37. Heinävaara S, Toikkanen S, Pasanen K, Verkasalo PK, Kurttio P, Auvinen A. Cancer incidence in the vicinity of Finnish nuclear power plants: an emphasis on childhood leukemia. Cancer Causes Control. 2010;21:587–95.

38. Spycher BD, Feller M, Zwahlen M, Röösli M, von der Weid NX, Hengartner H, et al. Childhood cancer and nuclear power plants in Switzerland: a census-based cohort study. Int J Epidemiol. 2011;40(5):1247–60.

39. Ma F, Lehnherr M, Fornoff J, Shen T. Childhood cancer incidence in proximity to nuclear power plants in Illinois. Arch Environ Occup Health. 2011;66(2):87–94.

40. Bazyka DA, Prysyazhnyuk AY, Romanenko AY, Fedorenko ZP, Gudzenko NA, Fuzik MM, et al. Cancer

incidence and nuclear facilities in Ukraine: a community-based study. Exp Oncol. 2012;34:116–20.

41. Ahn YO, Li ZM. Cancer risk in adult residents near nuclear power plants in Korea-a cohort study of 1992–2010. J Korean Med Sci. 2012;27(9):999–1008.

42. Sermage-Faure C, Laurier D, Goujon-Bellec S, Chartier M, Guyot-Goubin A, Rudant J, et al. Childhood leukemia around French nuclear power plants--the Geocap study, 2002–2007. Int J Cancer. 2012;131(5):E769–80.

43. Bithell JF, Murphy MF, Stiller CA, Toumpakari E, Vincent T, Wakeford R. Leukaemia in young children in the vicinity of British nuclear power plants: a case-control study. Br J Cancer. 2013;109(11):2880–5.

44. Lane R, Dagher E, Burtt J, Thompson PA. Radiation exposure and cancer incidence (1990 to 2008) around nuclear power plants in Ontario, Canada. J Environ Prot 2013;4:888–913.

45. Bollaerts K, Fierens S, Van Bladel L, Simons K, Sonck M, Poffijn A, et al. Thyroid cancer incidence in the vicinity of nuclear sites in Belgium, 2000–2008. Thyroid. 2014;24(5):906–17.

46. Wang SI, Yaung CL, Lee LT, Chiou SJ. Cancer incidence in the vicinity of nuclear power plants in Taiwan: a population-based study. Environ Sci Pollut Res. 2016;23:571–80.

47. Khomenko IM, Zakladna NV, Orlova NM. Population morbidity in the Zaporizhzhia nuclear power plant observation zone as an integral part of public health. Probl Radiac Med Radiobiol. 2017;22:162–71.

48. Demoury C, De Smedt T, De Schutter H, Sonck M, Van Damme N, Bollaerts K, et al. Thyroid cancer incidence around the Belgian nuclear sites, 2000–2014. Int J Environ Res Public Health. 2017;14(9):988.

49. Desbiolles A, Roudier C, Goria S, Stempfelet M, Kairo C, Quintin C, et al. Cancer incidence in adults living in the vicinity of nuclear power plants in France, based on data from the French Network of Cancer Registries. Int J Cancer. 2018;142(5):899–909.

50. Demoury C, Faes C, De Schutter H, Carbonnelle S, Rosskamp M, Francart J, et al. Childhood leukemia near nuclear sites in Belgium: an ecological study at small geographical level. Cancer Epidemiol. 2021;72:101910.

51. National Toxicology Program/Office of Health Assessment and Translation (NTP/OHAT). Handbook for conducting a literature-based health assessment using OHAT approach for systematic review and evidence integration. Washington, DC: National Toxicology Program; 2019.

52. Baker PJ, Hoel DG. Meta-analysis of standardized incidence and mortality rates of childhood leukaemia in proximity to nuclear facilities. Eur J Cancer Care. 2007;16(4):355–63.

53. Kim J, Bang Y, Lee WJ. Living near nuclear power plants and thyroid cancer risk: a systematic review and meta-analysis. Environ Int. 2016;87:42–8.

54. Qu SG, Gao J, Tang B, Yu B, Shen YP, Tu Y. Low-dose ionizing radiation increases the mortality risk of solid cancers in nuclear industry workers: a meta-analysis. Mol Clin Oncol. 2018;8(5):703–11.

55. Hauptmann M, Daniels RD, Cardis E, Cullings HM, Kendall G, Laurier D, et al. Epidemiological studies of low-dose ionizing radiation and cancer: summary bias assessment and meta-analysis. J Natl Cancer Inst, Monogr. 2020;2020(56):188–200.

56. Visci G, Rizzello E, Zunarelli C, Violante FS, Boffetta P. Relationship between exposure to ionizing radiation and mesothelioma risk: a systematic review of the scientific literature and meta-analysis. Cancer Med. 2022;11(3):778–89.
